# Principles and priorities for integrated tuberculosis screening and care: A modified Delphi consensus exercise

**DOI:** 10.1371/journal.pgph.0005954

**Published:** 2026-03-02

**Authors:** Claire Jacqueline Calderwood, Tenzin Kunor, Mikaela Coleman, Edson Marambire, Uzma Khan, Rosa Herrera, Jeffry Acaba, Zafar Hussain, Diptendu Bhattacharya, Leyla Larsson, Jose Luis Paredes, Laura Paramo, Márcia Chiluvane, Thu-Anh Nguyen, Hanh Nguyen Hong, Marc d’Elbée, Eneyi Kpokiri, Luan Nguyen Quang Vo, Tom Wingfield, Ben Marais, Madhavi Bhargava, Sarah Bernays, Katharina Kranzer

**Affiliations:** 1 Clinical Research Department, London School of Hygiene & Tropical Medicine, London, United Kingdom; 2 The Health Research Unit Zimbabwe, Biomedical Research and Training Institute, Harare, Zimbabwe; 3 National Heart and Lung Institute, Imperial College London, London, United Kingdom,; 4 We Are TB, Georgia, United States of America, Atlanta; 5 Institute of Infectious Diseases and Tropical Medicine, LMU University Hospital, LMU Munich, Munich, Germany; 6 German Center for Infection Research (DZIF), partner site Munich, Munich, Germany; 7 Sydney Infectious Diseases Institute, University of Sydney, Sydney, Australia; 8 CIH^**LMU**^ Center for International Health, University Hospital, LMU Munich, Munich, Germany; 9 Department of Epidemiology, Biostatistics and Occupational Health, McGill University, Montréal, Canada; 10 Interactive Research and Development (IRD) Global, Atlanta, Singapore; 11 TB People, Ottawa, Ontario, Canada; 12 Autonomous University of Durango, Mexicali Campus, Mexicali, Mexico; 13 APCASO, Bangkok, Thailand; 14 Survivors Against TB, Delhi, India; 15 Department of Internal Medicine, Advocate Illinois Masonic Medical Center, Chicago, Illinois, United States of America; 16 Instituto Nacional de Saúde (INS), Maputo, Mozambique; 17 Sydney School of Public Health, University of Sydney, Sydney, Australia; 18 University of Sydney Vietnam Institute, Ho Chi Minh City, Vietnam; 19 Oxford University Clinical Research Unit (OUCRU), Ho Chi Minh City, Vietnam; 20 University of Bordeaux, National Institute for Health and Medical Research, Research Institute for Sustainable Development, Bordeaux, France; 21 Friends for International TB Relief, Ha Noi, Viet Nam; 22 Department of Global Public Health, Karolinska Institutet, Stockholm, Sweden; 23 Centre for Tuberculosis Research, Departments of Clinical Sciences and International Public Health, Liverpool School of Tropical Medicine, Liverpool, United Kingdom; 24 Tropical and Infectious Diseases Unit, Liverpool University Hospitals NHS Foundation Trust, Liverpool, United Kingdom; 25 Department of Community Medicine, Yenepoya Medical College, Mangalore, India; 26 Center for Nutrition Studies, Yenepoya, Mangalore, India; University of Ottawa Faculty of Medicine, CANADA

## Abstract

Tuberculosis predominates in communities with multiple health and socioeconomic vulnerabilities. Tuberculosis diagnosis presents an opportunity for expanded health services to tuberculosis-affected households. We conducted a modified Delphi process to ascertain if and how expanded services should be offered to people with tuberculosis and their households. Purposively invited panellists were identified through professional networks and included researchers, service providers, policymakers and members of tuberculosis-affected communities. Panellists completed two online survey rounds. Round one sought to establish consensus on the perceived value of integration and capture diverse views on service integration priorities through free-text responses. Round two explored broad consensus statements (consensus defined as ≥75% agreement) developed from round one responses using Likert scales and country-specific priorities. Free-text responses were analysed using inductive thematic analysis. The percentage of panellists agreeing with each statement was calculated as a proportion of all responses, overall and by pre-specified subgroups of professional categories and WHO region. In round one, 223 panellists from 44 countries indicated strong support for expanded and better integrated services for people with tuberculosis (98% agreement), and their household contacts (84%). In round two, 324 people from 68 countries reached consensus on key motivations for service integration. These included improved tuberculosis treatment and other health outcomes among people with tuberculosis, and increased tuberculosis screening and preventive treatment uptake among contacts. Almost all (>99%) panellists agreed that people with tuberculosis should be routinely screened for relevant non-tuberculosis conditions, but only 69% thought this was appropriate among household contacts. There was consensus (93%) that population-wide tuberculosis screening should be integrated with other disease screening. Multiple, often context-specific, considerations for implementation were highlighted. Integrated tuberculosis screening and care is highly valued by global tuberculosis experts. This international consensus provides a strong mandate for research evaluating the feasibility and effectiveness of integrated tuberculosis service delivery and further policy and guideline development.

## Background

Tuberculosis is the leading cause of death due to a single infectious disease globally [1]. Drivers of tuberculosis include human immunodeficiency virus (HIV), diabetes, undernutrition, smoking, alcohol use and air pollution, whilst comorbidities worsen tuberculosis treatment outcomes and long-term health [2]. Since 2004, the World Health Organization (WHO) has recommended integrated tuberculosis and HIV care; this is now well-evidenced, well-established, and included in routine monitoring frameworks [3]. More recent recommendations endorse integrated care of other comorbidities (e.g., undernutrition, diabetes and mental health), whilst highlighting the limited volume and low quality of available evidence [4–6]. Consequent on limited evaluation data implementation of integrated tuberculosis services is highly variable, with no standardised service package, financing or reporting [7,8].

Household contacts of people with tuberculosis are also at higher risk of tuberculosis comorbidities such as HIV and undernutrition, and risk behaviours such as cigarette smoking and alcohol use [9–11]. Household contacts are impacted by the devastating loss of livelihood and stigma caused by tuberculosis, whilst playing a critical role in caring for the person affected [12]. Systematic screening for tuberculosis among household contacts, a key component of the WHO End TB strategy, may present an opportunity to provide services that mitigate the impact of tuberculosis on families (including through prevention of further tuberculosis episodes), and improve overall health [13,14]. WHO recommend HIV testing for household contacts in high HIV prevalence settings. Outside this, however, integrated screening of household contacts has received limited attention [15–17].

Progress on integration of services into tuberculosis screening and care is contingent on consensus to inform intervention development, evidence generation and policy recommendations. This modified Delphi exercise sought to establish global consensus from tuberculosis experts on integrated tuberculosis screening and care.

## Methods

### Ethics statement

This study was approved by the ethics committee of the London School of Hygiene & Tropical Medicine (28783) and reported following ACCORD (ACcurate COnsensus Reporting Document) guidance (Table 1 in S1 Appendix) [18]. All panellists provided electronic written informed consent; responses were anonymised before analysis. No incentives were offered. Detailed rationale, methods, and literature searches, and a glossary of terms are provided in Table 2-7 in S1 Appendix.

### Study design

**Fig 1:**
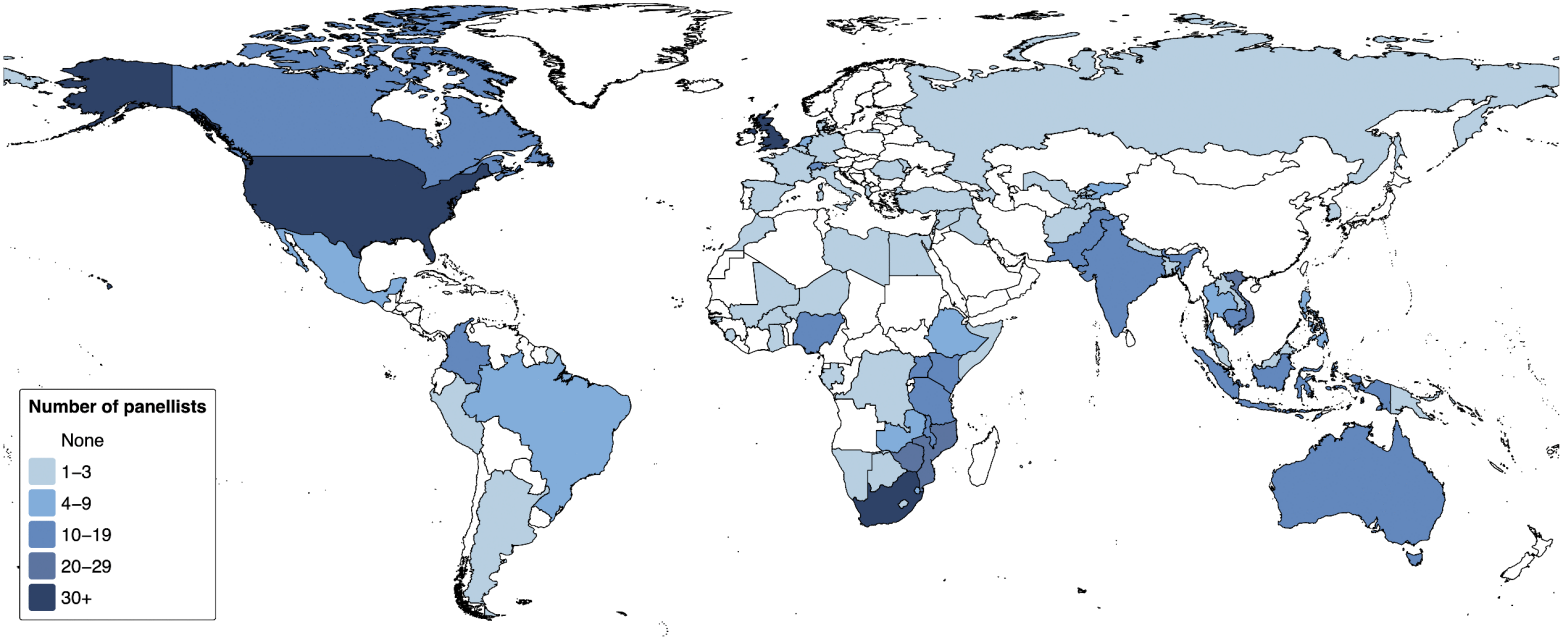
Countries in which Delphi panellists lived and worked (n = 547 individual responses across two rounds). *Panellists who responded to both rounds are represented twice. 31 panellists working across multiple countries are not included. Country borders based on World Bank data: https://datacatalog.worldbank.org/search/dataset/0038272/World-Bank-Official-Boundaries (Creative Commons Attribution 4.0 licence).

We employed a modified Delphi process [18]. Surveys were disseminated and hosted online in up to 5 languages to gain input from a diverse and globally representative group of professionals including researchers, policymakers, representatives of National Tuberculosis Programmes (NTPs), healthcare workers, civil society and affected community (Fig 1 in S1 Appendix). The round 1 survey (data collection: 11th July 2023–5th September 2023) explored consensus with the broad principle of integration, which conditions should be considered for integration, and aimed to synthesise views on the rationale for and opportunities and challenges of integrated tuberculosis service delivery, applied to both people with tuberculosis and household contacts.

The round 2 survey (10th May 2024–20th July 2024) included both round 1 panellists and other people purposively invited to increase geographic and professional group diversity (Supplementary methods). In round 2, consensus statements were proposed following an anonymised summary of round 1 findings. Statements were developed from round 1 findings with input from two steering committees (each including researchers or members of tuberculosis-affected community). The round 2 survey measured expert consensus with each proposed statement using a 5-point Likert scale (strongly agree to strongly disagree). Panellists could leave any statement on which they did not feel able to comment unanswered. This round also asked panellists to name up to three countries in which they were familiar with the tuberculosis screening and/or care programme; answer questions on current services in these settings; and rank conditions for integration in order of priority. They had the option to leave a condition unranked if they did not consider it important. The primary outcome of the study was consensus, prespecified as >75% agreement [19]. Secondary outcomes were i) ranking of conditions and ii) understanding of the implementation context into which integrated services are being proposed, including health system barriers and requirements.

### Data analysis

Thematic analysis of free-text responses (CJC, MC, TK) employed an inductive approach. The percentage of panellists agreeing (selecting agree/strongly agree) and disagreeing (disagree/strongly disagree) were calculated for each statement. Votes of “neither agree nor disagree” were included in the denominator [18]. Rankings were combined across panellists by assigning a score of 1 (highest) to 12 (lowest) and an arbitrary value of 22 to conditions left blank, before summing scores. Pre-specified subgroups were professional categories and WHO regions. As a sensitivity analysis, weighted average percentage agreement was calculated, giving equal weight to each professional category, world region, or country.

### Patient and public involvement

The researchers directing the exercise had mixed expertise, including qualitative and quantitative methods, with and without lived experience of tuberculosis. We aimed for diverse input across relevant professions and world regions, including specific efforts to capture the voices of members of tuberculosis-affected community (S1 Appendix). Two advisory committees comprised of a) tuberculosis researchers (EM, LNQV, TW, BM, MB, SB) and b) tuberculosis survivors, many of whom also lived with tuberculosis co-morbidities, (UK, RH, JA, ZH, DB) oversaw the research, including development of this manuscript.

## Results

### Panellist characteristics

In round 1, 644 people and subscribers to 15 mailing lists were invited to participate; 223 people across 44 countries participated (Table 8 in S1 Appendix). In round 2, 499 people and subscribers to 13 mailing lists were invited; 324 people across 68 countries participated. 124 people completed both rounds (55.6% of round 1 panellists completed round 2). Across both rounds, 47% of participants were women, 19% were affiliated with National TB Programmes or Ministries of Health, 17% with a healthcare providing organisation, 35% with non-governmental organisations and 50% with academic or research organisations. Six percent of panellists identified themselves as tuberculosis survivors. Panellists responded from 44 countries in round 1 and 68 countries in round 2, with representation of six continents (Fig 1).

### Survey round 1 findings

**Fig 2:**
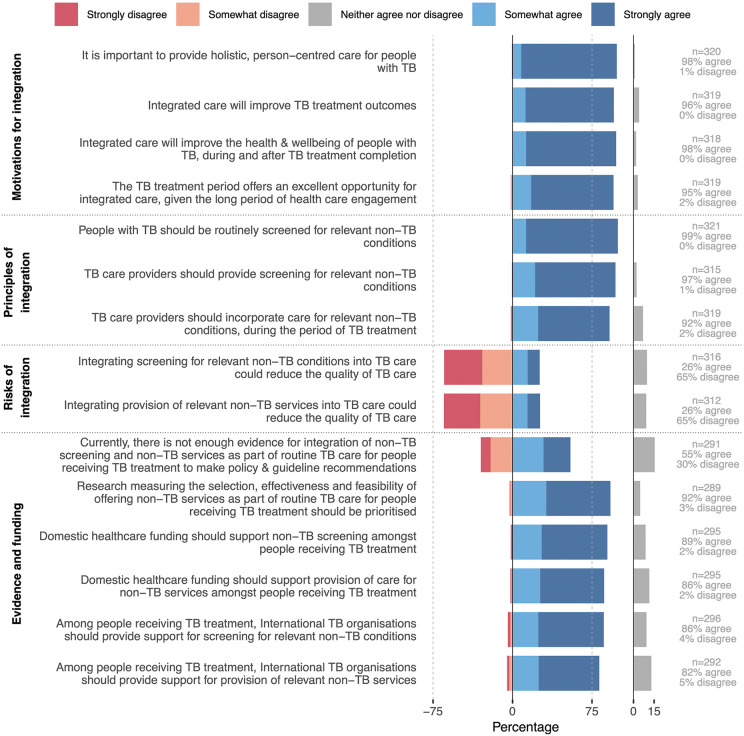
Agreement with statements outlining the motivation, principles, risks and needs for evidence generation and funding for integrated services for people with tuberculosis (N = 324). Data collected as checkboxes on a 5-point Likert scale. The y-axis represents the statements presented to panellists and the x-axis the cumulative proportion of panellists who somewhat agreed and strongly agreed with the statements (positive scale) or who somewhat disagreed and strongly disagreed (negative scale). Not all panellists provided a response for every statement: N is the total number of panellists and n (per statement) is the number of panellists providing a response to that statement. Responses that neither agreed nor disagreed with the statements are displayed separately (grey bars). Consensus was considered reached if ≥75% of panellists either agreed or disagreed (grey dotted line). Abbreviations: TB, tuberculosis.

In round 1, 98% of panellists agreed that they would consider screening people with tuberculosis for non-tuberculosis conditions or offering them non-tuberculosis health services; 84% agreed with this statement applied to household contacts. Critically, panellists recognised the close relationship between tuberculosis and other conditions as a motivation for integration (Table 9 in S1 Appendix). For most panellists the underlying goal of integrated services was to impact tuberculosis-specific outcomes (e.g., treatment adherence, treatment outcomes or post-tuberculosis morbidity, and tuberculosis incidence among household contacts). Other motivators were the “opportunity” to provide services to a particularly vulnerable segment of society and the broader ethical imperative of holistic, person-centred care. Seven panellists identified the potential for integration to attract and retain men by including desirable services (Table 10 in S1 Appendix). Draft consensus statements and priority conditions (Fig 2 & Table 11 in S1 Appendix) were developed from thematic analysis; this, and input from the study steering committees, also resulted in addition of three statements on population-wide integrated screening for round 2.

The following sections summarise consensus established from the round 2 survey. Findings were consistent across subgroups, unless otherwise specified.

### Motivation, principles and risks of integrated tuberculosis services

Consensus was reached on each of the proposed motivations for integrated care for people with tuberculosis (Fig 2; Table 12-15 in S1 Appendix): holistic, person-centred care (98%), an opportunity to intervene (95%), improved tuberculosis treatment outcomes (96%), and improved health and well-being of people with tuberculosis during and after tuberculosis treatment completion (98%). Consensus was reached that people with tuberculosis should be routinely screened for relevant non-tuberculosis conditions (99%); screening should be provided by tuberculosis care providers (97%); and care should be provided for relevant non-tuberculosis conditions by tuberculosis care providers during the period of tuberculosis treatment (92%; Table 16 in S1 Appendix). A quarter of panellists (26%) expressed concern that integration could reduce the quality of tuberculosis care. The most common concern (stated by 32/63 panellists who provided free-text comments; Table 17 in S1 Appendix) was feasibility if resources are inadequate. Fourteen panellists commented that effective integration would in fact improve the quality of tuberculosis care, including through improved tuberculosis outcomes, and, ultimately, decreased tuberculosis incidence. There was a lack of consensus on the current state of evidence on service integration among people with tuberculosis, with half of panellists agreeing and a third disagreeing that there is not enough evidence; 92% agreed that such research should be prioritised (Table 18 in S1 Appendix). Consensus was reached that both domestic (82% agreed) and international (89% agreed) funding should support the provision of screening and care for non-tuberculosis conditions among people with tuberculosis.

**Table 1:** Considerations for implementation of integrated tuberculosis screening and care.

Building block*	Theme
Service delivery	Screening aids, management guidelines, information, education and counselling materials, standard operating procedures, software and tools
	Free, accessible and timely treatment programs/referral services available for conditions that are screened
	Well-established, well-run community-based healthcare presence (strong primary care)
	Development of more accessible, affordable point-of-care tests for conditions
	Reliable supply chains
	Functioning, sufficiently resourced base patient follow-up and household contact tracing process
	Development of appropriate community-based psychosocial care programs
	Evidence of successful integration in similar setting
Health workforce	Staff training and mentorship
	Human resource/ availability of healthcare workers
	Healthcare worker buy-in and engagement with person-centred care
	Sufficient clinician time to develop plans for integrated care
Health information systems	Well-linked laboratory services
	Robust electronic medical record system
	Monitoring and evaluation systems
Essential diagnostics and medicines	Equipment/diagnostics/test-kits for screening that are appropriate for use in the community
Financing	Adequate financing
	Support for patients to attend additional medical appointments
Leadership and governance	Close collaboration among health departments, non-governmental organisations, community organisations and other stakeholders
	National policies in place
	Political commitment
	Strong leadership and governance
Community engagement	Strong community engagement, sensitization and buy-in

* Themes are mapped to the World Health Organisation health system building blocks.

**Fig 3:**
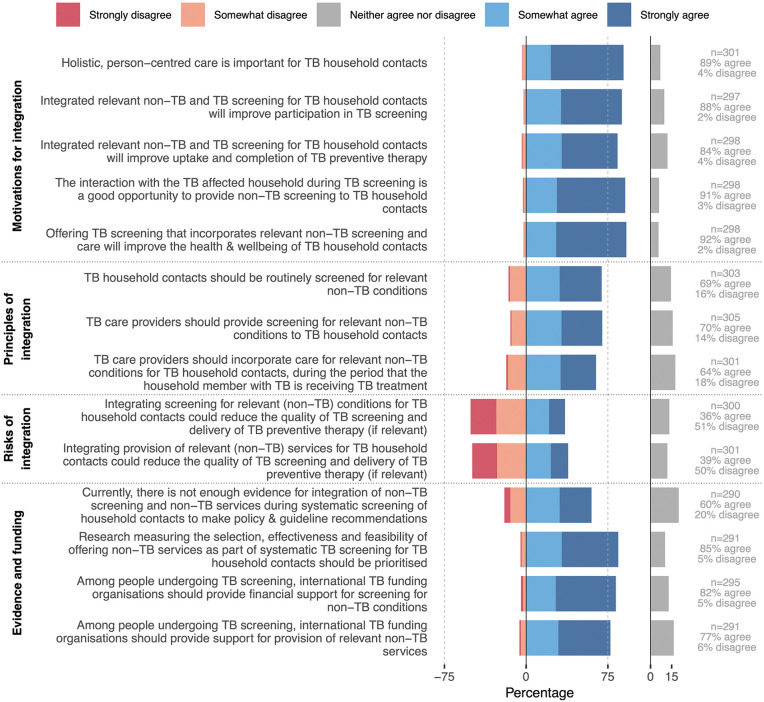
Agreement with statements outlining the motivation, principles, risks and needs for evidence generation and funding for integrated services for tuberculosis household contacts (N = 324). Data collected as checkboxes on a 5-point Likert scale. The y-axis lists the statements presented and the x-axis the cumulative proportion of panellists who somewhat agreed and strongly agreed (positive scale) or who somewhat disagreed and strongly disagreed (negative scale). Not all panellists provided a response for every statement: N is the total number of panellists and n (per statement) is the number providing a response to that statement. Consensus was considered reached when ≥75% of panellists either agreed or disagreed (grey dotted line). Abbreviations: TB, tuberculosis.

Similar to the results for people with tuberculosis, most panellists (89%) agreed that it is important to provide holistic, person-centred care for household contacts (Fig 3) and that integrated services will improve participation in tuberculosis screening (88%); uptake and completion of tuberculosis preventive therapy (TPT) (84%); and the overall health and wellbeing of household contacts (92%). Whilst 91% of participants agreed that tuberculosis screening offers a good opportunity to provide expanded screening, agreement was not reached on routine screening for relevant non-tuberculosis conditions among household contacts (69% agreed, 16% disagreed, 15% equivocal). More (≥36%) panellists expressed concern about the potential risks of integrated screening and care for non-tuberculosis conditions in this context compared to equivalent statements for people with tuberculosis. Themes related mostly to feasibility and resource constraints and included the potential for integrated screening for non-tuberculosis conditions to reduce quality of tuberculosis screening and TPT delivery (Table 17 & 19 in S1 Appendix). The majority (60%) of panellists agreed that current evidence is insufficient to make policy and guideline recommendations, with 85% agreeing that such research should be prioritised. Panellist opinions varied more across world regions and professional groups here than for the comparable statements related to people with tuberculosis, with the strongest support for integration from members of tuberculosis-affected community (Tables S20–21 in S1 Appendix). Consequently, consensus was stronger, and the consensus threshold reached for routine screening for non-tuberculosis conditions among household contacts, in sensitivity analyses assigning equal weight to each professional group (Table 22 in S1 Appendix).

Common themes on barriers and enablers for feasible and sustainable implementation of integrated tuberculosis care or screening services related to the need for service delivery support, strong primary care, referral services, and access to sufficient human and financial resources (Table 1 & Table 23 in S1 Appendix). The need for underpinning political buy-in and collaboration across health departments and other organisations was widely recognised.

Consensus was reached that population-wide systematic tuberculosis screening programmes offer a good opportunity to screen for other conditions (Fig 3 & Table 24–25 in S1 Appendix). Panellists agreed that people participating in population-wide tuberculosis screening should be offered screening for relevant non-tuberculosis conditions (93%) and be able to access relevant care (89%). Thematic analysis again highlighted how optimal combinations require careful context-specific consideration, including feasibility, local service availability and community member preferences (Table 26 in S1 Appendix). Nonetheless, panellists suggested that integrated population-wide tuberculosis screening could increase participation compared to tuberculosis screening alone, improve health literacy, and reduce tuberculosis-related stigma and discrimination.

### Priority conditions for and current status of integrated tuberculosis services

**Fig 4:**
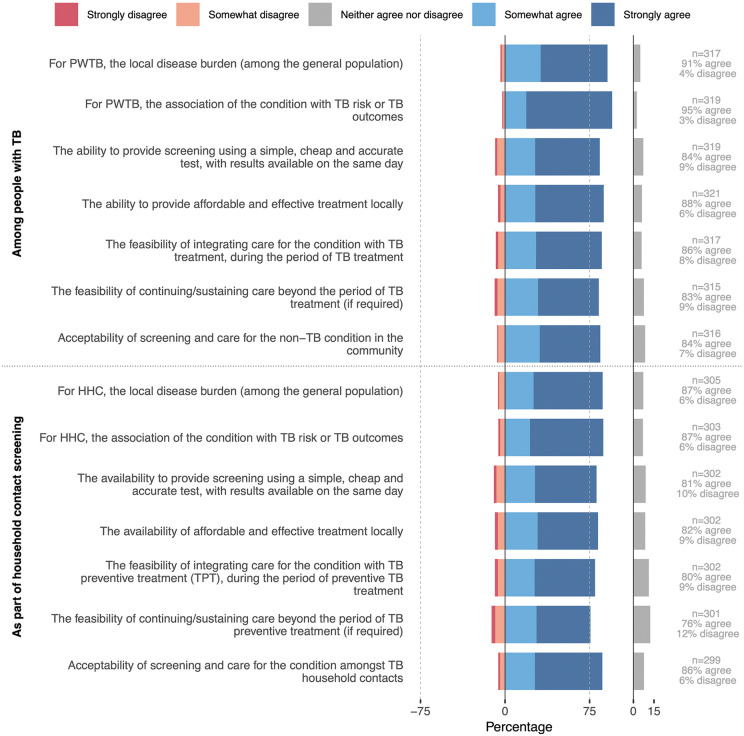
Agreement with statements outlining the key considerations for selection of conditions and services warranting integrated tuberculosis screening and care (N = 324). Data collected as checkboxes on a 5-point Likert scale. The y-axis represents the statements presented to panellists and the x-axis the cumulative proportion of panellists who somewhat agreed and strongly agreed with the statements (positive scale) or who somewhat disagreed and strongly disagreed (negative scale). Not all panellists provided a response for every statement: N is the total number of panellists and n (per statement) is the number of panellists providing a response to that statement. Responses that neither agreed nor disagreed with the statements are displayed separately (grey bars). Consensus was considered reached if ≥75% of panellists either agreed or disagreed (grey dotted line). Abbreviations: HHC, household contacts; PWTB, people with tuberculosis.

For both people with tuberculosis and household contacts, consensus was reached for all proposed criteria to define a non-tuberculosis condition as relevant for inclusion in an integrated service delivery programme (Fig 4; Table 27–28 in S1 Appendix). These included the 1) local disease burden, 2) association of the condition with tuberculosis risk or adverse tuberculosis outcomes, 3) ability to provide screening using a simple, cheap and accurate test, 4) availability of affordable and effective treatment locally, 5) feasibility of integrating care into that for tuberculosis, 6) feasibility of continuing care after tuberculosis treatment and 7) acceptability of screening and care within the community.

**Fig 5:**
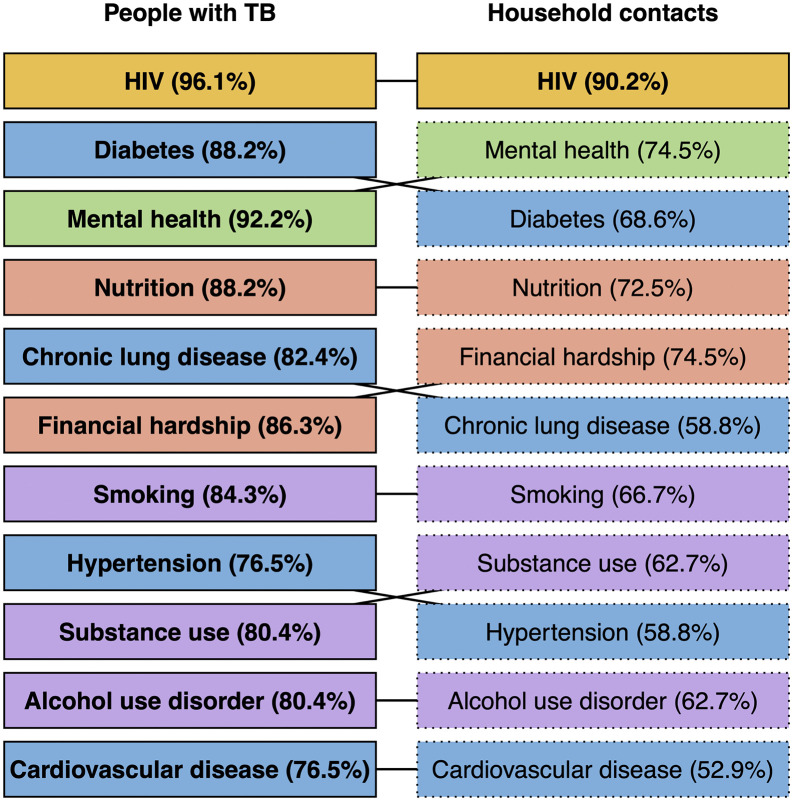
Ranking of the most important conditions and services to be included for people with tuberculosis and household contacts (n = 375 responses for 80 country settings; survey round 2). Conditions are ordered by their ranking across all country-specific scenarios provided. Percentages shown are the percentages of individual country settings in which panellists agreed this condition should be included in integrated screening or care. Solid borders and bold font indicate where the condition was ranked by panellists in ≥75% of country settings. Colours indicate the ‘category’ of the condition: yellow = infectious diseases, blue = non-communicable diseases, green = mental health and purple = substance use disorders.

For questions relating to the status of and priority conditions for integrated tuberculosis screening and care, 279 people (86%) named at least one country in which they were familiar with the routine tuberculosis programme, providing 375 responses for 80 countries. In 89% of countries, HIV testing was offered to people with tuberculosis (Table 29 in S1 Appendix). All other services were reported as being available for people with tuberculosis in fewer than half of settings, with nutritional support (31%) and diabetes screening (29%) being most common. Of note, nutritional support was often only for people with drug-resistant tuberculosis. HIV testing was offered to household contacts in 25% of country settings; very few other services were so. When panellists were asked to rank conditions or services most important for integration, HIV ranked highest (suggested in 96% of country settings for people with TB and 90% for household contacts; Fig 5 & Table 30 in S1 Appendix). Next were diabetes (suggested in 88% and 69% of settings), followed by mental health (92% and 75%) and nutrition (88% and 73%). The order in which conditions were ranked differed across world regions and professional groups (Fig 5-8 in S1 Appendix). For people with TB, all suggested conditions reached the consensus cut-off (>75%); among household contacts, only HIV did so (Fig 5).

## Discussion

This two-round Delphi process established broad consensus on the motivations and principles of integrated tuberculosis services (Table 31 in S1 Appendix). Importantly, we demonstrated that the global tuberculosis community does not view integrated care as ‘beyond the scope’ of tuberculosis programmes; rather, experts strongly endorsed holistic care of tuberculosis-affected communities and identified the period of tuberculosis treatment or screening as an opportunity to intervene and improve the health of vulnerable communities. People with tuberculosis should be routinely screened and offered care for comorbidities as part of their treatment, with consequent benefits including improved adherence to treatment and outcomes. Among household contacts, we did not establish consensus that other screening or services should be routinely integrated. Notably, the strongest support for integrated services for household contacts was from affected community, suggesting a resonance with their lived experience divergent from that of other professional groups.

In contrast to the consensus on motivation for and principles of integrated tuberculosis services, panellists opinions on the potential risks of this approach and the current state of evidence were mixed. Panellists however agreed that research to guide service development and implementation should be prioritised, and that funders need to allow for provision of non-tuberculosis specific services within their programmes. Most concerns related to resource limitations, feasibility and a perceived lack of existing evidence.

There is clear evidence of the high prevalence of comorbidities among people with tuberculosis and their role in driving adverse outcomes [2,20], with many calls for integration of care by experts [21–23] and WHO documents [5]. The conditions previously identified as important for tuberculosis risk and outcomes were those most highly ranked here [3]. Tuberculosis-HIV integration provides an important example of the power of cross-programme collaboration, facilitated by dedicated, disease-specific funding mechanisms. However, evidence for the integration of other conditions and services, without strong vertical funding and programming, remains limited [24]. Studies suggest household contacts face an increased risk of chronic conditions and are crucial treatment supporters, yet barriers to healthcare including tuberculosis screening are substantial [25,26]. Addressing stigma, providing incentives and psychosocial support, and fostering trust between communities and providers can enhance screening participation [27,28]. In household contact, and more so population-wide, screening for tuberculosis, the diagnostic yield is low (≤1% [13]). Here, integration may deliver economy of scope, making it logistically and financially possible to deliver necessary services. Emerging evidence suggests that integrated screening is feasible, can result in a high yield of new diagnoses at an earlier stage and can be cost-effective; though gaps in linkage-to-care remain [29,30]. The consensus established here endorses and informs future research into integrated screening for tuberculosis-affected households and in population-wide screening settings. Maximising benefits from integration must consider health system, funder and community perspectives. Gender disparities in tuberculosis risk factors (e.g., smoking, alcohol use and silica dust exposure among men; HIV among women) and screening uptake (often lower among men) warrant targeted strategies to enhance desirability of tuberculosis screening and care across genders.

The substantial health system barriers to integration identified in this study align with earlier pilot studies [31]. While safeguarding core tuberculosis programme activities is crucial, progress towards global tuberculosis targets remains off-track. New approaches, which might include integrated screening and care, are needed to ensure i) that people with tuberculosis come forward for care, are supported to take treatment by healthcare providers and their social networks, and achieve health outcomes that are desirable for them; and ii) that household contacts are well equipped to support people with tuberculosis, are willing to participate in tuberculosis screening, and start and complete TPT [28,32]. Healthcare workers delivering tuberculosis services in high-tuberculosis incidence settings face workforce and commodity shortages whilst caring for a complex population with multiple medical and social needs–they cannot be expected to replace robust primary care [33]. In this study panellists highlighted the need to integrate both activities and programming including strengthening cross-programme collaboration at the governmental level such that delivery of integrated screening and care does not become wholly the responsibility of tuberculosis programmes and their staff. Whilst this should form part of national financing, either through primary care budgets or public and private health insurance mechanisms, disease-specific funding agencies who promote integration should also allow money to be used for screening and care of related conditions, or facilitate resource mobilization. At the programme and policy level, aligning efforts for integrated tuberculosis services within broader health systems strengthening–including development of primary care services and universal health coverage–may enhance individual care experiences, reduce TB stigma, and reveal additional sources of financing. Further research is needed to strike a successful balance between tuberculosis programmes meeting needs of tuberculosis-affected households and driving down tuberculosis vulnerability, whilst avoiding overburdening the healthcare workforce.

It should be noted that integration of tuberculosis screening and care is already happening in different contexts, as reported by our panellists. However this currently lacks coordination and robust underpinning evidence [8]. This study provides a clear mandate for further evidence generation as a prerequisite for concrete recommendations and widescale adoption. The priority conditions identified and consensus on the principles by which conditions and services should be selected for possible integration provide a foundation for developing and evaluating interventions. Whilst many priority conditions are identifiable and treatable with low-cost, decentralizable tools, tailored interventions will be needed for the tuberculosis context. Importantly, whilst mental health is a key priority [34,35], models of effective psychosocial support for people affected by tuberculosis are lacking in most settings.

Strengths of our study include an innovative Delphi approach, including researchers with lived experience of tuberculosis. We used a broad definition of expertise, engaged a wide range of networks, and provided the survey in multiple languages. Unlike most Delphi studies, we used open-ended questions in round 1, allowing for diverse perspectives and richer data. We monitored participant demographics in real time, reaching out to underrepresented groups. Two expert steering committees guided consensus development. To enhance inclusivity in round 2, we added a Vietnamese translation, expanded outreach to tuberculosis survivor networks, and created an accessible video summary. Whilst some groups were over-represented and others underrepresented in our panellists, subgroup stratification suggested this had little impact on consensus findings.

Limitations of the study include potential sampling bias, as panellists opting to complete the surveys were likely predisposed to support integrated tuberculosis services. Our records of contacted individuals were incomplete (e.g., we did not have the number of mailing list subscribers), preventing precise assessment of response rates. Secondly, proportional representation of tuberculosis survivors and civil-society organisations is low; we did however include a greater absolute number of people representing affected community than is usual in consensus exercises. Delphi methods may be less accessible for non-research or non-clinical stakeholders; integrating in-depth interviews or focus groups could better capture survivor perspectives in expert consensus development. Finally, we did not explore the needs of specific populations such as children and adolescents, or people with extrapulmonary tuberculosis.

In conclusion, this Delphi survey demonstrated broad consensus on the principles and motivation for integrated tuberculosis screening and care and identified priority conditions to guide development of integrated services. However further research is needed to provide robust evidence of feasibility, effectiveness and scale-up potential in support of wide-scale implementation.

## Supporting information

S1 Appendix Supplementary materials. (DOCX)

S1 Checklist Human subjects research checklist. (PDF)

## References

[1] World Health Organization. Global Tuberculosis Report 2025. Geneva: World Health Organization. 2025.

[2] Marais BJ, Lönnroth K, Lawn SD, Migliori GB, Mwaba P, Glaziou P, et al. Tuberculosis comorbidity with communicable and non-communicable diseases: integrating health services and control efforts. Lancet Infect Dis. 2013;13(5):436–48. doi: 10.1016/S1473-3099(13)70015-X 23531392

[3] World Health Organization. Global Tuberculosis Report 2024. Geneva: World Health Organization. 2024.

[4] World Health Organization. Guideline: nutritional care and support for patients with tuberculosis. Geneva: World Health Organization. 2013. https://apps.who.int/iris/handle/10665/94836 24624480

[5] World Health Organization. Framework for collaborative action on tuberculosis and comorbidities. Geneva: World Health Organization. 2022.

[6] World Health Organization. Operational handbook on tuberculosis: module 6: tuberculosis and comorbidities. 3rd ed. Geneva: World Health Organization. 2024.

[7] Jarde A, Siqueira N, Afaq S, Naz F, Irfan M, Tufail P, et al. Addressing TB multimorbidity in policy and practice: An exploratory survey of TB providers in 27 high-TB burden countries. PLOS Glob Public Health. 2022;2(12):e0001205. doi: 10.1371/journal.pgph.0001205 36962813 PMC10022227

[8] Foo CD, Shrestha P, Wang L, Du Q, García-Basteiro AL, Abdullah AS, et al. Integrating tuberculosis and noncommunicable diseases care in low- and middle-income countries (LMICs): A systematic review. PLoS Med. 2022;19(1):e1003899. doi: 10.1371/journal.pmed.1003899 35041654 PMC8806070

[9] Hamada Y, Quartagno M, Law I, Malik F, Bonsu FA, Adetifa IMO, et al. Tobacco smoking clusters in households affected by tuberculosis in an individual participant data meta-analysis of national tuberculosis prevalence surveys: Time for household-wide interventions?. PLOS Glob Public Health. 2024;4(2):e0002596. doi: 10.1371/journal.pgph.0002596 38422092 PMC10903843

[10] Zayar NN, Chotipanvithayakul R, Bjertness E, Htet AS, Geater AF, Chongsuvivatwong V. Vulnerability of NCDs and Mediating Effect of Risk Behaviors Among Tuberculosis Patients and Their Household Contacts Compared to the General Population in the Yangon Region, Myanmar. Int J Gen Med. 2023;16:5909–20. doi: 10.2147/IJGM.S439141 38106977 PMC10725691

[11] Hamada Y, Quartagno M, Malik F, Ntshamane K, Tisler A, Gaikwad S, et al. Prevalence of non-communicable diseases among household contacts of people with tuberculosis: A systematic review and individual participant data meta-analysis. Trop Med Int Health. 2024;29(9):768–80. doi: 10.1111/tmi.14038 39073229 PMC11368628

[12] Galea JT, Chu AL, Sweetland AC, Jimenez J, Yataco R, Calderón R, et al. Latent TB and depressive symptoms in household contacts of persons with active TB. Int J Tuberc Lung Dis. 2023;27(9):682–7. doi: 10.5588/ijtld.22.0609 37608477 PMC10443790

[13] World Health Organization. WHO consolidated guidelines on tuberculosis. Module 2: screening – systematic screening for tuberculosis disease. Geneva: World Health Organization. 2021. 33822560

[14] Calderwood CJ, Timire C, Mavodza C, Kavenga F, Ngwenya M, Madziva K, et al. Beyond tuberculosis: a person-centred and rights-based approach to screening for household contacts. Lancet Glob Health. 2024;12(3):e509–15. doi: 10.1016/S2214-109X(23)00544-2 38365421

[15] Zayar N-N, Sangthong R, Saw S, Aung ST, Chongsuvivatwong V. Combined Tuberculosis and Diabetes Mellitus Screening and Assessment of Glycaemic Control among Household Contacts of Tuberculosis Patients in Yangon, Myanmar. Trop Med Infect Dis. 2020;5(3):107. doi: 10.3390/tropicalmed5030107 32610514 PMC7558353

[16] Hamada Y, Lugendo A, Ntshiqa T, Kubeka G, Lalashowi JM, Mwastaula S, et al. A pilot cross-sectional study of non-communicable diseases in TB household contacts. IJTLD Open. 2024;1(4):154–9. doi: 10.5588/ijtldopen.23.0579 38988408 PMC11231826

[17] Scott P, Elsayedkarar M, Marambire E, Ncube G, Apollo T, Kavenga F, et al. HIV testing during systematic screening for tuberculosis among household contacts in high-tuberculosis burden settings: a systematic review and meta-analysis. Lancet Glob Health. 2026;14(1):e81–91. doi: 10.1016/S2214-109X(25)00437-1 41386250

[18] Gattrell WT, Logullo P, van Zuuren EJ, Price A, Hughes EL, Blazey P, et al. ACCORD (ACcurate COnsensus Reporting Document): A reporting guideline for consensus methods in biomedicine developed via a modified Delphi. PLoS Med. 2024;21(1):e1004326. doi: 10.1371/journal.pmed.1004326 38261576 PMC10805282

[19] Diamond IR, Grant RC, Feldman BM, Pencharz PB, Ling SC, Moore AM, et al. Defining consensus: a systematic review recommends methodologic criteria for reporting of Delphi studies. J Clin Epidemiol. 2014;67(4):401–9. doi: 10.1016/j.jclinepi.2013.12.002 24581294

[20] Janse Van Rensburg A, Dube A, Curran R, Ambaw F, Murdoch J, Bachmann M, et al. Comorbidities between tuberculosis and common mental disorders: a scoping review of epidemiological patterns and person-centred care interventions from low-to-middle income and BRICS countries. Infect Dis Poverty. 2020;9(1):4. doi: 10.1186/s40249-019-0619-4 31941551 PMC6964032

[21] Lönnroth K, Castro KG, Chakaya JM, et al. Tuberculosis control and elimination 2010–50: cure, care, and social development. The Lancet. 2010;375:1814–29. 10.1016/S0140-6736(10)60483-7 20488524

[22] Marais BJ, Lönnroth K, Lawn SD, Migliori GB, Mwaba P, Glaziou P, et al. Tuberculosis comorbidity with communicable and non-communicable diseases: integrating health services and control efforts. Lancet Infect Dis. 2013;13(5):436–48. doi: 10.1016/S1473-3099(13)70015-X 23531392

[23] Wingfield T, Tovar MA, Huff D, Boccia D, Saunders MJ, Datta S, et al. Beyond pills and tests: addressing the social determinants of tuberculosis. Clin Med (Lond). 2016;16(Suppl 6):s79–91. doi: 10.7861/clinmedicine.16-6-s79 27956446 PMC6329567

[24] Hyle EP, Naidoo K, Su AE, El-Sadr WM, Freedberg KA. HIV, tuberculosis, and non-communicable diseases: what is known about the costs, effects, and cost-effectiveness of integrated care?. Journal of Acquired Immune Deficiency Syndromes. 2014;67:S87. 10.1097/QAI.0000000000000254 PMC4147396 25117965

[25] Taylor M, Medley N, van Wyk SS, Oliver S. Community views on active case finding for tuberculosis in low- and middle-income countries: a qualitative evidence synthesis. Cochrane Database Syst Rev. 2024;3(3):CD014756. doi: 10.1002/14651858.CD014756.pub2 38511668 PMC10955804

[26] Calderwood CJ, Marambire ET, Ngwerume M, Tshuma M, Coleman M, Musunzuru T, et al. Integrated health checks as a person-centred approach to systematic screening of household tuberculosis contacts: A realist-informed mixed-methods study. PLOS Glob Public Health. 2025;5(11):e0005146. doi: 10.1371/journal.pgph.0005146 41183019 PMC12582465

[27] Matteelli A, Churchyard G, Cirillo D, den Boon S, Falzon D, Hamada Y, et al. Optimizing the cascade of prevention to protect people from tuberculosis: A potential game changer for reducing global tuberculosis incidence. PLOS Glob Public Health. 2024;4(7):e0003306. doi: 10.1371/journal.pgph.0003306 38954723 PMC11218967

[28] Barss L, Moayedi-Nia S, Campbell JR, Oxlade O, Menzies D. Interventions to reduce losses in the cascade of care for latent tuberculosis: a systematic review and meta-analysis. Int J Tuberc Lung Dis. 2020;24(1):100–9. doi: 10.5588/ijtld.19.0185 32005312

[29] Chamie G, Kwarisiima D, Clark TD, Kabami J, Jain V, Geng E, et al. Leveraging rapid community-based HIV testing campaigns for non-communicable diseases in rural Uganda. PLoS One. 2012;7(8):e43400. doi: 10.1371/journal.pone.0043400 22916256 PMC3423366

[30] Govindasamy D, Kranzer K, van Schaik N, Noubary F, Wood R, Walensky RP, et al. Linkage to HIV, TB and non-communicable disease care from a mobile testing unit in Cape Town, South Africa. PLoS One. 2013;8(11):e80017. doi: 10.1371/journal.pone.0080017 24236170 PMC3827432

[31] Nunemo MH, Gidebo KD, Woticha EW, Lemu YK. Integration Challenges and Opportunity of Implementing Non-Communicable Disease Screening Intervention with Tuberculosis Patient Care: A Mixed Implementation Study. Risk Manag Healthc Policy. 2023;16:2609–33. doi: 10.2147/RMHP.S432943 38045564 PMC10693204

[32] Alsdurf H, Hill PC, Matteelli A, Getahun H, Menzies D. The cascade of care in diagnosis and treatment of latent tuberculosis infection: a systematic review and meta-analysis. Lancet Infect Dis. 2016;16(11):1269–78. doi: 10.1016/S1473-3099(16)30216-X 27522233

[33] Saunders MJ, Tovar MA, Datta S, Evans BEW, Wingfield T, Evans CA. Pragmatic tuberculosis prevention policies for primary care in low- and middle-income countries. Eur Respir J. 2018;51(3):1800315. doi: 10.1183/13993003.00315-2018 29567728

[34] World Health Organization. WHO operational handbook on tuberculosis Module 6: Tuberculosis and comorbidities Mental health conditions. Geneva: World Health Organization. 2023.

[35] Patwal R, Sachdeva A, Bhaskarapillai B, Arasappa R, Muliyala KP, Desai G. Prevalence of suicidal ideations and suicide attempts in patients with tuberculosis: A systematic review and meta-analysis. J Psychosom Res. 2023;167:111171. doi: 10.1016/j.jpsychores.2023.111171 36753943

