# Supplementary material for: Principles and priorities for integrated tuberculosis screening and care: A modified Delphi consensus exercise
Source: PLOS Glob Public Health. 2026 Mar 2;6(3):e0005954. doi: 10.1371/journal.pgph.0005954 (PMC12952631; doi:10.1371/journal.pgph.0005954)
Supplement: S1 Appendix — (DOCX) [file pgph.0005954.s001.docx]

Global tuberculosis expert consensus on principles and priorities for integrated tuberculosis screening and care: a modified Delphi exercise

SUPPLEMENTARY MATERIALS

**Table of contents**

[Supplementary methods 3](#_Toc211266771)

[Role of the researchers 3](#_Toc211266772)

[Table S1: ACcurate COnsensus Reporting Document (ACCORD) checklist 3](#_Toc211266773)

[Table S2: Glossary of terms 4](#_Toc211266774)

[Literature review 4](#_Toc211266775)

[Table S3: Search strategies used to identify relevant literature on tuberculosis comorbidities 4](#_Toc211266776)

[Table S4: Association between chronic conditions and tuberculosis 6](#_Toc211266777)

[Table S5: A timeline of United Nations / World Health Organisation declarations, policies and guidelines of relevance to integrated tuberculosis screening and care 10](#_Toc211266778)

[Table S6: Strategies used to identify relevant literature on chronic conditions among members of TB-affected households 11](#_Toc211266779)

[Table S7: Key Findings on Non-Communicable Diseases, Mental Health, and Nutrition among TB Household Contacts 11](#_Toc211266780)

[Delphi survey design and distribution 11](#_Toc211266781)

[Data analysis 12](#_Toc211266782)

[Refinement of the approach in response to round 1 findings 13](#_Toc211266783)

[Figure S1: Flowchart summarising Delphi survey development and conduct 14](#_Toc211266784)

[Supplementary results 15](#_Toc211266785)

[Table S8: Delphi panellist demographic details 15](#_Toc211266786)

[Round 1 consensus: dissenting voices 15](#_Toc211266787)

[Development of consensus statements 16](#_Toc211266788)

[Table S9: Rationale for offering non-tuberculosis screening or care services to people with tuberculosis and household contacts (survey round 1) 17](#_Toc211266789)

[Table S10: Gender considerations in development of integrated tuberculosis screening and care (survey round 1) 18](#_Toc211266790)

[Figure S2: Non-tuberculosis conditions for which experts would offer screening and care among people with tuberculosis and their household contacts, ranked by frequency of votes and reported as both total expert responses and by subgroup of tuberculosis survivor responses (survey round 1) 20](#_Toc211266791)

[Table S11: Conditions mentioned by panellists as not being suitable for inclusion in integrated tuberculosis screening or care services, stratified by whether mentioned with regard to people with tuberculosis or household contacts (survey round 1) 21](#_Toc211266792)

[Table S12: Themes relating to the motivations (and concerns) for integrated TB screening and care, from free text comments (survey round 2) 21](#_Toc211266793)

[Table S13: Agreement with statements outlining the motivation, principles, risks and needs for evidence generation and funding of integrated services for people with tuberculosis, stratified by WHO region and professional group of the panellist (N=324) 23](#_Toc211266794)

[Table S14: Percentage of panellists selecting an ambivalent response for statements about the motivation, principles, risks and needs for evidence generation and funding of integrated care for people with tuberculosis, stratified by WHO region and professional group of the panellist (N=324) 24](#_Toc211266795)

[Table S15: Agreement with statements outlining the motivations, principles, risks and need for evidence generation and funding of integrated services for people with tuberculosis, giving equal weighting to respondents from each WHO region, country or professional group (N=324) 25](#_Toc211266796)

[Table S16: Themes relating to the principles of integrated tuberculosis screening and care, from free text comments (survey round 2) 25](#_Toc211266797)

[Table S17: Themes relating to the risks of integration for quality of tuberculosis screening and care, stratified by whether the panellist agreed that such risks existed (survey round 2) 27](#_Toc211266798)

[Table S18: Themes on evidence generation and funding for integrated screening and care (both people with tuberculosis and household contacts; round 2 survey) 27](#_Toc211266799)

[Table S19: Illustrative quotes from comments provided for statement “Household contacts of people treated for tuberculosis should be routinely screened for relevant non-tuberculosis conditions”, among people who disagreed with this statement (survey round 2; n=21/48 provided comments) 28](#_Toc211266800)

[Table S20: Agreement with statements outlining the motivations, principles, risks and need for evidence generation and funding of integrated services for tuberculosis household contacts, stratified by WHO region and professional group of the panellist (N=324) 29](#_Toc211266801)

[Table S21: Percentage of panellists selecting an ambivalent response for statements about motivation, principles, risks and needs for evidence generation and funding for integrated services for tuberculosis household contacts, stratified by WHO region and professional group of the panellist (N=324) 30](#_Toc211266802)

[Table S22: Agreement with statements outlining the motivations, principles, risks and need for evidence generation and funding of integrated services for tuberculosis household contacts, giving equal weighting to respondents from each WHO region, country or professional group (N=324) 32](#_Toc211266803)

[Table S23: Considerations for implementation of integrated tuberculosis screening and care, from thematic analysis of free text responses (round 1) 33](#_Toc211266804)

[Figure S3: Agreement with statements outlining the motivations, principles, risks and need for evidence generation and funding of integrated services as part of population-wide tuberculosis active case finding activities (N=324) 34](#_Toc211266805)

[Table S24: Agreement with statements outlining the motivations and principles of integrated population-wide tuberculosis active case finding, stratified by WHO region or professional group (N=324) 35](#_Toc211266806)

[Table S25: Agreement with statements outlining the motivations and principles of integrated population-wide tuberculosis active case finding, giving equal weighting to respondents from each WHO region, country or professional group (N=324) 35](#_Toc211266807)

[Table S26: Themes on integrated population-wide screening interventions, by panellist agreement that screening for non-tuberculosis conditions should be routinely included in such programmes (round 2 survey; n=49 people left comments) 36](#_Toc211266808)

[Table S27: Agreement with statements outlining the key considerations for selection of conditions and services for integration into tuberculosis screening or care, stratified by WHO region or professional group (N=324) 37](#_Toc211266809)

[Table S28: Agreement with statements outlining the key considerations for selection of conditions and services for integration into tuberculosis screening or care, giving equal weighting to respondents from each WHO region, country or professional group (N=324) 38](#_Toc211266810)

[Current status of integrated tuberculosis screening and care 38](#_Toc211266811)

[Table S29: Services reported by participants as already being available in their setting (survey round 2) 38](#_Toc211266812)

[Figure S4: Ranking of the most important conditions and services to be included for people with tuberculosis and household contacts (n=375 responses for 80 country settings) 39](#_Toc211266813)

[Table S30: Number of times conditions to be included as part of integrated care for people with TB and household contacts were ranked in first, second and third place, and total number of rankings received (n=375 rankings for 80 countries; survey round 2) 39](#_Toc211266814)

[Figure S5: Ranking of conditions stratified by WHO world region (N=375 individual country scenarios) 40](#_Toc211266815)

[Figure S6: Ranking of conditions using weighted average ranking across strata of WHO world region (N=375 individual country scenarios) 41](#_Toc211266816)

[Figure S7: Ranking of conditions stratified by professional group (N=375 individual country scenarios) 42](#_Toc211266817)

[Figure S8: Ranking of conditions using weighted average ranking across strata of professional group (N=375 individual country scenarios) 43](#_Toc211266818)

[Table S31: Core list of consensus statements on integrated screening and care for people with tuberculosis and their household contacts 43](#_Toc211266819)

# Supplementary methods

## Role of the researchers

Those directing the consensus exercise were tuberculosis researchers with mixed qualitative and quantitative expertise and lived experience. Claire Calderwood and Tenzin Kunor conceived of the study, collaboratively developed the protocol and designed survey round 1, based on a literature review and drawing on guidance from Eneyi Kpokori, a Delphi method expert and Katharina Kranzer. Whilst our questions were informed by literature review, we did not present a summary of the literature to panellists. Claire Calderwood, Tenzin Kunor and Mikaela Coleman analysed the first-round survey. Survey round 2 was designed by Claire Calderwood, Tenzin Kunor, Mikaela Coleman and Katharina Kranzer with input from two advisory steering committees comprised of tuberculosis researchers and tuberculosis survivors, many of whom also lived with tuberculosis co-morbidities. Steering committee members were Tom Wingfield, Madhavi Bhargava, Edson Marambire, Ben Marais, Sarah Bernays, Uzma Khan, Rosa Herrera, Jeffry Acaba, Zafar Hussain and Diptendu Bhattacharya. Distribution and analysis of the second-round survey was performed by Claire Calderwood and Mikaela Coleman. Survey panellists received no reimbursement for their time. All researchers and members of the two steering committees were responsible for distributing the survey to potential panellists. The core research team and members of the two steering committees were able to participate and contribute to consensus development as members of the panel.

Table 1: ACcurate COnsensus Reporting Document (ACCORD) checklist

| **Item No.** | **Section** | **Checklist Item (help text)** | **Page No.** |
| --- | --- | --- | --- |
| T1 | Title | Identify the article as reporting a consensus exercise and state the consensus methods used in the title. | 1 |
| I1 | Introduction | Explain why a consensus exercise was chosen over other approaches. | 5 & S1 |
| I2 |  | State the aim of the consensus exercise, including its intended audience and geographical scope (national, regional, global). | 5 & S1 |
| I3 |  | If the consensus exercise is an update of an existing document, state why an update is needed, and provide the citation for the original document. | NA |
| M1 | Methods  Registration | If the study or study protocol was prospectively registered, state the registration platform and provide a link. If the exercise was not registered, this should be stated. | S1 |
| M2 | Selection of SC and/or panellists | Describe the role(s) and areas of expertise or experience of those directing the consensus exercise. | S1 |
| M3 |  | Explain the criteria for panellist inclusion and the rationale for panellist numbers. State who was responsible for panellist selection. | S1 |
| M4 |  | Describe the recruitment process (how panellists were invited to participate). | 5 & S1 |
| M5 |  | Describe the role of any members of the public, patients or carers in the different steps of the study. | S1 |
| M6 | Preparatory research | Describe how information was obtained prior to generating items or other materials used during the consensus exercise. | 5 & S1 |
| M7 |  | Describe any systematic literature search in detail, including the search strategy and dates of search or the citation if published already. | S1 |
| M8 |  | Describe how any existing scientific evidence was summarised and if this evidence was provided to the panellists. | S1 |
| M9 | Assessing consensus | Describe the methods used and steps taken to gather panellist input and reach consensus (for example, Delphi, RAND-UCLA, nominal group technique). | 5 & S1 |
| M10 |  | Describe how each question or statement was presented and the response options. State whether panellists were able to or required to explain their responses, and whether they could propose new items. | Supp materials |
| M11 |  | State the objective of each consensus step. | 5 |
| M12 |  | State the definition of consensus (for example, number, percentage, or categorical rating, such as ‘agree’ or ‘strongly agree’) and explain the rationale for that definition. | 6 |
| M13 |  | State whether items that met the prespecified definition of consensus were included in any subsequent voting rounds. | NA |
| M14 |  | For each step, describe how responses were collected, and whether responses were collected in a group setting or individually. | 5 |
| M15 |  | Describe how responses were processed and/or synthesised.  Include qualitative analyses of free-text responses (for example, thematic, content or cluster analysis) and/or quantitative analytical methods, if used. | 6 |
| M16 |  | Describe any piloting of the study materials and/or survey instruments. | S1 |
| M17 |  | If applicable, describe how feedback was provided to panellists at the end of each consensus step or meeting. | 5 |
| M18 |  | State whether anonymity was planned in the study design. Explain where and to whom it was applied and what methods were used to guarantee anonymity. | 5 |
| M19 |  | State if the steering committee was involved in the decisions made by the consensus panel. | S1 |
| M20 | Participation | Describe any incentives used to encourage responses or participation in the consensus process. | 5 & S1 |
| M21 |  | Describe any adaptations to make the surveys/meetings more accessible. | S2 |
| R1 | Results | State when the consensus exercise was conducted. List the date of initiation and the time taken to complete each consensus step, analysis, and any extensions or delays in the analysis. | 5 |
| R2 |  | Explain any deviations from the study protocol, and why these were necessary.  For example, addition of panel members during the exercise, number of consensus steps, stopping criteria; report the step(s) in which this occurred. | NA |
| R3 |  | For each step, report quantitative (number of panellists, response rate) and qualitative (relevant socio-demographics) data to describe the participating panellists. | 6 &  S Table 2 |
| R4 |  | Report the final outcome of the consensus process as qualitative (for example, aggregated themes from comments) and/or quantitative (for example, summary statistics, score means, medians and/or ranges) data. | 7-9 |
| R5 |  | List any items or topics that were modified or removed during the consensus process. Include why and when in the process they were modified or removed. | NA |
| D1 | Discussion | Discuss the methodological strengths and limitations of the consensus exercise.  Include factors that may have impacted the decisions (for example, response rates, representativeness of the panel, potential for feedback during consensus to bias responses, potential impact of any non-anonymised interactions). | 11-12 |
| D2 |  | Discuss whether the recommendations are consistent with any pre-existing literature and, if not, propose reasons why this process may have arrived at alternative conclusions. | 13 |
| O1 | Other information | List any endorsing organisations involved and their role. | NA |
| O2 |  | State any potential conflicts of interests, including among those directing the consensus study and panellists. Describe how conflicts of interest were managed. | S1 |
| O3 |  | State any funding received and the role of the funder. | 6 |

**Abbreviations:** S = Supplementary materials.

Table 2: Glossary of terms

| **Term** | **Definition (provided to panellists)** |
| --- | --- |
| TB programme | Whoever delivers TB services. This includes National TB Programmes (NTPs), private TB care providers, TB NGOs etc and is dependent on context |
| Holistic care | About caring for the whole person — providing for physical, mental, spiritual, and social needs |
| Referral services | Other non-TB health or social care programmes (e.g. non-communicable disease services, social welfare or disability programmes) which are open to the general population. People with TB or household contacts may be referred to these services to address a specific non-TB issue e.g. diabetes, alcohol dependency etc. Often, these services are not provided in the same place that TB care is delivered |
| Non-TB condition | Any disease, syndrome, status or determinant that impacts health, other than TB (e.g. smoking, diabetes, HIV, financial hardship, mental health, hepatitis, nutrition). |
| Non-TB screening | The use of tests, diagnostics and assessments to determine whether a non-TB condition exists e.g. smoking questionnaire, HIV test, psychosocial assessment, blood test, measurement of height and weight, etc. |

## Literature review

Table 3: Search strategies used to identify relevant literature on tuberculosis comorbidities

| **Topic*** | **Search details^†^** |
| --- | --- |
| **Multimorbidity^‡^** | Pubmed search for multimorbidity OR “multiple long term conditions” AND tuberculosis in Title/Abstract on 18/07/2024 identified 67 articles (two systematic reviews^§^) of which 15 were considered relevant. |
| ***Communicable diseases*** | |
| **HIV** | Pubmed search for HIV AND tuberculosis AND “systematic review” in Title/Abstract on 14/02/2024 returned 547 results. |
| **Hepatitis B&C** | Pubmed search for (“hepatitis B” OR “hepatitis C”) AND tuberculosis AND “systematic review” in Title/Abstract on 14/02/2024 returned 49 results. |
| ***Risk behaviours*** | |
| **Alcohol use disorders** | Pubmed search for alcohol AND tuberculosis AND “systematic review” in Title/Abstract on 14/12/2023 identified two systematic reviews (2017 and 2018). |
| **Drug use disorders** | Pubmed search for (“drug use?” OR “substance use?” OR “people who use drugs”) AND tuberculosis AND “systematic review” in Title/Abstract on 03/03/2024 returned 24 results. |
| **Smoking** | Pubmed search for smoking and tuberculosis and “systematic review” in Title/Abstract on 03/03/2024. No language or date restrictions. 61 results returned |
| ***Non-communicable diseases*** | |
| **Chronic lung disease** | Pubmed search for COPD, chronic lung disease, chronic respiratory disease, lung function, pulmonary function, spirometry, asthma, chronic obstructive pulmonary disease, chronic pulmonary disease (all with ‘or’) and tuberculosis, and household in Title/Abstract on 03/03/2024 returned 64 results. |
| **Diabetes** | Pubmed search for diabetes and tuberculosis and “systematic review” in Title/Abstract on 14/12/2023. No language or date restrictions. 99 results returned. |
| **Cardiovascular disease** | Pubmed search for (“heart disease” OR “cardiovascular”) AND tuberculosis AND “systematic review” in Title/Abstract on 15/08/2024 returned 31 results of which 8 reviews were relevant. |
| **Mental health disorders** | Pubmed search for (“mental health” OR “mental disorder” OR “anxiety” OR “depression”) AND tuberculosis AND “systematic review” in Title/Abstract on 15/08/2024 returned 43 results. |
| **Undernutrition** | Pubmed search for (“undernutrition” OR “underweight” OR “nutrition” OR “body mass index”) AND tuberculosis AND “systematic review” in Title/Abstract on 15/08/2024 returned 59 results. |

Footnotes: * Pre-specified topics were multimorbidity and the chronic conditions/risk factor clusters named in the World Health Organization (WHO) Global Tuberculosis Report and WHO document on collaborative action for tuberculosis (TB) and comorbidities.^1,2^ Additional conditions were added where needed. In addition to these searches, the WHO guideline database was reviewed. ^†^ Search terms were applied without language or date restrictions. ^‡^ This search was to identify literature on multiple chronic conditions among people with TB. ^§^ This excludes the preprint by Jarde at al, identified separately.

Table 4: Association between chronic conditions and tuberculosis

|  | **Global burden** | **Association with TB** | **Policies and evidence on TB integration** |
| --- | --- | --- | --- |
| **HIV** | Globally almost 40 million people are living with HIV, of whom two thirds are in Africa. Among 15–49-year-olds in Africa, HIV prevalence is 3.4%. As a result of global expansion of ART, HIV related deaths globally fell by 51% between 2010–2023. In Africa in 2023, 90% of people with HIV knew their status, 82% were receiving treatment and 76% had suppressed HIV viral loads. | In 2008 people living with HIV were at over 20 times higher risk of developing TB compared to those without, whilst between 1990 and 2008 the number of TB cases and TB-related deaths in sub-Saharan Africa tripled because of the HIV epidemic.^3^ More recent declines in TB incidence rates reflect the massive expansion of HIV testing and ART. According to WHO estimates, in 2022 671,000 people living with HIV developed TB and 167,000 (25%) died. Of those, 69% and 68% respectively lived in Africa. Of people with TB globally who knew their HIV status, 7.3% were living with HIV, 89% of those were on ART. In Africa, equivalent figures were 20% and 93%. The excess risk of TB among people with well controlled HIV on treatment is likely much lower than historic estimates, however TB remains a leading cause of death among people with HIV, as well as being independently associated with mental health conditions. | As described in the text, integration of TB and HIV care is guided by WHO policies and included in routine TB reporting tools.^4^ |
| **Hepatitis B&C** | Hepatitis B (HBV) and C (HCV) are chronic viral infections which cause liver disease and liver cancer; together they are estimated to have caused 1.1 million deaths in 2021. Recent innovations in treatment have meant that these conditions are now curable. | A recent systematic review described pooled prevalence of active HBV of 5.8% (95% CI 5.0–6.8) and HCV (10.3%; 95% CI 8.4–12.3) among people with TB; higher than general population estimates.^5^ People with HCV appear to be at higher risk of developing TB compared to those without HCV, perhaps reflecting the role of shared risk factors.^6^ Drug-induced liver injury, complicating TB treatment, is more common among people with HCV than those without.^7^ | Screening for HBV and HCV is not routinely recommended for people with TB, however HBV and HCV may complicate TB treatment (e.g. 6 times increased risk of drug-induced liver injury^8^) |
| **Smoking** | Globally, in 2020 an estimated 991 million people aged 15 years or older smoked tobacco (prevalence 29% among men and 5.2% among women). Among the 10 countries with the highest incidence of TB an estimated 35% of men and 2% of women smoke tobacco.^2^ Tobacco smoking is, itself, a leading preventable cause of death and contributes to multimorbidity, being a cause of multiple respiratory and cardiovascular diseases.^9^  In 2011 it was estimated that without sustained progress in TB and smoking prevention, 40 million potentially avoidable TB-related deaths would be attributable to smoking by 2050.^10^ | Systematic review evidence demonstrates that smoking increases risk of *Mtb* infection, TB^11^ and MDR-TB^12^, with an estimated 730 000 new episodes of TB attributable to tobacco use in 2020^13^. People who have diabetes and smoke have an elevated risk of TB compared to those with diabetes alone.^14^ In an analysis of national TB prevalence surveys current smoking was associated with an increased odds of TB after adjustment for age and gender (aOR 1.53; 95% CI 1.39-1.69).^15^ Notably, passive smoking was also associated with increased risk of *Mtb* infection (~3 times increased risk^16^) and TB, particularly among children, which may reflect increased infectiousness of the smoking TB index patient.^11,16,17^ During TB treatment, smoking increases the risk of poor TB treatment outcomes, TB recurrence and mortality,^18^ | There is limited systematic review evidence for smoking cessation during TB treatment^19,20^, however recent trials have shown that very brief advice, or other counselling and or medication-based interventions, during TB treatment can lead to sustained abstinence (for 15–82% of smokers^21–32^) and stopping smoking improves TB outcomes and prevents relapse.^22,33^ Tobacco control as integral part of TB management have been recommended by WHO and the Union since 2007^34^, whilst a multi-country study has demonstrated that this is feasible and inexpensive, at scale.^35^ |
| **Alcohol use disorders** | 400 million people (7% of the world’s population aged 15 years and older) live with alcohol use disorders. In 2019, 2.6 million deaths (4.7% of all deaths) were attributable to alcohol consumption; with over three times higher alcohol-related mortality among men than women.^36^ | Alcohol use disorders are associated with a three times increased risk of TB disease (pooled risk ratio from 38 studies: 3.33; 95% CI 2.14–5.19), accounting for 740 000 new TB episodes in 2020, mostly among men (population attributable fraction for TB, 13% among men and 1.7% among women).^2,37–40^ Lower level alcohol use is associated with a smaller increase in risk of TB (1.35–1.9), with a 12% increase in TB risk for every 10-20 gram increase in alcohol consumption. Mechanisms include immunosuppression, increased exposure to high-TB transmission environments (e.g. bars) and shared pathways of social isolation and lower socio-economic position. In Zimbabwe, alcohol use disorders have the second highest population attributable fraction for TB after HIV.^41^ People with TB who consume alcohol are twice as likely to have poor TB treatment outcomes (treatment failure, death, or loss to follow-up) and more likely to develop MDR-TB.^42^ | Regular monitoring and support are recommended among people with TB who also have alcohol use disorder.^2^ |
| **Drug use** | In 2021, 296 million people aged 15–64 years used psychoactive drugs, with an estimated 0.6 million attributable deaths, mostly through blood-borne viruses.^36^ | People who use drugs (both injecting and non-injecting) have an elevated risk of *Mtb* infection and TB.^43^ Mechanisms towards this include the association of drug use disorders with other TB comorbidities such as HIV, viral hepatitis and mental disorders; whilst people who use drugs are more likely to have been incarcerated which increases their likelihood of exposure to *Mtb*.^44^ | WHO recommends a comprehensive package of services to address infectious diseases (including TB) among people who inject drugs (including harm reduction services, TPT and integrated care).^2,45^ |
| **Chronic lung disease** | Chronic respiratory diseases, of which chronic obstructive pulmonary disease is the most common, are the third leading cause of death globally, with an estimated 454 million people living with chronic respiratory disease and 4 million deaths in 2019. Tobacco smoking is the most common underlying cause.  Silicosis is a specific form of lung disease (a pneumoconiosis) caused by inhalation of respirable crystalline silica. The global burden of silicosis is unclear. Exposure to silica dusts and thus silicosis is common in mining and other industries^46^ but under- reporting means silica-related morbidity and mortality is likely far higher than modelled estimates.^47^ | TB has a bidirectional association with chronic respiratory diseases, with the strongest evidence for respiratory diseases occurring as a consequence of TB. Previous TB is associated with a 1.3–6.7 times increased risk of COPD (n=9 studies^48^), 3.1–4.6 times increased risk of bronchiectasis (n=2 studies)^48^ and four times increased incidence of lung cancer.^49^ TB has been estimated to be the fifth leading attributable cause of COPD globally.^50^ Persistent symptoms are common after TB^51^, with half of people assessed after TB treatment having lung function impairment, and 25% having activity-limiting breathlessness.^49^  Conversely, there is strong evidence that pre-existing lung diseases increase risk of TB. Silicosis substantially increases TB risk (OR 4.01; 95%CI 2.88–5.58), and worsens TB outcomes^52,53^, multiplicative with HIV.^54^ COPD may increase TB risk (HR 1.4–4.1 from n=4 cohort studies in high-income countries),^55^ with a stronger association in countries with high TB incidence, among never smokers, and among younger adults (i.e. where other major causes of chronic respiratory disease are less common). This may reflect direct (e.g. impaired innate and cell mediated immunity after TB) or shared causal pathways. | WHO guidelines recommend screening for TB among workers exposed to silica, and for TPT among people with silicosis.^56,57^ Other chronic lung diseases are not included. |
| **Mental health disorders** | Lifetime burden of mental disorders in adults are 12–49%, with 14% of the global disability-adjusted life years attributable to mental health disorders. Almost three quarters of the global burden of mental health disorders is in LMICs, where fewer than 10% of people with mental disorders receive mental health care. The stigma directed towards people with mental health disorders exacerbates mental health conditions, increases poverty, and prevents effective care and recovery.^58^ | Anxiety and depression are common among people with TB (prevalence of depression 9%–84% and anxiety 2%–47%^59,60^, higher among people with drug resistant TB^61^), with 9% of people with TB having suicidal ideation and a 1% suicide rate within two years from TB diagnosis.^62^ Co-morbid mental health disorders worsen health status compared to TB alone.^63^ Depression and schizophrenia are associated with increased risk of TB^64^, with a ‘dose dependent’ relationship (increased depression severity: HR 2.62 [1.74–3.96]), likely to reflect multiple causal pathways mediated by immunity, nutrition, smoking and alcohol misuse.^65^ At the same time, TB can cause mental health disorders through biological and psychosocial pathways. In terms of TB diagnosis and treatment, mental health disorders are associated with delays in seeking care, lower adherence^66^, worse TB treatment outcomes^67–69^ and worse health status^63^; therefore they represent a barrier to TB elimination by driving TB mortality and community transmission.^70^ | Mental health is increasingly a global development priority, and previous studies have suggested a high receptivity to TB/mental health integration among policymakers, programmers and healthcare workers.^71–74^ However policies, guidelines and training through which to implement this were felt to be lacking. Psychosocial interventions significantly improve adherence, TB outcomes and symptoms of anxiety and depression among people with TB^75^ and these are being implemented in LMICs through the WHO mhGAP programme^58^, with recent specific guidance on mental health care for people with TB.^76^ Mental health interventions for TB need to recognise syndemic interactions with poverty, which result in other fundamental and urgent priorities whilst curtailing individuals’ ability to ‘solve’ problems.^77^ |
| **Diabetes** | Globally, over 530 million people are living with diabetes, with prevalence expected to increase by 50% between 2019 and 2045 (and almost double in high-TB burden countries).^2,78^ Over 50% of people with diabetes around the world are undiagnosed and less than a third achieve good control of their condition. | Fifteen percent of people with TB globally have diabetes (~1.5 million people), with TB-diabetes comorbidity a particular concern in WHO Western Pacific and South East Asian Regions.^69,79^ Diabetes increases risk of *Mtb* infection^80,81^ and TB (OR range 1.5–3.6^82–85^ / pooled HR 1.90^86^) mitigated by good diabetes control.^87,88^ The effects of TB and its treatment may make diabetes control worse (e.g. through impaired glucose tolerance and hunger^89^). People with diabetes and TB have a higher risk of death, relapse and MDR-TB^90,91^, compared to those with TB alone; amongst people without HIV one in 10 TB deaths is attributable to diabetes^79^. A modelling study for 13 high-TB incidence countries suggested that maintaining current diabetes incidence (as opposed to the currently projected increases) could prevent 6 million TB cases and 1 million TB deaths in the next 20 years.^92^  Of note, transient hyperglycaemia due to TB can result in misclassification of diabetes status if cross-sectional screening is performed at the time of TB diagnosis.^93^ | Since 2011, the WHO Collaborative Framework for Care and Control of Tuberculosis and Diabetes has recommended ‘bidirectional screening’ of all people with TB for diabetes; and all people with diabetes for TB.^94^ However, in a recent survey of TB care providers across 27 countries only 52% reported that diabetes was routinely screened for among people with TB in their setting. There is substantial evidence on effective models of TB-diabetes integration.^95^ |
| **Cardiovascular diseases** | Cardiovascular diseases are the leading cause of death globally. Historically prevalence has been higher in high-income countries with older populations, however three quarters of global cardiovascular mortality is now in LMICs. In 2019, a third of all deaths globally were attributable to cardiovascular disease, with the vast majority of those being heart attacks and strokes.^96^ | No demonstrated association of hypertension with TB; in fact people with TB appear to have less hypertension compared to those without, both during^97^ and after treatment.^98^ During TB illness, hypertension may be masked by acutely lowered blood pressure, which later increases (TB sequel, unpublished). However, people with TB appear to have a higher risk of major cardiovascular events (RR 1.51; 95%CI 1.16–1.97; n=8 studies, all with high risk of bias).^99^ Other cardiovascular effects of TB may include long-term sequalae of cardiac involvement (e.g. pericarditis); these effects are not well described.^97^ Pregnant women with TB are at excess risk of pre-eclampsia.^100^ The increased risk of cardiovascular events among people with chronic lung diseases (particularly during acute exacerbations), may be one mechanism for the increased cardiovascular mortality seen among people with previous TB. | In settings where calcium intake is low, pregnant women with TB should receive calcium supplementation to prevent pre-eclampsia.^100^ No other policy recommendations exist. |
| **Undernutrition** | 309 million people globally are estimated to be living with acute hunger.^101^ Ten percent of TB-affected households living with severe food insecurity. Undernutrition remains a leading cause of death among young children, being a factor in 50% of under-5 deaths, whilst overweight and obesity represent a rapidly expanding epidemic of malnutrition. In 2022, whilst 390 million adults aged 18 and older were underweight, 2.5 billion were overweight or obese. Poverty amplifies the risk of, and risks from, malnutrition. | Undernutrition is the leading attributable cause of TB globally (responsible for 19% of global TB cases in 2020^13^) The relationship between TB and undernutrition is bidirectional and multifaceted.^102^ Lower body mass index is associated with an increased risk of TB, with a two-fold risk of TB among people who are underweight.^103^ Among people of normal BMI, the relationship is log-linear (with a 14% decreased risk of TB per unit increase in BMI^104^). Longer duration of underweight is associated with higher TB risk.^105^ Food insecurity itself has been associated with an increased risk of TB (e.g. Ethiopia: OR 2.38 [95%CI 1.52-3.73]). Undernutrition is associated with twice the risk of death during TB treatment^106,107^ and increased risk of TB recurrence^108^, whilst greater gain in weight during treatment is protective. Causal pathways include impaired cell-mediated immunity^109^ increasing progression from *Mtb* infection to TB, rather than through differences in *Mtb* infection risk^110,111^. It is likely that micronutrient, as well as macronutrient, insufficiency is important^100^, however studies providing supplementation of individual micronutrients (zinc, vitamin A, or vitamin D) have generally been inconclusive.^25-28^ TB can lead to appetite reduction, malabsorption, and impaired metabolism, in turn resulting in undernutrition and unintentional excessive weight loss (cachexia).^112^ | Screening of nutritional status, dietary counselling and management of moderate-severe acute malnutrition (defined using and in accordance with other guidance) is recommended by WHO for people with TB, whilst severe acute malnutrition (SAM) should be managed in accordance with relevant WHO guidelines.^100,113^ Children with SAM should be screened for TB. Additional counselling, and consideration of nutritional support is recommended for people with TB who fail to gain weight by two months of treatment, or for pregnant women and people with MDR-TB. In the RATIONS trial, people with TB who gained the most weight had the lowest mortality.^114^ Social incentives which promote food security improve TB adherence and outcomes. |
| **Other disability** | Disabiliy is defined as an impairment in physical or mental function that results from the interaction of health conditions with personal or environmental factors, including age, gender, social values, access to infrastructure and policies. An estimated 16% of the world’ population is living with a disability.^115^ | Physical disabilities related to TB vary according to the bodily site affected. Other common disabilities during TB treatment include musculoskeletal impairment (17.1%), hearing impairment (14.5%, more common among people with DR-TB), visual impairment (9.8%), renal impairment (5.7%), and neurological impairment (1.6%).^61^.  Overall, one in four people with TB are left with some disability after treatment completion.^116^ Some of those may be attributable to the anti-TB drugs (specifically injectables for MDR-TB which are no longer in use, and linezolid). | No specific guidance exists for management of TB-associated disability however WHO recognises that TB-associated impairments require a holistic, multidisciplinary approach that includes preventive and rehabilitation services; and that such services are not often available in LMICs with a high burden of TB.^116^ |

Abbreviations: 95% CI = 95% confidence interval; ART = anti-retroviral therap**y**; (M)DR-TB = (multi-)drug resistant tuberculosis; Mtb = mycobacterium tuberculosis; LMIC = low and middle income countries; OR = odds ratio; WHO = World Health Organization.

Table 5: A timeline of United Nations / World Health Organisation declarations, policies and guidelines of relevance to integrated tuberculosis screening and care

| **Year** | **Quotes** |
| --- | --- |
| 2004 | *“HIV testing and counselling should be offered to all tuberculosis patients in settings where the HIV prevalence among tuberculosis patients exceeds 5%.”*^117^ |
| 2007 | *“Opportunities must be created within the health care system to provide every TB patient who is a smoker encouragement and help to overcome the tobacco addiction. In addition every TB patient who is not a smoker must be made aware of the consequences of being exposed to secondhand smoke.”*^34^ |
| 2010 | *“HIV testing for patients of all ages who present with signs or symptoms that suggest tuberculosis (7), whether TB is suspected or already confirmed…* *Testing and counselling should be recommended for children and other immediate family members of all people living with HIV, in cases where horizontal or vertical transmission may have occurred…. Serodiscordant partnerships provide an important opportunity for prevention of HIV transmission.”*^118^ |
| 2011 | *“Patients with TB should be screened for diabetes at the start of their treatment, where resources for diagnosis are available.”*^94^ |
| 2012 | *“Routine HIV testing should be offered to all patients with presumptive and diagnosed TB… Partners of known HIV-positive TB patients should be offered voluntary HIV testing and counselling with mutual disclosure… TB-control programmes should implement comprehensive HIV prevention strategies for their patients and their partners, targeting sexual, parenteral or vertical transmission or should establish a referral linkage with HIV programmes to do so”*^4^ |
| 2013 | *“Because of the clear bidirectional causal link between undernutrition and active TB, nutrition screening, assessment and management are integral components of TB treatment and care… Poverty and food insecurity are both causes and consequences of TB, and those involved in TB care therefore play an important role in recognizing and addressing these wider socioeconomic issues…. TB is commonly accompanied by comorbidities such as HIV, diabetes mellitus, smoking and alcohol or substance abuse, which have their own nutritional implications, and these should be fully considered during nutrition screening, assessment and counselling.”*^100^  *“All individuals with active TB should receive (i) an assessment of their nutritional status and (ii) appropriate counselling based on their nutritional status at diagnosis and throughout treatment. In settings where contact tracing is implemented, household contacts of people with active TB should have a nutrition screening and assessment as part of contact investigation. ”*^100^  *“Addressing comorbid conditions has value for improving access and response to TB treatment and it should be considered as part of the standard of care for people with TB. The aim of comprehensive care should be to improve general health and quality of life.”*^100^ |
| 2015 | *“All persons with TB need to be assessed for nutritional status and receive nutritional counselling and care according to need. All persons with TB should also be screened for diabetes. Further, depending on local epidemiology, all persons with TB should be assessed for other co-morbidities and related risk factors such as smoking and alcohol or drug abuse.”*^119^  *“In addition to HIV/AIDS, other co-morbidities and health risks associated with TB are important and require integrated patient management. This includes undernutrition, diabetes, alcohol or drug abuse, smoking, silicosis, chronic obstructive pulmonary disease (COPD) and other non-communicable diseases including mental health problems… Relevant co-morbidities and health behaviours should be routinely assessed and managed for improved TB treatment and general health outcomes.”*^119^ |
| 2018 | *United Nations member states “Commit to developing community-based health services through approaches that protect and promote equity, ethics, gender equality and human rights in addressing tuberculosis by focusing on prevention, diagnosis, treatment and care, including socioeconomic and psychosocial support, based on individual needs, that reduce stigma, and integrated care for related health conditions, such as HIV and AIDS, undernutrition, mental health, non-communicable diseases including diabetes and chronic lung disease, and tobacco use, harmful use of alcohol and other substance abuse, including drug injection, with access to existing and new tools”*^120^ |
| 2022 | *“Efforts to achieve UHC also place a special emphasis on the poor, vulnerable and marginalized segments of the population, many of whom are at elevated risk of experiencing TB and comorbidities. Therefore, to improve health for all, collaborative action on TB and comorbidities should be aligned with and feed into the national UHC agenda.”*^2^ |
| 2023 | *“Social support, including education and facilitating access to psychological and material support, is critical to mitigate the impact of poverty, TB, its treatment, and the related stigma and discrimination, on people’s mental health.”*^76^ |

Table 6: Strategies used to identify relevant literature on chronic conditions among members of TB-affected households

| **Topic** | **Search strategy** |
| --- | --- |
| NCDs | No formal search; recent systematic review already identified.^121^ Conditions of interest for this review were diabetes, hypertension, renal disease, cardiovascular disease, chronic respiratory disease, dyslipidaemia, cancer, and mental health conditions. |
| Nutrition | Pubmed search for undernutrition or obesity, and tuberculosis, and household in Title/Abstract on 20/02/2024 returned 34 records; citations of recent systematic review of association between undernutrition and TB additionally considered (08/2024).^103^ |
| Alcohol | Pubmed search for alcohol AND tuberculosis AND “household?” in Title/Abstract, last updated 9/8/2024 identified 60 records, of which 6 were relevant. |
| HIV | Systematic searches were performed, as reported separately.^122^ |

**Abbreviations:** NCDs = non-communicable diseases.

Table 7: Key Findings on Non-Communicable Diseases, Mental Health, and Nutrition among TB Household Contacts

| **Category** | **Key Findings** |
| --- | --- |
| Diabetes, hypertension, and smoking* | Higher odds of smoking among household members of TB-affected individuals in TB prevalence surveys.^123^ In systematic review, among household contacts 7.3% prevalence of diabetes (n=4 studies), 11.4% hypertension (n=4 studies). measured diabetes prevalence was twice that determined using self-report.^121^ |
| Mental health disorders | Systematic review: prevalence 22% (n=1 study).^121^ |
| Alcohol use disorder | No systematic review data. Across several studies (Nigeria, Ethiopia, Myanmar, Nepal, India) prevalence of alcohol use among tuberculosis household contacts ranged from 2.4% to 12.9%, with an increased risk of co-prevalent tuberculosis among household contacts who drank alcohol.^124–129^ No data on incident TB risk or harm reduction interventions among household contacts. |
| Undernutrition | Studies in India have described a high prevalence of underweight among household contacts (16–35%^111,129,130^). Underweight household contacts had a 1.88–6.16 times higher risk of developing TB compared to those with a normal body mass index^111,129^; consistent with findings from a systematic review of cohorts (not only household contacts).^103^ Food supplementation was associated with a 43% reduction in TB incidence among household contacts.^131^ |

**Footnotes:** * No studies identified evaluating chronic lung disease among tuberculosis household contacts in the review by Hamada et al.^121^

## Delphi survey design and distribution

We selected a consensus exercise, and specifically a modified Delphi method (i.e. using online, as opposed to in person, tools), given that we sought to capture the perspectives of a diverse range of experts, across geographic regions and multiple individual conditions where often randomised controlled trial data does not yet exist. Anonymity between participants, and equal weighting of all panellists in analysis ensures that people with relatively less power and influence are equally heard.

We chose to disseminate the survey widely, using a range of strategies, and consider responses from anyone who self-identified (after reading the study information) as having expertise in tuberculosis, non-communicable diseases or integrated care, and who wished to contribute. Tuberculosis survivors and members of affected community were explicitly named as a key group of panellists in the information provided to participants. This approach aimed to increase inclusivity and representativeness of the resulting recommendations. We did not stop accepting responses after a pre-specified sample size as we considered that later responses may improve inclusivity, offering additional perspectives compared to those of people reached through more immediate networks. Our approach differed from more traditional Delphi surveys where a limited, predefined panel of experts, identified by the researchers, are invited to participate. This latter approach can tend towards an elitist view of expertise and is less likely to identify divergent views by the nature of a bias towards the researchers’ own networks.^132^ We aimed to receive at least 20 responses from each world region.

Survey invitations were issued by purposive recruitment via email, using known networks of people, including as part of research groups, Union working groups, national tuberculosis programmes, implementing organisations, and civil society mailing lists. We invited individual members of community advisory boards (CAB) to participate via CAB co-ordinators. We sent personalised emails where possible and asked invitees to forward the invitation to other people in their networks, with a focus on reaching local policy and health professionals and tuberculosis-affected community. In round two we additionally asked invitees to consider forwarding the invitation to people from regions of the world under-represented in the previous survey round. For round 1, one email reminder was sent part-way through the data collection period (11^th^ July 2023 to 5^th^ September 2023). For round 2, all participants received one reminder part way through the survey window (10^th^ May 2024 to 20^th^ July 2024); people who had participated in round 1 received two reminders.

We also sought to be inclusive through our survey design, with most questions included in the first-round survey asking for open ended, free-text responses. This allowed for divergent and unexpected views, in comparison to the approach of including a pre-determined list of outcomes. We monitored aggregated responses in real time and iteratively refined who we invited to participate to ensure broad representation. As we sought to improve representation from tuberculosis/tuberculosis-multimorbidity-affected community and across world regions in round 2 (see below), we invited by email both people who had participated in round 1 of the survey, and purposively selected other potential panellists. We also included free-text boxes spaced throughout the round 2 survey, inviting panellists to leave comments and expand on their responses to the consensus statements.

The round 1 survey was available in English, French, Spanish and Portuguese. For round 2, the survey was additionally available in Vietnamese. Translations were performed by native speakers of each language. Each survey was piloted in English by at least 2 people and in each translated language by at least one person. All individuals participating in the piloting were tuberculosis researchers. Pilot responses were not included in the analysis; however, the individuals who completed the pilot were invited to also complete the final survey. We considered, but ultimately did not include, a Russian translation. This was based on previous experience of a survey on the impact of COVID-19 on tuberculosis services, where most respondents from Russian-speaking countries elected to respond in English.^133^

Panellists were blinded to the identity of all other panellists throughout the survey.

## Data analysis

Panellists were able to indicate that they lived and worked in more than one country and identify themselves as a member of as many professional groups as were relevant. For stratification of responses by professional category and WHO region, we reviewed responses where multiple options had been selected and assigned the most appropriate category. People who indicated that they lived/worked in more than one world region were usually global health researchers working (usually) across Europe/North America and a high-tuberculosis burden region; in these cases panellists had responded about the high-tuberculosis burden region in which they worked. They were thus assigned to this region.

We developed four categories for profession: i) healthcare workers; ii) policy makers, public health professionals, M&E specialists; iii) researchers; iv) tuberculosis survivors, people with chronic conditions and civil society advocates. Most panellists fell naturally into one of these categories. People who identified as a member of tuberculosis -affected community (tuberculosis survivors, people with chronic conditions and civil society advocates) and another role were assigned as the former. People who were both doctors and researchers (a common combination) were considered as Researchers. Three people could not be categorised into one of these groups and thus were excluded from analyses stratified by professional group.

For thematic analysis, we adopted a grounded theory approach, considering each group of questions in turn (as often panellist responses referred back to previous answers). Within each group of questions, an inductive approach to coding and identification of emergent themes was employed. Coding of responses was performed by CJC, MC and TK in NVivo. Given the number of responses, each was coded by one person, whilst discussion between researchers clarified codes and themes. Review of emergent themes on key prerequisites, including barriers and enablers to implementation highlighted commonality with the World Health Organisation health system building blocks and thus emergent themes were mapped to the relevant domain.^134^

To calculate weighted average percentage agreement, we calculated the percentage of panellists who agreed with the statement posed, stratified by i. WHO region, ii. Individual country or iii. Professional category. For stratification by country, panellists who indicated that they lived/worked in more than one country were excluded. Percentages were summed across strata and divided by the number of strata to give a average percentage agreement in which each stratum received equal weight.

Where categories include fewer than 5 responses, individual data are not shown.

## Refinement of the approach in response to round 1 findings

Review of the data from round 1 demonstrated relatively few responses from members of tuberculosis-affected community and people from WHO Western Pacific and South East Asian Regions. As high tuberculosis incidence regions, this omission was important and could impact the reliability and representation of the expert consensus. In addition, affected people (i.e. tuberculosis survivors or people with chronic conditions) were the least represented group. We thus developed two steering committees, one representing global tuberculosis experts with a specific focus on people working in the WHO Western Pacific and South East Asian Regions, and the other representing tuberculosis-affected community. Through online meetings and email discussion, the two steering committees reviewed the round 1 analysis and interpretation of the round 1 data and gave input into the data summary and questions for round 2, with a particular emphasis on improving accessibility of the survey for members of affected community. These consultations also led to the inclusion of questions about community-wide tuberculosis screening which had not been included in the round 1 survey. We also created an online video which explained the Delphi process and summarised the results of the first round of the survey to increase accessibility^135^ and additionally translated the survey into Vietnamese. The decision to translate the survey into Vietnamese was informed by i) wanting to increase responses from South East Asia and ii) Vietnam being a high burden tuberculosis country. We also specifically asked four research groups we knew to have local community advisory boards to arrange to complete the survey with their members, providing Microsoft Word copies for paper completion. These were manually entered and merged with the rest of the responses.

Fig 1: Flowchart summarising Delphi survey development and conduct

**Footnotes:** * We asked invitees to forward the invitation to other relevant people within their networks (snowball sampling), with one of the researchers (TK) in carbon copy. This aimed to enable us to estimate the total reach of the survey, however it is likely that we were not copied into all forwarded emails and hence we were not aware of some people who were invited through snowball sampling.

# Supplementary results

Table 8: Delphi panellist demographic details

| **Characteristic** | | **Round 1  N = 223** | **Round 2  N = 324** |
| --- | --- | --- | --- |
| **Gender** | Man | 120 (54%) | 158 (49%) |
|  | Woman | 100 (45%) | 158 (49%) |
|  | Other/not disclosed | 3 (1.3%) | 8 (2.5%) |
| **Employing/affiliated organisation** | Academic or research organisation | 126 (57%) | 148 (46%) |
|  | Community based organization | 13 (5.8%) | 29 (9.0%) |
|  | International NGO | 33 (15%) | 64 (20%) |
|  | Local NGO | 29 (13%) | 53 (16%) |
|  | National TB programme | 28 (13%) | 30 (9.3%) |
|  | Ministry of Health | 23 (10%) | 24 (7.4%) |
|  | Private healthcare provider | 13 (5.8%) | 9 (2.8%) |
|  | Public healthcare provider | 25 (11%) | 45 (14%) |
|  | World Health Organization | 6 (2.7%) | 9 (2.8%) |
|  | Other or none | 8 (3.6%) | 18 (5.6%) |
| **Profession/role** | Public health professional | 98 (44%) | 120 (37%) |
|  | Policy professional | 10 (4.5%) | 14 (4.3%) |
|  | Civil society advocate | 21 (9.4%) | 34 (10%) |
|  | Patient survivor | 13 (5.8%) | 19 (5.9%) |
|  | Researcher | 117 (52%) | 146 (45%) |
|  | Doctor | 106 (48%) | 145 (45%) |
|  | Nurse | 10 (4.5%) | 25 (7.7%) |
|  | Community healthcare worker | 3 (1.3%) | 17 (5.2%) |
|  | Other healthcare worker | 5 (2.2%) | 7 (2.2%) |
|  | Laboratory scientist | 16 (7.2%) | 19 (5.9%) |
|  | Monitoring and evaluation specialist | 11 (4.9%) | 21 (6.5%) |
| **WHO region** | Africa | 115 (52%) | 122 (38%) |
|  | Americas | 40 (18%) | 40 (12%) |
|  | Eastern Mediterranean | 9 (4.0%) | 21 (6.5%) |
|  | Europe | 33 (15%) | 62 (19%) |
|  | South-East Asia | 13 (5.8%) | 26 (8.0%) |
|  | Western Pacific | 13 (5.8%) | 53 (16%) |
| **Footnotes**: N = number of panellists. Panellists were able to select more than one gender, employer/affiliated organisation and profession/role. World Health Organization (WHO) regions were assigned from countries in which panellists were employed or based, as reported. *Other responses were in Russian (with the questions in English). **Abbreviations**: NGO = non-governmental organisation. | | | |

## Round 1 consensus: dissenting voices

In round 1 of the survey, only 1.8% of panellists (n=4) would not consider screening people with tuberculosis for non-tuberculosis conditions or offering them non-tuberculosis services. Of the four people who said they would not consider this, three gave reasons. Two respondents said that patients were either already evaluated (or should be evaluated) for common conditions at primary care, whilst a third cited the lack of inclusion of such services in their local NTP guidelines.

Eighty-five percent of panellists would consider screening for tuberculosis household contacts; 15% would not (n=34). Most panellists who would not consider screening for non-tuberculosis conditions or providing non-tuberculosis services expected such services to be available elsewhere. Amongst those who would not consider screening for non-tuberculosis conditions among household contacts, the main concerns were about practicality of care delivery and lack of current evidence and guidelines to support this practice. Some dissenting panellists suggested that additional non-tuberculosis services should be offered selectively to tuberculosis household contacts (n indicates number of responses coded to this theme):

- - “It will consume huge costs and it will not be practical/ realistic” (n=14)
  - “It is not the current standard of care” (n=4)
  - “Acceptability amongst families of people with tuberculosis has not been established” (n=4)
  - “I would only screen for HIV/mental health and case-by-case” (n=3)
  - “I don’t know enough about the potential benefit” (n=3)
  - “Distracts from routine tuberculosis services” (n=2)
  - “There should be other services available to do this outside tuberculosis” (n=1)

## Development of consensus statements

### Rationale for integrated tuberculosis screening and care services

Next, panellists were asked a series of questions on why they had suggested the services or conditions mentioned above. Thematic analyses of these questions aimed to establish the underlying rationale for the selection. There was substantial overlap in the themes identified on the rationale for integrated tuberculosis screening and care services across questions on people with tuberculosis and household contacts and therefore these are broadly summarised together (Table S10).

For many panellists the often-bidirectional relationships between tuberculosis and other conditions, multiplying the adverse effects of each, was central to their reasons for providing integrated care, with the underlying goal of such services being to impact tuberculosis-specific outcomes (e.g. improving tuberculosis treatment adherence, tuberculosis treatment outcome or post-tuberculosis morbidity; and reducing tuberculosis incidence among household contacts). Several panellists suggested that conditions which do not have an association with tuberculosis or do not impact tuberculosis-specific outcomes should not be screened for, particularly if resources are scarce. Conversely, other panellists highlighted the *“opportunity”* that a tuberculosis diagnosis presents for vulnerable individuals or families to access healthcare, when they might otherwise not do so, particularly in a context where services are limited or not available outside of the tuberculosis programme. Similarly, panellists identified integrated services as an opportunity to fulfil the mandate of holistic, person-centred care for both people with tuberculosis and their support network. An indirect potential mechanism to improving tuberculosis-specific outcomes (thus perhaps justifying inclusion of conditions less directly associated with tuberculosis) was via a reduction in the stigma experienced by people affected by tuberculosis due to integration of tuberculosis screening or care with other services that are less stigmatised. This was proposed to increase perceived benefits and increase acceptability and thus uptake of tuberculosis screening; however, a potential hazard may be jeopardising the tuberculosis service through inclusion of services that exacerbate stigma. A few (<10) panellists also highlighted the general principal of reducing *“siloed care”* and potential for integrated tuberculosis care to contribute to health worker capacity development with broader benefit.

The epidemiology of the (non-tuberculosis) conditions in the wider community and local health priorities were also seen to be relevant factors. Local community prevalence was a common consideration among both people with tuberculosis and screening for household contacts. This was also apparent in panellists’ suggestions for approaches by which to target screening and care services (56% [n=124] suggested prioritisation of sub-groups), for example suggesting that this should be offered to children (n=24), adults (n=73), older adults (n=38), men (n=2), prison populations (n=4), miners (n=7), smokers (n=2), people with multi-drug resistant tuberculosis (n=2) or other high risk groups (n=17).

Some panellists identified tuberculosis as a *“complex problem”* most impacting vulnerable and marginalised people, and noted the shared genetic, environmental and behavioural risks between people with tuberculosis and household contacts as a reason for household contacts to be a priority group for public health interventions. The psychological and economic burden of tuberculosis, shared within the family, was noted in particular. Given the role of family in supporting people with tuberculosis holistic care for families was seen as potentially improving tuberculosis treatment outcomes.

Whilst panellists highlighted the potential for screening to improve early detection of health conditions, several feasibility concerns were raised. These included the need for simple, point-of-care, low-cost tests and the imperative to have available and accessible referral or treatment services. In situations where these conditions are met, integration could address some of the *“low hanging fruit”* in challenged health systems. Some panellists warned of the risk that a *“divided focus”* would undermine core tuberculosis programme activity and potentially reduce the quality of tuberculosis care. Of note, mental health was one condition identified to be important, cheap and feasible to screen for, but some panellists suggested it could not be included because of the lack of accessible care services. In the household contact screening setting privacy must be assured (e.g. for HIV and sexual and reproductive health services). Whilst integration of services was perceived to be a cost-effective strategy through economy of scope, panellists questioned its affordability within current tuberculosis budgets and emphasised the need to understand the costs (ensuring cost-effectiveness), resource requirements and potential inefficiencies generated by such a programme.

These same considerations fed into panellists’ suggestions for conditions which should not be included in an integrated tuberculosis care or screening context, including suggestions to exclude conditions that are not highly prevalent (n=11), those that are not a local priority (n=8), or those that do not affect tuberculosis treatment outcomes (n=10). Other key considerations related to feasibility and the capacity of the health system to provide services.

Table 9: Rationale for offering non-tuberculosis screening or care services to people with tuberculosis and household contacts (survey round 1)

| **Theme** | **Sub-themes** | |
| --- | --- | --- |
|  | **People with tuberculosis** | **Household contact screening** |
| Other conditions are associated with increased TB risk, worse TB outcomes, or occur because of the impacts of TB | The condition has high prevalence amongst people with TB (n=62); do not screen if it doesn’t (n=11).  The condition has a multiplicative / interactive effect on TB outcomes (n=62)  TB treatment causes or exacerbates the condition (n=22)  The condition increases TB risk (n=32), particularly if it is a modifiable risk factor (n=6) or if TB is a risk factor for the condition (n=16) | Non-TB conditions increase risk of TB disease and poor outcomes in household contacts (n=45)  Household members share genetic, environmental and behavioural risk factors with people with TB (n=16)  It will improve detection and management of TB co-morbidities (n=10)  Conditions that are not a TB risk-factor nor relevant to TB screening, diagnosis or treatment, should not be screened for, particularly if resources are scarce (n=25) |
| It improves outcomes for the person with TB, and/or lessen the impact of TB on people and families | Improving the condition improves TB outcomes (n=30); do not screen for conditions that do not affect TB outcomes (n=10)  Addressing condition promotes behaviour change and improves treatment adherence (n=18)  Treating other condition(s) increases TB recovery/social participation (n=14)  Untreated condition impairs/ complicates TB treatment and management (n=12)  Interventions for common TB and other condition risk factors improve both conditions (n=6) | Household members experience psychological and socioeconomic burden because of TB in the family (n=10)  Holistic care to families can improve TB treatment adherence by the index case (n=6)  Patient can be treated and managed better if they have good psychological and social support from families (n=4) |
| There is a high prevalence of non-TB conditions in the general population | The setting has a high prevalence of both the other condition and TB (n=36) | Other conditions are highly prevalent in area (n=9), meaning screening has high yield (n=11)  Consider only screening specific sub-groups (n=2 – do not screen for NCDs in children) |
| There is an opportunity to do so | TB services are an opportunity to improve the lives of vulnerable people (n=16)  TB serves as an entry-point to the healthcare system and can improve access to care for other conditions (n=14)  Screening improves early detection / diagnosis of other conditions (n=14)  There is no screening or care available for comorbidities unless offered by the TB program (n=4)  This is an opportunity to screen / treat neglected diseases (n=4) or include screening for priority conditions according to local authorities (n=4) | We should make most of the opportunity to reach vulnerable people who may not otherwise access care (n=17)  Early detection of other conditions in household members can save lives (n=17)  Incidence of other conditions is rising and these should be screened for (n=5) |
| It may maximise use of resources and be cost-effective | Screening for additional conditions improves the cost-effectiveness of screening (n=12)  Do not screen if doing so is overly complex or too specialized to offer in the routine setting (n=21), is not cost-effective (n=9), or creates inefficiency in patient care (n=7) | Integrated screening is often cost-effective; it should only be done in this case (n=11)  Some interventions are easy add-ons that are not resource intensive (n=7) |
| There is an imperative or opportunity to provide high-quality, holistic, person-centred care | It fulfils a mandate to provide holistic, person-centred care (n=16)  It will improve quality of care (n=14)  It will reduce siloed care (n=6) | It ensures maximum welfare and holistic care for the patients and their support network (n=10)  It is appropriate to address a complex problem with multi-level services (n=1) |
| It will reduce stigma and improve acceptability of TB care and screening | Integration of TB screening with screening for less stigmatised conditions reduces community / self-perceptions stigma (n=13) | It improves acceptability of screening among household members (n=2) and community (n=4*) due to higher perceived benefit  It improves uptake of screening due to inclusion of conditions prioritised by community (n=2*)  Do not screen for conditions that would jeopardise participation in TB screening i.e. invasive tests or stigmatised conditions (n=3) |
| It is included in guidelines / policies, and aligns with local priorities | Only offer screening / care if the non-TB condition is a local authority screening priority (n=6) | Screening for other conditions in household members is part of national guidelines (n=2)  Do not screen for conditions that are not local priority (n=2) |
| It is feasible to provide screening and onward treatment / care | Only offer screening / care if referral services are available (n=14)  Many tests for other conditions are very simple / easy to add-on (n=2) | Screen can be simple, treatment / referral services are easily accessible (n=14 – exclusions: cancer; lung health; mental health), and privacy can be assured in the setting (n=3 – exclusions: HIV; STI; SRH)  Results are available at point of care, minimising resource use in follow up (n=3 – cancer)  Do not screen if TB screening and care is compromised by divided focus (n=3) or if the number of services would overwhelm household members (n=2) |

**Footnotes**: number (n) of panellists for whom this sub-theme was reported. Sub-themes are ordered by the number of occurrences. * Some themes reported were felt to relate both to household contact screening and community-wide screening, prompting the inclusion of an additional section on community-wide screening in the round 2 survey. Abbreviations: NCD = non-communicable diseases; SRH = sexual and reproductive health; STI = sexually transmitted infections; TB = tuberculosis.

Table 10: Gender considerations in development of integrated tuberculosis screening and care (survey round 1)

| **Theme** | **N panellists** |
| --- | --- |
| ***Both groups*** | ***3*** |
| Include services (and deliver in a way) which will attract men | 3 |
| ***People with tuberculosis*** | ***19*** |
| Include services (and deliver in a way) which will attract men | 2 |
| Prioritise women of childbearing age/pregnant women for SRH services | 4 |
| Sex-specific conditions (i.e. cervical cancer, prostate cancer, pregnancy, contraception)* | 12 |
| ***Household contacts*** | ***19*** |
| Include services (and deliver in a way) which will attract men | 3 |
| Lower age threshold at which to screen for cardiovascular disease in men | 2 |
| Prioritise women of childbearing age/pregnant women for SRH services | 3 |
| Sex-specific conditions (i.e. cervical cancer, prostate cancer, pregnancy)* | 11 |
| **Total** | **41** |

**Footnotes:** * Most of these comments related to provision of pregnancy testing and or family planning for women with tuberculosis and household contacts; a minority (n=2) mentioned male-specific conditions (prostate cancer).

The identified themes were used to develop statements which captured the motivation (n=4 statements), principles (n=3 statements), and risks (n=2 statements) of integrated screening or care for people with tuberculosis and tuberculosis household contacts, respectively. We proposed seven criteria by which conditions and services could be selected for a package of integrated care. It was clear that how these criteria would apply to individual conditions and services would be different in different contexts and thus, rather than seeking to develop a list of services to be integrated, we developed a list of country-specific questions for round 2, in which we asked panellists to name up to three countries in which they were familiar with the tuberculosis programme; for each we asked about currently available services and for panellists to rank which of the top 11 conditions named in round 1 (above) they would include in a package of integrated care. Based on our literature review, there was limited evidence for many of the proposed justifications for integrated care; we thus included statements on evidence generation and funding.

### Services for inclusion as part of integration

Panellists were asked which conditions they would screen for and which services they would offer to people with tuberculosis or during household contact screening, respectively. A separate question asked about conditions and services panellists would not include in integrated services.

A total of 1082 suggestions of conditions or services to be offered to people with tuberculosis were made (Figure S2). When grouped by category, non-commumicable diseases (NCDs) and psychosocial needs received an equal number of mentions (~33% each), followed by communicable diseases (mostly HIV; 22%), nutrition (7.4%) and sexual and reproductive health services (2%). Four percent of suggestions fell outside these categories. Notably, among the 13 responses from members of tuberculosis-affected community, 50% (10/20 suggestions) were for psychosocial assessment and support.

Conditions and services suggested as being relevant for inclusion as part of screening for household contacts were similar to those for people with tuberculosis (641 individual suggestions; Figure S2). By category, 31% suggestions related to each of NCDs and psychosocial needs, 22% to communicable disease, 11% to nutrition, and 2% sexual and reproductive health. Four percent of suggestions fell outside these categories. Ten of the 28 conditions/services mentioned by members of tuberculosis-affected community related to psychosocial needs.

Few panellists named specific conditions and services they would not want to see as part of integrated services (n=30/223 named at least one among people with tuberculosis and 30/223 did so for household contacts). Of the conditions named, the most frequent mentions were NCDs and cancers (Table S8). Interestingly, in some cases the same condition was suggested for inclusion by some panellists and as something not to include by others (e.g. NCDs [8/29 mentions as a condition not to include among people with tuberculosis], and mental health [4/29 mentions among people with tuberculosis and 17/30 mentions among tuberculosis household contacts]).

Fig 2: Non-tuberculosis conditions for which experts would offer screening and care among people with tuberculosis and their household contacts, ranked by frequency of votes and reported as both total expert responses and by subgroup of tuberculosis survivor responses (survey round 1)


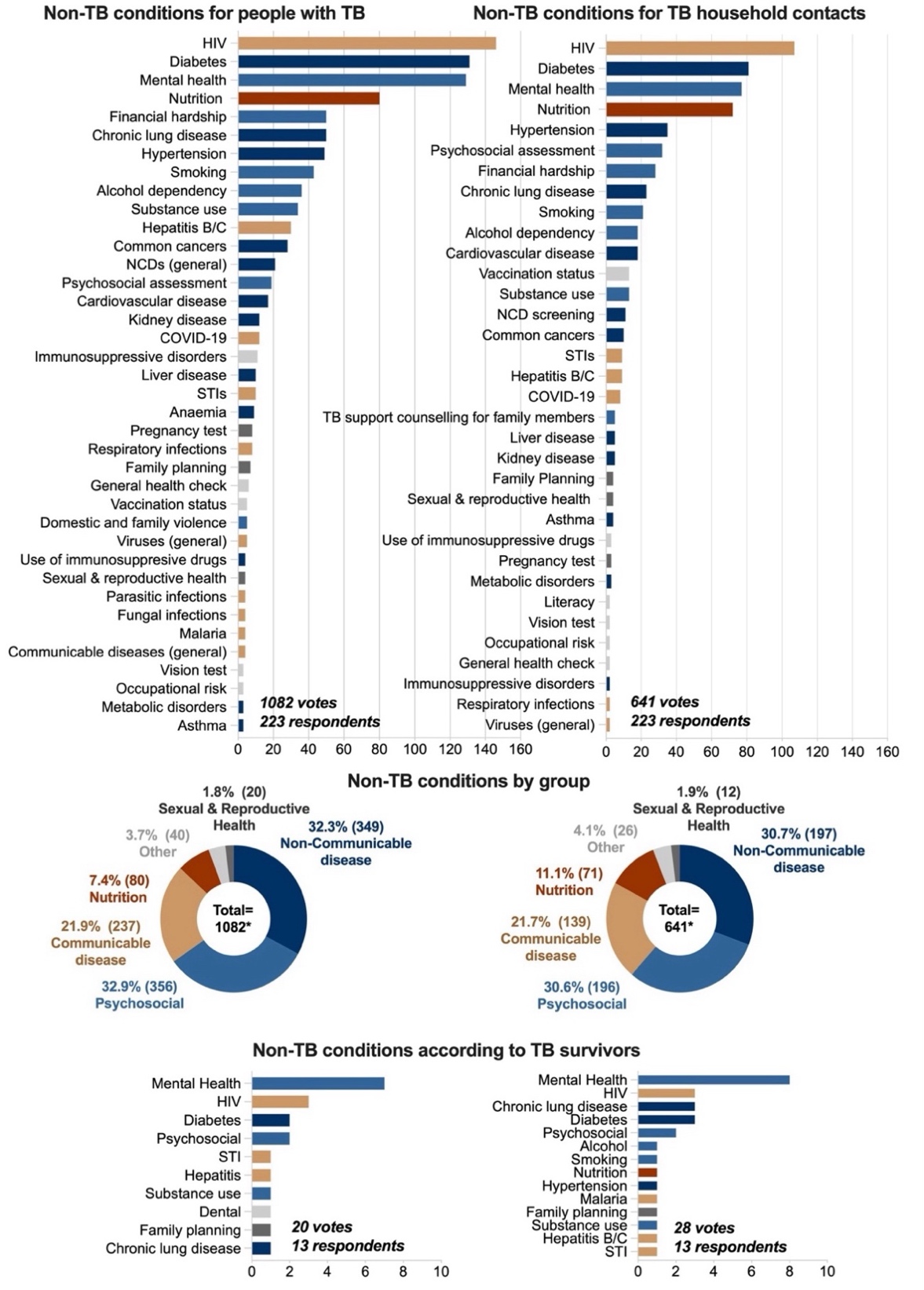


**Footnotes**: Left series of plots show responses for people with tuberculosis and right series of plots for household contacts. Data are from all panellists, other than in the last two plots which include people identifying as tuberculosis survivors only.

Table 11: Conditions mentioned by panellists as not being suitable for inclusion in integrated tuberculosis screening or care services, stratified by whether mentioned with regard to people with tuberculosis or household contacts (survey round 1)

| Conditions to NOT include | People with tuberculosis (n=29) | Household contacts (n=30) |
| --- | --- | --- |
| NCDs (including CVD and HTN) | 9 | 2 |
| Lung health | 2 |  |
| Cancers | 7 | 17 |
| Mental health | 4 | 2 |
| SRH services | 4 |  |
| Physical disabilities | 1 |  |
| Stigmatising conditions | 1 |  |
| Recreational drug use | 1 | 2 |
| Malaria (if no symptoms) |  | 3 |
| Dental |  | 2 |
| Genetic disorders |  | 2 |

**Footnotes**: Numbers shown in cells are the number of panellists mentioning this condition as something not to include within an integrated service (blank cells = no mentions); the column header summarises the total number of panellists (of total 223) who suggested a condition that should not be included. Abbreviations: NCDs = non-communicable diseases; CVD = cardiovascular disease; HTN = hypertension.

We also asked panellists whether they felt the services they had mentioned should be offered universally (to people with tuberculosis or household contacts, respectively), or targeted towards specific groups. Most comments here related to the need for risk stratification, by age (e.g. targeting screening for diabetes and hypertension towards older adults; n=52 [people with tuberculosis] and n=64 [household contacts]), sex/gender (n=10 [people with tuberculosis] and n=9 [household contacts]) or other participant characteristics (e.g. prisoners (n=2 [people with tuberculosis]), miners (n=3 [people with tuberculosis] and n=4 [household contacts] or unspecified *“high-risk groups”*).

We reviewed responses related to sex and gender more broadly: 41/223 (18%) panellists commented on an element of sex/gender in any part of their response, as summarised in Table S9 (below).

Table 12: Themes relating to the motivations (and concerns) for integrated TB screening and care, from free text comments (survey round 2)

|  | Theme | N |
| --- | --- | --- |
| Motivations (N=69) | **An opportunity to provide services to a key population** | **14** |
|  | * Household contacts will not otherwise go to a health facility | 2 |
|  | * Strong link between TB and other conditions | 4 |
|  | * Unethical to only focus on TB | 1 |
|  | * Need to broaden focus from infectious diseases given epidemiological transition | 2 |
|  | **Additional screening is 'attractive' for communities (increases uptake of screening)** | **14** |
|  | * Depends on the additional condition being screened for | 1 |
|  | * Evidence on stigma / uptake is lacking | 1 |
|  | * Reduce stigma and discrimination | 7 |
|  | **People affected by tuberculosis have multiple health needs - holistic care is important** | **12** |
|  | **Improved tuberculosis outcomes** | **11** |
|  | **Gains in efficiency, synergies in funding and programming** | **6** |
|  | **Improved patient experience** | **5** |
|  | **Better support to caregivers** | **4** |
|  | **Increased trust in the health care system (among a vulnerable or disengaged population)** | **2** |
|  | **Prevent tuberculosis** | **5** |
| Concerns (N=23) | **Feasibility, including risk of overloading services** | **12** |
|  | **Loss of efficiency** | **4** |
|  | **Cost of programmes and resource limitations** | **4** |
|  | **Privacy and stigma** | **3** |
|  | **Not a priority for or the job of the tuberculosis programme** | **2** |

Table 13: Agreement with statements outlining the motivation, principles, risks and needs for evidence generation and funding of integrated services for people with tuberculosis, stratified by WHO region and professional group of the panellist (N=324)

|  |  | N | Overall (N=324) | WHO region | | | | | | Professional group | | | |
| --- | --- | --- | --- | --- | --- | --- | --- | --- | --- | --- | --- | --- | --- |
|  | Statement |  |  | Africa (N=122) | Americas (N=40) | Eastern Mediterranean (N=21) | Europe (N=62) | South-East Asia (N=26) | Western Pacific (N=53) | Healthcare workers (N=81) | Policy makers, public health professionals, M&E specialists (N=118) | Researchers (N=79) | TB survivors, people with chronic conditions and civil society advocates (N=43) |
| Motivations for integration | It is important to provide holistic, person-centred care for people with TB | 320 | 98.4 | 98.3 | 95.0 | 100.0 | 100.0 | 96.2 | 100.0 | 98.8 | 99.1 | 97.5 | 97.7 |
|  | Integrated care will improve TB treatment outcomes | 319 | 95.6 | 98.3 | 80.0 | 95.2 | 98.4 | 96.2 | 98.0 | 97.5 | 96.5 | 91.1 | 97.7 |
|  | Integrated care will improve the health & wellbeing of people with TB, during and after TB treatment completion | 318 | 97.8 | 99.2 | 92.5 | 100.0 | 98.4 | 96.2 | 98.0 | 98.8 | 99.1 | 94.9 | 97.6 |
|  | The TB treatment period offers an excellent opportunity for integrated care, given the long period of health care engagement | 319 | 95.3 | 96.7 | 97.5 | 100.0 | 95.1 | 92.3 | 90.2 | 96.3 | 95.6 | 94.9 | 93.0 |
| Principles of integration | People with TB should be routinely screened for relevant non-TB conditions | 321 | 99.4 | 100.0 | 100.0 | 100.0 | 100.0 | 92.3 | 100.0 | 98.8 | 100.0 | 98.7 | 100.0 |
|  | TB care providers should provide screening for relevant non-TB conditions | 315 | 97.1 | 97.4 | 92.5 | 95.0 | 98.4 | 96.2 | 100.0 | 100.0 | 96.4 | 93.6 | 100.0 |
|  | TB care providers should incorporate care for relevant non-TB conditions, during the period of TB treatment | 319 | 91.5 | 95.0 | 82.1 | 85.7 | 88.7 | 92.3 | 96.1 | 93.8 | 93.8 | 86.1 | 93.0 |
| Risks of integration | Integrating screening for relevant non-TB conditions into TB care could reduce the quality of TB care | 316 | 25.6 | 29.7 | 34.2 | 23.8 | 19.4 | 30.8 | 15.7 | 27.3 | 21.6 | 24.4 | 35.7 |
|  | Integrating provision of relevant non-TB services into TB care could reduce the quality of TB care | 312 | 26.0 | 27.8 | 34.2 | 28.6 | 23.0 | 30.8 | 15.7 | 28.9 | 23.5 | 24.7 | 29.3 |
| Evidence and funding | Currently, there is not enough evidence for integration of non-TB screening and non-TB services as part of routine TB care for people receiving TB treatment to make policy & guideline recommendations | 291 | 54.6 | 61.8 | 40.0 | 55.6 | 30.9 | 64.0 | 70.8 | 63.8 | 51.5 | 45.9 | 61.9 |
|  | Research measuring the selection, effectiveness and feasibility of offering non-TB services as part of routine TB care for people receiving TB treatment should be prioritised | 289 | 92.4 | 95.4 | 80.6 | 94.4 | 96.2 | 84.0 | 93.8 | 95.7 | 94.1 | 88.0 | 90.2 |
|  | Domestic healthcare funding should support non-TB screening amongst people receiving TB treatment | 295 | 89.5 | 92.0 | 83.8 | 100.0 | 87.3 | 80.0 | 91.7 | 94.4 | 94.2 | 76.0 | 92.9 |
|  | Domestic healthcare funding should support provision of care for non-TB services amongst people receiving TB treatment | 295 | 86.4 | 88.4 | 81.1 | 94.4 | 85.5 | 80.0 | 87.5 | 90.1 | 91.3 | 72.0 | 92.9 |
|  | Among people receiving TB treatment, International TB organisations should provide support for screening for relevant non-TB conditions | 296 | 86.1 | 92.0 | 70.3 | 94.4 | 83.6 | 80.0 | 87.5 | 94.4 | 83.8 | 82.7 | 85.7 |
|  | Among people receiving TB treatment, International TB organisations should provide support for provision of relevant non-TB services | 292 | 81.8 | 87.2 | 70.3 | 94.4 | 74.5 | 76.0 | 85.4 | 90.1 | 80.8 | 77.0 | 80.0 |

**Footnotes**: Numbers shown indicate the percentage of all panellists responding to the statement indicated (for which the total shown as ‘N’) with either “Strongly agree” or “Somewhat agree”. Cell colours indicate whether consensus was reached (darker blue) or whether there was some (≥50%; lighter blue) or no agreement (white). Three people are not included in the ‘professional group’ as they could not be categorised into one of the four categories shown. **Abbreviations**: M&E = monitoring and evaluation; TB = tuberculosis.

Table 14: Percentage of panellists selecting an ambivalent response for statements about the motivation, principles, risks and needs for evidence generation and funding of integrated care for people with tuberculosis, stratified by WHO region and professional group of the panellist (N=324)

|  |  | N | WHO region | | | | | | | Professional group | | | | |
| --- | --- | --- | --- | --- | --- | --- | --- | --- | --- | --- | --- | --- | --- | --- |
|  | Statement |  | Africa (N=122) | Americas (N=40) | Eastern Mediterranean (N=21) | Europe (N=62) | South-East Asia (N=26) | Western Pacific (N=53) | Healthcare workers (N=81) | | Policy makers, public health professionals, M&E specialists (N=118) | Researchers (N=79) | TB survivors, people with chronic conditions and civil society advocates (N=43) | Africa (N=122) |
| Motivations for integration | It is important to provide holistic, person-centred care for people with TB | 320 | 0.9 | 1.7 | 2.5 | 0.0 | 0.0 | 0.0 | 0.0 | | 1.2 | 0.0 | 1.3 | 2.3 |
|  | Integrated care will improve TB treatment outcomes | 319 | 4.1 | 1.7 | 20.0 | 4.8 | 1.6 | 0.0 | 2.0 | | 2.5 | 3.5 | 7.6 | 2.3 |
|  | Integrated care will improve the health & wellbeing of people with TB, during and after TB treatment completion | 318 | 1.9 | 0.8 | 7.5 | 0.0 | 1.6 | 0.0 | 2.0 | | 1.2 | 0.9 | 3.8 | 2.4 |
|  | The TB treatment period offers an excellent opportunity for integrated care, given the long period of health care engagement | 319 | 3.1 | 2.5 | 2.5 | 0.0 | 3.3 | 3.8 | 5.9 | | 3.7 | 4.4 | 0.0 | 4.7 |
| Principles of integration | People with TB should be routinely screened for relevant non-TB conditions | 321 | 0.3 | 0.0 | 0.0 | 0.0 | 0.0 | 3.8 | 0.0 | | 1.2 | 0.0 | 0.0 | 0.0 |
|  | TB care providers should provide screening for relevant non-TB conditions | 315 | 2.2 | 2.6 | 7.5 | 0.0 | 1.6 | 0.0 | 0.0 | | 0.0 | 2.7 | 5.1 | 0.0 |
|  | TB care providers should incorporate care for relevant non-TB conditions, during the period of TB treatment | 319 | 6.9 | 5.0 | 17.9 | 4.8 | 9.7 | 0.0 | 3.9 | | 4.9 | 5.3 | 11.4 | 7.0 |
| Risks of integration | Integrating screening for relevant non-TB conditions into TB care could reduce the quality of TB care | 316 | 9.8 | 7.6 | 10.5 | 4.8 | 12.9 | 15.4 | 9.8 | | 6.5 | 10.3 | 14.1 | 7.1 |
|  | Integrating provision of relevant non-TB services into TB care could reduce the quality of TB care | 312 | 9.3 | 6.1 | 10.5 | 4.8 | 13.1 | 11.5 | 11.8 | | 6.6 | 8.7 | 11.7 | 12.2 |
| Evidence and funding | Currently, there is not enough evidence for integration of non-TB screening and non-TB services as part of routine TB care for people receiving TB treatment to make policy & guideline recommendations | 291 | 15.5 | 11.8 | 17.1 | 5.6 | 30.9 | 8.0 | 12.5 | | 10.1 | 14.6 | 24.3 | 11.9 |
|  | Research measuring the selection, effectiveness and feasibility of offering non-TB services as part of routine TB care for people receiving TB treatment should be prioritised | 289 | 4.8 | 2.8 | 13.9 | 5.6 | 3.8 | 4.0 | 4.2 | | 4.3 | 3.0 | 6.7 | 7.3 |
|  | Domestic healthcare funding should support non-TB screening amongst people receiving TB treatment | 295 | 8.8 | 6.2 | 16.2 | 0.0 | 12.7 | 12.0 | 6.2 | | 4.2 | 3.8 | 21.3 | 7.1 |
|  | Domestic healthcare funding should support provision of care for non-TB services amongst people receiving TB treatment | 295 | 11.5 | 9.8 | 18.9 | 5.6 | 14.5 | 12.0 | 8.3 | | 8.5 | 6.7 | 24.0 | 7.1 |
|  | Among people receiving TB treatment, International TB organisations should provide support for screening for relevant non-TB conditions | 296 | 9.5 | 4.4 | 24.3 | 5.6 | 14.5 | 12.0 | 4.2 | | 4.2 | 9.5 | 12.0 | 11.9 |
|  | Among people receiving TB treatment, International TB organisations should provide support for provision of relevant non-TB services | 292 | 13.0 | 8.3 | 24.3 | 5.6 | 21.8 | 16.0 | 6.2 | | 7.0 | 11.5 | 16.2 | 20.0 |

**Footnotes**: Numbers shown indicate the percentage of all panellists responding ‘neither agree nor disagree’ to the statement indicated (for which the total shown as ‘N’). Cell colours indicate whether ≥20% of respondents gave an ambivalent response (dark grey); 10-20% (light grey) or ≤10% did so (white). Three people are not included in the ‘professional group’ as they could not be categorised into one of the four categories shown. **Abbreviations**: M&E = monitoring and evaluation; TB = tuberculosis

Table 15: Agreement with statements outlining the motivations, principles, risks and need for evidence generation and funding of integrated services for people with tuberculosis, giving equal weighting to respondents from each WHO region, country or professional group (N=324)

| Category | Statement | Overall* | WHO region | Country | Profession* |
| --- | --- | --- | --- | --- | --- |
| Motivations for integration | It is important to provide holistic, person-centred care for people with TB | 98.4 | 98.3 | 99.5 | 98.6 |
|  | Integrated care will improve TB treatment outcomes | 95.6 | 94.4 | 97.5 | 96.6 |
|  | Integrated care will improve the health & wellbeing of people with TB, during and after TB treatment completion | 97.8 | 97.4 | 99.0 | 98.1 |
|  | The TB treatment period offers an excellent opportunity for integrated care, given the long period of health care engagement | 95.3 | 95.3 | 97.8 | 96.0 |
| Principles of integration | People with TB should be routinely screened for relevant non-TB conditions | 99.4 | 98.7 | 99.6 | 99.5 |
|  | TB care providers should provide screening for relevant non-TB conditions | 97.1 | 96.6 | 95.2 | 98.0 |
|  | TB care providers should incorporate care for relevant non-TB conditions, during the period of TB treatment | 91.5 | 90.0 | 91.5 | 86.7 |
| Risks of integration | Integrating screening for relevant non-TB conditions into TB care could reduce the quality of TB care | 25.6 | 25.6 | 23.4 | 28.4 |
|  | Integrating provision of relevant non-TB services into TB care could reduce the quality of TB care | 26.0 | 26.7 | 25.5 | 27.9 |
| Evidence and funding | Currently, there is not enough evidence for integration of non-TB screening and non-TB services as part of routine TB care for people receiving TB treatment to make policy & guideline recommendations | 54.6 | 53.9 | 57.4 | 57.9 |
|  | Research measuring the selection, effectiveness and feasibility of offering non-TB services as part of routine TB care for people receiving TB treatment should be prioritised | 92.4 | 90.7 | 90.8 | 93.6 |
|  | Domestic healthcare funding should support non-TB screening amongst people receiving TB treatment | 89.5 | 89.1 | 89.5 | 91.5 |
|  | Domestic healthcare funding should support provision of care for non-TB services amongst people receiving TB treatment | 86.4 | 86.1 | 87.5 | 89.3 |
|  | Among people receiving TB treatment, International TB organisations should provide support for screening for relevant non-TB conditions | 86.1 | 84.6 | 88.2 | 82.6 |
|  | Among people receiving TB treatment, International TB organisations should provide support for provision of relevant non-TB services | 81.8 | 81.3 | 84.0 | 78.9 |

**Footnotes**: Numbers shown indicate the percentage of all panellists responding to the statement indicated with either “Strongly agree” or “Somewhat agree”. Cell colours indicate whether consensus was reached (darker blue) or whether there was some (≥50%; lighter blue) or no agreement (white). The ‘Overall’ column is the overall, unweighted agreement; the other columns give equal weight to each category of the variable shown. For analysis of professional group, three panellists were excluded as they could not be classified into one of the four main professional groups considered. **Abbreviations**: TB = tuberculosis, WHO = World Health Organization.

Table 16: Themes relating to the principles of integrated tuberculosis screening and care, from free text comments (survey round 2)

| Principles | N = 141 |
| --- | --- |
| **Ensuring people affected by tuberculosis have access to care is important** | **36** |
| * Tuberculosis providers should screen but not necessarily provide care; care should be provided by the most appropriate person | 29 |
| * People can be referred for screening for non-tuberculosis conditions by other providers | 7 |
| * One stop shop model is preferable | 3 |
| **Burden of meeting the non-tuberculosis needs of people affected by tuberculosis should not wholly fall on tuberculosis care providers** | **2** |
| **Whether, who or how services delivered is context dependent, and will vary by condition or service** | **37** |
| * Need to consider feasibility (time and resources available) | 13 |
| * Will likely need an increase in funding or additional resource | 10 |
| * Need to consider whether services are available (do not screen if it cannot be managed) | 10 |
| ** If services are not otherwise available, they should be provided as part of tuberculosis care | 1 |
| ** Only care that can be delivered within tuberculosis treatment should be integrated | 1 |
| * Need to consider cost to people affected by tuberculosis | 3 |
| * Integrated care is currently too ambitious in some contexts | 3 |
| * People with tuberculosis should be prioritised over household contacts where needed | 2 |
| **Need to ensure quality of tuberculosis screening or care is not compromised** | **1** |
| **People affected by tuberculosis (people with tuberculosis and household contacts) need access to integrated primary health care and universal health coverage** | **21** |
| * Services should not be duplicated (services for people with tuberculosis integrated into the existing care system) | 7 |
| * 'Bidirectional' integration is more efficient | 5 |
| * Integrated services for household contacts should be part of broader community-based health services | 2 |
| **Development of services needs to be led by collaborative government initiatives** | **9** |
| * Needs to be part of country-level strategies & supported by guidelines | 2 |
| **Development of services should be informed by the community and their preferences** | **6** |
| * Consider burden placed on people by additional tests | 3 |
| **Integrated screening and care should be evidence-based** | **6** |
| * Currently this evidence does not exist | 2 |
| * The usual principles of screening apply | 2 |
| **Continuation of care after tuberculosis treatment is critical** | **5** |
| **Household contact services should be at the point of contact** | **3** |
| **Services should be available but autonomy respected (unless immediate public health concern)** | **3** |
| **People with tuberculosis and household contacts should receive counselling and health education (even when services are not available)** | **3** |
| **Screening requires a validated test** | **2** |

Table 17: Themes relating to the risks of integration for quality of tuberculosis screening and care, stratified by whether the panellist agreed that such risks existed (survey round 2)

|  | People with TB (N=74) | | | Household contacts (=48) | | |
| --- | --- | --- | --- | --- | --- | --- |
|  | Agree | Ambivalent | Disagree | Agree | Ambivalent | Disagree |
| ***N panellists leaving comments*** | ***23*** | ***11*** | ***40*** | ***22*** | ***8*** | ***18*** |
| **No, in fact integration will improve quality of care (thus improving TB outcomes or decreasing tuberculosis incidence)** | **1*** | **1** | **14** | **0** | **0** | **3** |
| **Integration will not impact on tuberculosis care quality** | **1*** | **0** | **3** | **0** | **0** | **0** |
| **Yes, there are risks to quality of tuberculosis screening and care, these include:** | **4** | **3** | **1** | **5** | **3** | **1** |
| * Burden placed on people with tuberculosis and caregivers | 1 | 1 | 0 | 0 | 1 | 0 |
| * Consequences of poorly evidenced screening | 0 | 0 | 0 | 1 | 0 | 1 |
| * Could deter people from attending health facilities or participating in screening | 0 | 2 | 1 | 2 | 2 | 0 |
| * Loss of focus (at programmatic or healthcare worker level) or too broad a scope to be achievable | 2 | 0 | 0 | 2 | 0 | 0 |
| **Risks exist if feasibility and resource concerns are not addressed** | **16** | **7** | **16** | **12** | **4** | **5** |
| **Risk that quality of care for non-TB conditions (provided as part of tuberculosis treatment) will be reduced** | **0** | **0** | **2** | **0** | **0** | **1** |

**Footnotes:** Responses are presented in aggregate, since most panellists gave concordant responses to the two questions about risks of integrated screening and care. Panellists were assigned to an agreement category if they provided the relevant response to either of the questions. * Review of these two individual responses suggests that these two panellists intended to agree with the statement posed.

Table 18: Themes on evidence generation and funding for integrated screening and care (both people with tuberculosis and household contacts; round 2 survey)

|  | Theme | N = 85 |
| --- | --- | --- |
| Evidence | **Action should not be contingent on more evidence - it already exists**** | **11** |
|  | **More evidence is needed to guide implementation** | **13** |
|  | * Need evidence on how integrated screening influence engagement (inc stigma) | 3 |
|  | * Need evidence on costs (inc considering opportunity cost) | 4 |
|  | * Need evidence on effectiveness (including specifically tuberculosis outcomes) | 5 |
|  | * Need evidence on impacts on health and wellbeing after tuberculosis | 2 |
|  | **Evidence generation - prioritised over what?** | **4** |
| Funding | **Additional funding is needed to provide integrated screening or care** | **10** |
|  | * Currently insufficient funding for non-TB services (NCDs, mental health, nutrition) generally | 3 |
|  | * Lack of attention on comorbidities among people with tuberculosis by international funders is hard to understand | 2 |
|  | * Need for investment in tuberculosis prevention | 3 |
|  | * Providing integrated care should not detract from 'TB specific' funding | 2 |
|  | **Funding priorities depend on context, integrated care may not be a top priority for some settings** | **4** |
|  | **International funding should be made available for evidence generation (inc operational research)** | **3** |
|  | **International funding is needed to implement integrated services, but long-term domestically funded programmes should be the goal** | **37** |
|  | * Approach to funding will depend on country context; external support particularly needed in high-TB burden countries | 8 |
|  | * Extent of support provided should depend on the non-TB condition | 2 |
|  | * Donors should collaborate and provide joint funding programmes across disease areas | 1 |
|  | * Providing holistic / integrated care is unlikely to get started without donor or external funding support | 3 |
|  | * Donor funding for integrated care should be as investment in PHC | 5 |
|  | * Programmes should be locally led (supporting sustainability) and implemented through existing country level systems | 13 |
|  | * Funding for this should be through funding of wider PHC strengthening | 1 |

**Footnotes**: Number (N) of panellists providing a response coded to this theme. Where responses were coded to multiple themes, individuals may appear more than once in the table. ** Of those who said more evidence is not needed, panellists provided examples of evidence for integration in the context of both people with tuberculosis and household contacts. **Abbreviations**: PHC = primary health care.

Table 19: Illustrative quotes from comments provided for statement “Household contacts of people treated for tuberculosis should be routinely screened for relevant non-tuberculosis conditions”, among people who disagreed with this statement (survey round 2; n=21/48 provided comments)

| “This will create workload for TB clinic. Instead, TB clinic should refer for screening at relevant non-TB condition clinics with specific specialties and then start the treatment accordingly.” *Woman, Nurse, SE Asia* |
| --- |
| “Only if TBI or TB disease is being diagnosed among the HHCs” *Man, Researcher, Europe* |
| “This depends on the burden on the programme and health system. Counselling the contacts and supporting them when they require information on health may be prudent, especially on TB risk factors and comorbidities, and those affecting the index TB patient themselves\|” *Man, Public health professional & Researcher, SE Asia* |
| “The scope of providing screening and care for non-TB conditions would be very logistically difficult.” *Woman, Doctor, Western Pacific* |
| “In our context this is a step too far. We can't even get health workers to go to households and screen the contacts of MDR/RR-TB patients for TB!” *Woman, Researcher, Africa* |
| “Funding is not adequate to allow screening programme staff to do this- time is already severely constrained” *Woman, Public Health Professional, SE Asia* |
| “We have to be very careful with screening to household members, particularly in the context of high stigma and discrimination.” *Man, Researcher, SE Asia* |
| “Really hard to answer in the absence of data on the prevalence of these conditions amongst household members, acceptability/feasibility of screening, and importantly information on if and how people could be linked to ongoing care for these conditions (many of which might be chronic diseases). Woudl be hesitant to mandate for screening / treatment during TB treatment of the index case, without ensuring separate longterm access to ongoing care.” *Woman, Doctor & Researcher, Europe* |

**Footnotes**: TBI = Mtb infection; TB = tuberculosis; HHCs = household contacts.

Table 20: Agreement with statements outlining the motivations, principles, risks and need for evidence generation and funding of integrated services for tuberculosis household contacts, stratified by WHO region and professional group of the panellist (N=324)

|  |  | N | Overall (N=324) | WHO region | | | | | | Professional group | | | | |
| --- | --- | --- | --- | --- | --- | --- | --- | --- | --- | --- | --- | --- | --- | --- |
|  | Statement |  |  | Africa (N=122) | Americas (N=40) | Eastern Mediterranean (N=21) | Europe (N=62) | South-East Asia (N=26) | Western Pacific (N=53) | | Healthcare workers (N=81) | Policy makers, public health professionals, M&E specialists (N=118) | Researchers (N=79) | TB survivors, people with chronic conditions and civil society advocates (N=43) |
| Motivations for integration | Holistic, person-centred care is important for TB household contacts | 301 | 89.4 | 94.7 | 74.4 | 95.0 | 87.5 | 91.3 | 87.8 | | 89.3 | 90.5 | 87.0 | 90.2 |
|  | Integrated relevant non-TB and TB screening for TB household contacts will improve participation in TB screening | 297 | 87.9 | 93.8 | 76.9 | 94.7 | 81.5 | 82.6 | 89.8 | | 88.0 | 91.1 | 80.5 | 92.7 |
|  | Integrated relevant non-TB and TB screening for TB household contacts will improve uptake and completion of TB preventive therapy | 298 | 83.9 | 88.5 | 68.4 | 100.0 | 78.6 | 82.6 | 85.7 | | 87.8 | 87.4 | 68.8 | 95.1 |
|  | The interaction with the TB affected household during TB screening is a good opportunity to provide non-TB screening to TB household contacts | 298 | 90.9 | 93.8 | 92.1 | 95.0 | 85.5 | 91.3 | 87.8 | | 92.0 | 92.2 | 84.4 | 97.6 |
|  | Offering TB screening that incorporates relevant non-TB screening and care will improve the health & wellbeing of TB household contacts | 298 | 91.9 | 97.3 | 78.9 | 100.0 | 87.3 | 82.6 | 95.9 | | 92.0 | 93.1 | 88.3 | 95.1 |
| Principles of integration | TB household contacts should be routinely screened for relevant non-TB conditions | 303 | 69.3 | 74.6 | 59.0 | 65.0 | 69.6 | 60.0 | 71.4 | | 67.1 | 71.4 | 63.6 | 76.2 |
|  | TB care providers should provide screening for relevant non-TB conditions to TB household contacts | 305 | 69.8 | 75.0 | 53.8 | 70.0 | 71.4 | 68.0 | 69.4 | | 67.5 | 76.4 | 57.1 | 78.6 |
|  | TB care providers should incorporate care for relevant non-TB conditions for TB household contacts, during the period that the household member with TB is receiving TB treatment | 301 | 64.1 | 71.4 | 48.7 | 65.0 | 55.4 | 60.0 | 71.4 | | 61.8 | 71.2 | 48.1 | 78.0 |
| Risks of integration | Integrating screening for relevant (non-TB) conditions for TB household contacts could reduce the quality of TB screening and delivery of TB preventive therapy (if relevant) | 300 | 35.7 | 36.6 | 42.1 | 35.0 | 25.0 | 52.0 | 32.7 | | 41.1 | 29.2 | 33.8 | 46.3 |
|  | Integrating provision of relevant (non-TB) services for TB household contacts could reduce the quality of TB screening and delivery of TB preventive therapy (if relevant) | 301 | 38.5 | 39.3 | 44.7 | 45.0 | 29.8 | 52.0 | 32.7 | | 45.3 | 31.1 | 38.2 | 46.3 |
| Evidence and funding | Currently, there is not enough evidence for integration of non-TB screening and non-TB services during systematic screening of household contacts to make policy & guideline recommendations | 290 | 60.0 | 61.5 | 55.6 | 61.1 | 38.9 | 72.0 | 77.1 | | 66.7 | 56.9 | 56.8 | 64.3 |
|  | Research measuring the selection, effectiveness and feasibility of offering non-TB services as part of systematic TB screening for TB household contacts should be prioritised | 291 | 84.5 | 91.7 | 64.9 | 88.9 | 77.8 | 84.0 | 89.6 | | 88.6 | 88.2 | 70.7 | 92.7 |
|  | Among people undergoing TB screening, international TB funding organisations should provide financial support for screening for non-TB conditions | 295 | 82.4 | 86.7 | 59.5 | 88.9 | 81.8 | 75.0 | 91.7 | | 91.5 | 81.0 | 70.7 | 92.7 |
|  | Among people undergoing TB screening, international TB funding organisations should provide support for provision of relevant non-TB services | 291 | 77.3 | 78.0 | 67.6 | 83.3 | 72.7 | 75.0 | 87.5 | | 84.5 | 78.6 | 64.4 | 85.4 |

**Footnotes**: Numbers shown indicate the percentage of all panellists responding to the statement indicated (for which the total shown as ‘N’) with either “Strongly agree” or “Somewhat agree”. Cell colours indicate whether consensus was reached (darker blue) or whether there was some (≥50%; lighter blue) or no agreement (white). Three people are not included in the ‘professional group’ as they could not be categorised into one of the four categories shown. **Abbreviations**: M&E = monitoring and evaluation; TB = tuberculosis

Table 21: Percentage of panellists selecting an ambivalent response for statements about motivation, principles, risks and needs for evidence generation and funding for integrated services for tuberculosis household contacts, stratified by WHO region and professional group of the panellist (N=324)

|  |  | N | Overall (N=324) | WHO region | | | | | | Professional group | | | |
| --- | --- | --- | --- | --- | --- | --- | --- | --- | --- | --- | --- | --- | --- |
| Category | Statement |  |  | Africa (N=122) | Americas (N=40) | Eastern Mediterranean (N=21) | Europe (N=62) | South-East Asia (N=26) | Western Pacific (N=53) | Healthcare workers (N=81) | Policy makers, public health professionals, M&E specialists (N=118) | Researchers (N=79) | TB survivors, people with chronic conditions and civil society advocates (N=43) |
| Motivations for integration | Holistic, person-centred care is important for TB household contacts | 301 | 7.0 | 1.8 | 23.1 | 5.0 | 7.1 | 0.0 | 10.2 | 8.0 | 6.7 | 7.8 | 4.9 |
|  | Integrated relevant non-TB and TB screening for TB household contacts will improve participation in TB screening | 297 | 9.8 | 4.4 | 23.1 | 5.3 | 14.8 | 13.0 | 6.1 | 9.3 | 6.9 | 15.6 | 7.3 |
|  | Integrated relevant non-TB and TB screening for TB household contacts will improve uptake and completion of TB preventive therapy | 298 | 12.1 | 8.8 | 26.3 | 0.0 | 17.9 | 4.3 | 10.2 | 9.5 | 8.7 | 23.4 | 4.9 |
|  | The interaction with the TB affected household during TB screening is a good opportunity to provide non-TB screening to TB household contacts | 298 | 6.0 | 3.5 | 7.9 | 5.0 | 9.1 | 4.3 | 8.2 | 5.3 | 4.9 | 10.4 | 2.4 |
|  | Offering TB screening that incorporates relevant non-TB screening and care will improve the health & wellbeing of TB household contacts | 298 | 5.7 | 0.0 | 21.1 | 0.0 | 9.1 | 8.7 | 4.1 | 5.3 | 4.9 | 7.8 | 4.9 |
| Principles of integration | TB household contacts should be routinely screened for relevant non-TB conditions | 303 | 14.5 | 14.0 | 7.7 | 25.0 | 16.1 | 8.0 | 18.4 | 13.2 | 14.3 | 19.5 | 9.5 |
|  | TB care providers should provide screening for relevant non-TB conditions to TB household contacts | 305 | 15.7 | 14.7 | 20.5 | 10.0 | 21.4 | 0.0 | 18.4 | 14.3 | 10.4 | 24.7 | 16.7 |
|  | TB care providers should incorporate care for relevant non-TB conditions for TB household contacts, during the period that the household member with TB is receiving TB treatment | 301 | 17.6 | 14.3 | 23.1 | 20.0 | 26.8 | 0.0 | 18.4 | 14.5 | 14.4 | 29.9 | 9.8 |
| Risks of integration | Integrating screening for relevant (non-TB) conditions for TB household contacts could reduce the quality of TB screening and delivery of TB preventive therapy (if relevant) | 300 | 13.3 | 10.7 | 18.4 | 10.0 | 21.4 | 12.0 | 8.2 | 12.3 | 12.3 | 19.5 | 7.3 |
|  | Integrating provision of relevant (non-TB) services for TB household contacts could reduce the quality of TB screening and delivery of TB preventive therapy (if relevant) | 301 | 12.0 | 9.8 | 15.8 | 5.0 | 17.5 | 12.0 | 10.2 | 10.7 | 11.3 | 17.1 | 7.3 |
| Evidence and funding | Currently, there is not enough evidence for integration of non-TB screening and non-TB services during systematic screening of household contacts to make policy & guideline recommendations | 290 | 20.0 | 16.5 | 16.7 | 16.7 | 40.7 | 4.0 | 16.7 | 17.4 | 16.7 | 28.4 | 16.7 |
|  | Research measuring the selection, effectiveness and feasibility of offering non-TB services as part of systematic TB screening for TB household contacts should be prioritised | 291 | 10.3 | 5.5 | 24.3 | 5.6 | 16.7 | 4.0 | 8.3 | 7.1 | 7.8 | 20.0 | 4.9 |
|  | Among people undergoing TB screening, international TB funding organisations should provide financial support for screening for non-TB conditions | 295 | 12.9 | 8.8 | 32.4 | 11.1 | 14.5 | 16.7 | 4.2 | 5.6 | 11.4 | 24.0 | 7.3 |
|  | Among people undergoing TB screening, international TB funding organisations should provide support for provision of relevant non-TB services | 291 | 16.5 | 16.5 | 27.0 | 11.1 | 21.8 | 16.7 | 4.2 | 9.9 | 11.7 | 30.1 | 14.6 |

**Footnotes**: Numbers shown indicate the percentage of all panellists responding ‘neither agree nor disagree’ to the statement indicated (for which the total shown as ‘N’). Cell colours indicate whether ≥20% of respondents gave an ambivalent response (dark grey); 10-20% (light grey) or ≤10% did so (white). **Abbreviations**: M&E = monitoring and evaluation; TB = tuberculosi

Table 22: Agreement with statements outlining the motivations, principles, risks and need for evidence generation and funding of integrated services for tuberculosis household contacts, giving equal weighting to respondents from each WHO region, country or professional group (N=324)

| Category | Statement | Overall* | WHO region | Country | Profession* |
| --- | --- | --- | --- | --- | --- |
| Motivations for integration | Holistic, person-centred care is important for TB household contacts | 89.4 | 88.4 | 90.7 | 91.4 |
|  | Integrated relevant non-TB and TB screening for TB household contacts will improve participation in TB screening | 87.9 | 86.6 | 86.3 | 90.5 |
|  | Integrated relevant non-TB and TB screening for TB household contacts will improve uptake and completion of TB preventive therapy | 83.9 | 84.0 | 85.2 | 87.8 |
|  | The interaction with the TB affected household during TB screening is a good opportunity to provide non-TB screening to TB household contacts | 90.9 | 90.9 | 91.4 | 93.2 |
|  | Offering TB screening that incorporates relevant non-TB screening and care will improve the health & wellbeing of TB household contacts | 91.9 | 90.3 | 91.2 | 93.7 |
| Principles of integration | TB household contacts should be routinely screened for relevant non-TB conditions | 69.3 | 66.6 | 72.9 | 75.7 |
|  | TB care providers should provide screening for relevant non-TB conditions to TB household contacts | 69.8 | 67.9 | 75.6 | 75.9 |
|  | TB care providers should incorporate care for relevant non-TB conditions for TB household contacts, during the period that the household member with TB is receiving TB treatment | 64.1 | 62.0 | 64.9 | 71.8 |
| Risks of integration | Integrating screening for relevant (non-TB) conditions for TB household contacts could reduce the quality of TB screening and delivery of TB preventive therapy (if relevant) | 35.7 | 37.2 | 29.3 | 36.8 |
|  | Integrating provision of relevant (non-TB) services for TB household contacts could reduce the quality of TB screening and delivery of TB preventive therapy (if relevant) | 38.5 | 40.6 | 32.9 | 38.9 |
| Evidence and funding | Currently, there is not enough evidence for integration of non-TB screening and non-TB services during systematic screening of household contacts to make policy & guideline recommendations | 60.0 | 61.0 | 57.7 | 55.6 |
|  | Research measuring the selection, effectiveness and feasibility of offering non-TB services as part of systematic TB screening for TB household contacts should be prioritised | 84.5 | 82.8 | 86.1 | 88.0 |
|  | Among people undergoing TB screening, international TB funding organisations should provide financial support for screening for non-TB conditions | 82.4 | 80.6 | 83.5 | 80.5 |
|  | Among people undergoing TB screening, international TB funding organisations should provide support for provision of relevant non-TB services | 77.3 | 77.4 | 83.5 | 75.9 |

**Footnotes**: Numbers shown indicate the percentage of all panellists responding to the statement indicated with either “Strongly agree” or “Somewhat agree”. Cell colours indicate whether consensus was reached (darker blue) or whether there was some (≥50%; lighter blue) or no agreement (white). The ‘Overall’ column is the overall, unweighted agreement; the other columns give equal weight to each category of the variable shown. For analysis of professional group, three panellists were excluded as they could not be classified into one of the four main professional groups considered. **Abbreviations**: TB = tuberculosis, WHO = World Health Organization.

### Considerations for developing integrated tuberculosis care or screening services

Table S22 summarises emergent themes relating to the necessary resources and infrastructure to deliver integrated tuberculosis screening and care. These span all the WHO health system building blocks. Many respondents emphasised the need to consider context in developing and implementing integrated health interventions – this included the epidemiological context (i.e. local disease prevalence), health system context (i.e. feasibility, affordability) and the social and cultural context (i.e. acceptability). A cross-cutting theme across the building blocks was the need for integration not only at the point of care, but for better communication and collaboration at multiple levels of the health system (e.g. across national and regional health departments, or across different facilities to support ongoing care); as well as the currently limited evidence available to support integrated screening. This lack of evidence translated to an identified need for trials to inform further development of policy and guidelines. Several panellists highlighted the need to be mindful, as integrated services are developed, to maximise inclusivity, ensuring reach extends to marginalized and often neglected groups including the elderly, people with disabilities, refugees, people who use drugs, and people who are in prison.

Key barriers to implementation were often the converse of the described requirements, with the most cited health system barriers being lack of financial (n=90/223 panellists), human (i.e. sufficient, trained, healthcare workers; n=82) and other resources (e.g. tests and equipment; n=31); lack of will for provision of additional services among healthcare providers (n=27); and lack of referral or other pathways towards ongoing care (n=26). The existence of siloed care, lacking mechanisms for collaboration and co-ordination were mentioned as a barrier by 23 panellists. Community barriers included stigma (n=31), lack of community awareness, engagement and mobilisation (n=31), low trust in the health system (n=22), distance to health facilities (n=13) and the costs that might be incurred by participants (n=19). Seventeen panellists cited the lack of clear guidance and polices to support integration as a barrier.

Table 23: Considerations for implementation of integrated tuberculosis screening and care, from thematic analysis of free text responses (round 1)

|  |  | Round 1 | |
| --- | --- | --- | --- |
| Building block | Theme | Requirement | Current barrier |
| Service delivery | Screening aids, management **guidelines**, IEC materials, SOPs, software and tools | 47 | 7 |
|  | Free, accessible and timely treatment programs/**referral services** available for conditions that are screened | 31 | 26 |
|  | Well-established, well-run community-based healthcare presence within geographic reach (**strong primary care**) | 23 | 13 |
|  | Development of more accessible, affordable **POC tests** for conditions | 14 |  |
|  | Reliable supply chains | 9 | 10 |
|  | Functioning, sufficiently resourced base **patient follow-up** and household contact tracing process | 8 |  |
|  | Appropriate community-based psychosocial care programs | 8 |  |
|  | **Evidence** of successful integration in similar setting | 3 | 20 |
| Health workforce | Staff **training** and mentorship | 53 |  |
|  | **Human resource** / availability of healthcare workers / sufficient clinician time | 55 | 82 |
|  | **Healthcare worker buy-in** and engagement with person-centred care | 7 | 27 |
| Health information systems | Well-linked **laboratory** services | 10 |  |
|  | Robust electronic medical record system | 7 | 6 |
|  | Monitoring and evaluation systems | 6 | 7 |
| Essential diagnostics and medicines | **Equipment** / diagnostics / test-kits for screening that are appropriate for use in the community | 48 | 31 |
| Financing | Adequate **financing** (healthcare workers / services) | 41 | 99 |
|  | Adequate support to overcome socio-economic barriers, including to attend additional appointments if needed | 7 | 19 |
| Leadership and governance | Close **collaboration** among health departments, non-governmental organisations, community organisations and other stakeholders | 14 | 23 |
|  | National **policies** & guidelines include support service integration | 13 | 17 |
|  | Political commitment | 7 | 13 |
|  | Strong leadership and governance of community-based / primary care services | 6 | 21 |
| Community engagement | Strong **community engagement,** sensitization and buy-in (including trust in health providers and services | 19 | 53 |
|  | Existence of tuberculosis stigma / need for stigma reduction |  | 31 |

Fig 3: Agreement with statements outlining the motivations, principles, risks and need for evidence generation and funding of integrated services as part of population-wide tuberculosis active case finding activities (N=324)

**Footnotes**: Data collected as checkboxes on a 5-point Likert scale. The y-axis represents the statements presented to panellists and the x-axis the cumulative proportion of panellists who somewhat agreed and strongly agreed with the statements (positive scale) or who somewhat disagreed and strongly disagreed (negative scale). Not all panellists provided a response for every statement: N is the total number of panellists and n (per statement) is the number of panellists providing a response to that statement. Responses that neither agreed nor disagreed with the statements are displayed separately (grey bars). Consensus was considered reached if ≥75% of panellists either agreed or disagreed (grey dotted line). **Abbreviations**: TB = tuberculosis.

Table 24: Agreement with statements outlining the motivations and principles of integrated population-wide tuberculosis active case finding, stratified by WHO region or professional group (N=324)

|  | N | Overall (N=324) | WHO region | | | | | | Professional group | | | |
| --- | --- | --- | --- | --- | --- | --- | --- | --- | --- | --- | --- | --- |
| Label |  |  | Africa (N=122) | Americas (N=40) | Eastern Mediterranean (N=21) | Europe (N=62) | South-East Asia (N=26) | Western Pacific (N=53) | Healthcare workers (N=81) | Policy makers, public health professionals, M&E specialists (N=118) | Researchers (N=79) | TB survivors, people with chronic conditions and civil society advocates (N=43) |
| Community-wide systematic TB screening programs are a good opportunity to screen for other conditions that are important to the community | 299 | 94.3 | 95.6 | 92.3 | 94.4 | 92.7 | 84.0 | 100.0 | 95.9 | 95.3 | 90.7 | 95.1 |
| People participating in community-wide systematic TB screening in high TB incidence settings should be offered screening for relevant non-TB conditions as part of the TB screening programme | 299 | 92.6 | 92.9 | 92.3 | 100.0 | 89.3 | 88.0 | 95.8 | 90.5 | 90.6 | 93.3 | 100.0 |
| Programmes screening for relevant non-TB conditions during community-wide systematic TB screening have a responsibility to ensure participants can access relevant non-TB care | 297 | 88.9 | 88.5 | 84.6 | 100.0 | 90.9 | 84.0 | 89.4 | 89.2 | 87.6 | 88.0 | 92.5 |

**Abbreviations**: TB = tuberculosis.

Table 25: Agreement with statements outlining the motivations and principles of integrated population-wide tuberculosis active case finding, giving equal weighting to respondents from each WHO region, country or professional group (N=324)

| Label | Overall* | WHO region | Country | Profession* |
| --- | --- | --- | --- | --- |
| Community-wide systematic TB screening programs are a good opportunity to screen for other conditions that are important to the community | 94.3 | 93.2 | 92.6 | 95.4 |
| People participating in community-wide systematic TB screening in high TB incidence settings should be offered screening for relevant non-TB conditions as part of the TB screening programme | 92.6 | 93.1 | 93.3 | 94.9 |
| Programmes screening for relevant non-TB conditions during community-wide systematic TB screening have a responsibility to ensure participants can access relevant non-TB care | 88.9 | 89.6 | 88.7 | 91.5 |

**Footnotes**: Numbers shown indicate the percentage of all panellists responding to the statement indicated with either “Strongly agree” or “Somewhat agree”. Cell colours indicate whether consensus was reached (darker blue) or whether there was some (≥50%; lighter blue) or no agreement (white). The ‘Overall’ column is the overall, unweighted agreement; the other columns give equal weight to each category of the variable shown. For analysis of professional group, three panellists were excluded as they could not be classified into one of the four main professional groups considered. **Abbreviations**: TB = tuberculosis, WHO = World Health Organization.

Table 26: Themes on integrated population-wide screening interventions, by panellist agreement that screening for non-tuberculosis conditions should be routinely included in such programmes (round 2 survey; n=49 people left comments)

|  |  | **Overall*** | Agree | Ambivalent | Disagree |
| --- | --- | --- | --- | --- | --- |
|  | **There is evidence to support this** | **3** | 2 | 0 | 1 |
| **Motivations** | **An opportunity to provide services to a key population** | **1** | 1 | 0 | 0 |
|  | **Additional screening is 'attractive' (increases uptake of screening)** | **5** | 4 | 0 | 1 |
|  | * Reduce stigma and discrimination | **2** | 2 | 0 | 0 |
|  | * Gains in efficiency, synergies in funding and programming | **3** | 3 | 0 | 0 |
|  | **Integrated, PHC based services are the goal** | **3** | 3 | 0 | 0 |
|  | **Opportunity for TB-sensitive health education** | **2** | 2 | 0 | 0 |
|  | **Increases impact** | **1** | 1 | 0 | 0 |
| **Principles** | **Ensuring people have access to care is important (e.g. through referral with or without support for linkage to care** | **10** | 9 | 0 | 1 |
|  | * Need to consider whether services are available | **5** | 4 | 0 | 1 |
|  | **Whether, who or how services are delivered is context dependent, and will vary by condition or service** | **8** | 7 | 1 | 0 |
|  | * Need to consider feasibility (time and resources available) | **5** | 5 | 0 | 0 |
|  | * Will likely need an increase in funding or additional resource (including to support care services – health systems strengthening) | **4** | 4 | 0 | 0 |
|  | **People affected by TB (PWTB, HHC, community) need access to integrated primary health care (UHC)** | **5** | 5 | 0 | 0 |
|  | * Need to be led by collaborative government-led initiatives | **3** | 3 | 0 | 0 |
|  | **Should be informed by the community / consider preferences of the community being served** | **3** | 1 | 2 | 0 |
|  | **Integrated screening and care should be evidence-based** | **2** | 1 | 0 | 1 |
|  | **Need to ensure TB screening or care is not compromised** | **4** | 3 | 0 | 1 |
|  | * Other conditions should only be included once TB screening is established | **1** | 1 | 0 | 0 |
| **Concerns** | Concerns about feasibility | **6** | 4 | 1 | 1 |
|  | Concerns about efficiency | **2** | 1 | 0 | 1 |
|  | Concerns about cost of programmes and resource limitations | **1** | 0 | 1 | 0 |
|  | Concerns about privacy | **1** | 0 | 1 | 0 |

**Footnotes**: * An additional two panellists left comments suggesting that population-wide screening for tuberculosis (TB) (or any other condition) should not be done due to concerns about underpinning evidence and cost-effectiveness.

Table 27: Agreement with statements outlining the key considerations for selection of conditions and services for integration into tuberculosis screening or care, stratified by WHO region or professional group (N=324)

|  |  | N | Overall (N=324) | WHO region | | | | | | Professional group | | | |
| --- | --- | --- | --- | --- | --- | --- | --- | --- | --- | --- | --- | --- | --- |
| Group | Label |  |  | Africa (N=122) | Americas (N=40) | Eastern Mediterranean (N=21) | Europe (N=62) | South-East Asia (N=26) | Western Pacific (N=53) | Healthcare workers (N=81) | Policy makers, public health professionals, M&E specialists (N=118) | Researchers (N=79) | TB survivors, people with chronic conditions and civil society advocates (N=43) |
| People with TB | For PWTB, the local disease burden (among the general population) | 317 | 90.9 | 95.0 | 92.3 | 81.0 | 89.7 | 73.1 | 94.2 | 88.6 | 93.0 | 91.1 | 92.9 |
|  | For PWTB, the association of the condition with TB risk or TB outcomes | 319 | 95.0 | 96.7 | 92.5 | 100.0 | 88.3 | 96.2 | 98.1 | 95.0 | 97.4 | 88.6 | 100.0 |
|  | The ability to provide screening using a simple, cheap and accurate test, with results available on the same day | 319 | 84.0 | 82.5 | 79.5 | 95.2 | 82.0 | 80.8 | 90.4 | 87.5 | 79.8 | 79.7 | 95.3 |
|  | The ability to provide affordable and effective treatment locally | 321 | 87.5 | 87.5 | 77.5 | 85.7 | 85.5 | 92.3 | 96.2 | 91.4 | 87.0 | 83.5 | 90.7 |
|  | The feasibility of integrating care for the condition with TB treatment, during the period of TB treatment | 317 | 85.8 | 83.9 | 80.0 | 85.7 | 85.0 | 92.3 | 92.3 | 82.1 | 91.2 | 79.7 | 90.7 |
|  | The feasibility of continuing/sustaining care beyond the period of TB treatment (if required) | 315 | 83.2 | 82.8 | 72.5 | 90.5 | 80.3 | 96.2 | 86.3 | 89.7 | 82.5 | 73.1 | 90.5 |
|  | Acceptability of screening and care for the non-TB condition in the community | 316 | 84.5 | 82.2 | 80.0 | 85.7 | 83.1 | 92.3 | 90.4 | 85.7 | 83.3 | 83.5 | 88.4 |
| TB screening | For HHC, the local disease burden (among the general population) | 305 | 86.6 | 92.2 | 84.6 | 95.0 | 84.2 | 68.0 | 83.7 | 84.0 | 88.9 | 89.6 | 81.0 |
|  | For HHC, the association of the condition with TB risk or TB outcomes | 303 | 87.1 | 94.7 | 74.4 | 90.0 | 78.6 | 80.0 | 91.8 | 89.2 | 89.7 | 77.9 | 92.9 |
|  | The availability to provide screening using a simple, cheap and accurate test, with results available on the same day | 302 | 81.1 | 81.4 | 71.8 | 100.0 | 76.8 | 80.0 | 85.7 | 80.0 | 83.0 | 75.3 | 92.7 |
|  | The availability of affordable and effective treatment locally | 302 | 82.5 | 83.3 | 73.7 | 100.0 | 75.4 | 84.0 | 87.5 | 82.7 | 83.2 | 77.6 | 90.2 |
|  | The feasibility of integrating care for the condition with TB preventive treatment (TPT), during the period of preventive TB treatment | 302 | 79.8 | 82.5 | 71.8 | 90.0 | 71.4 | 80.0 | 85.4 | 80.0 | 83.0 | 70.1 | 90.2 |
|  | The feasibility of continuing/sustaining care beyond the period of TB preventive treatment (if required) | 301 | 75.7 | 77.9 | 66.7 | 85.0 | 71.4 | 72.0 | 81.2 | 76.0 | 74.5 | 69.7 | 87.8 |
|  | Acceptability of screening and care for the condition amongst TB household contacts | 299 | 86.3 | 89.3 | 76.3 | 95.0 | 80.4 | 88.0 | 89.6 | 85.3 | 89.4 | 80.3 | 90.2 |

**Footnotes**: Numbers shown indicate the percentage of all panellists responding to the statement indicated (for which the total shown as ‘N’) with either “Strongly agree” or “Somewhat agree”. Cell colours indicate whether consensus was reached (darker blue) or whether there was some (≥50%; lighter blue) or no agreement (white). **Abbreviations**: PWTB = people with TB; TB = tuberculosis.

Table 28: Agreement with statements outlining the key considerations for selection of conditions and services for integration into tuberculosis screening or care, giving equal weighting to respondents from each WHO region, country or professional group (N=324)

| Group | Label | Overall* | WHO region | Country | Profession |
| --- | --- | --- | --- | --- | --- |
| People with TB | For PWTB, the local disease burden (among the general population) | 90.9 | 87.5 | 85.8 | 79.8 |
|  | For PWTB, the association of the condition with TB risk or TB outcomes | 95.0 | 95.3 | 96.1 | 96.2 |
|  | The ability to provide screening using a simple, cheap and accurate test, with results available on the same day | 84.0 | 85.1 | 86.4 | 88.5 |
|  | The ability to provide affordable and effective treatment locally | 87.5 | 87.4 | 85.1 | 83.8 |
|  | The feasibility of integrating care for the condition with TB treatment, during the period of TB treatment | 85.8 | 86.5 | 82.7 | 82.1 |
|  | The feasibility of continuing/sustaining care beyond the period of TB treatment (if required) | 83.2 | 84.7 | 86.2 | 87.2 |
|  | Acceptability of screening and care for the non-TB condition in the community | 84.5 | 85.6 | 84.0 | 81.5 |
| TB screening | For HHC, the local disease burden (among the general population) | 86.6 | 84.6 | 86.6 | 82.0 |
|  | For HHC, the association of the condition with TB risk or TB outcomes | 87.1 | 84.9 | 87.7 | 89.9 |
|  | The availability to provide screening using a simple, cheap and accurate test, with results available on the same day | 81.1 | 82.6 | 80.3 | 72.9 |
|  | The availability of affordable and effective treatment locally | 82.5 | 84.0 | 78.9 | 80.1 |
|  | The feasibility of integrating care for the condition with TB preventive treatment (TPT), during the period of preventive TB treatment | 79.8 | 80.2 | 80.0 | 78.0 |
|  | The feasibility of continuing/sustaining care beyond the period of TB preventive treatment (if required) | 75.7 | 75.7 | 76.5 | 81.6 |
|  | Acceptability of screening and care for the condition amongst TB household contacts | 86.3 | 86.4 | 86.7 | 89.1 |

**Footnotes**: Numbers shown indicate the percentage of all panellists responding to the statement indicated with either “Strongly agree” or “Somewhat agree”. Cell colours indicate whether consensus was reached (darker blue) or whether there was some (≥50%; lighter blue) or no agreement (white). The ‘Overall’ column is the overall, unweighted agreement; the other columns give equal weight to each category of the variable shown. For analysis of professional group, three panellists were excluded as they could not be classified into one of the four main professional groups considered. **Abbreviations**: TB = tuberculosis, WHO = World Health Organization.

## Current status of integrated tuberculosis screening and care

Table S27 summarises panellists’ reports of which services were currently available in individual country contexts with which they were familiar. Other services reported for people with tuberculosis were smoking, hepatitis C testing, pregnancy testing, silicosis screening, rehabilitation/disability assessment, asthma, alcohol use disorders, STI, harm reduction, vaccines, visual acuity screening, ECG (each n<5 mentions). Among household contacts, Hepatitis C testing, immunisations and support for travel to attend tuberculosis screening were mentioned (each n=1). The following paragraphs provide a narrative summary of the associated free-text data from this question.

When panellists were asked how services were currently offered for people with tuberculosis, this was either through co-location (mentioned in n=137 country-specific responses) or referral (n=68). Most co-located or ‘one stop shop services’ provided HIV testing with or without treatment; others included diabetes screening and nutritional/social support. HIV testing was usually reported as being well implemented and offered to all people with tuberculosis, whilst many other conditions (notably, mental health, nutritional and social support) were only considered among people with drug-resistant tuberculosis. Most diagnosis and treatment of conditions other than HIV was through referrals.

For tuberculosis household contacts, services were usually through referral (mentioned in n=35 country-specific responses) rather than co-located services such as a household visit (n=29). HIV testing was either offered to all household contacts (n=8) or offered if the index case has HIV (n=4). Several panellists highlighted that the services that were available for tuberculosis household contacts were not specific to this group; but formed part of community-based services (n=5). In 7 country-specific responses the panellist highlighted how HIV testing for tuberculosis household contacts was in guidelines or available ‘in theory’ but in practice was not.

Table 29: Services reported by participants as already being available in their setting (survey round 2)

| **Services** | **Individual panellists (N=375)** | | **Countries (N=80)*** | |
| --- | --- | --- | --- | --- |
|  | **People with tuberculosis** | **Household contacts** | **People with tuberculosis** | **Household contacts** |
| Diabetes screening | 155 (41.3%) | 46 (12.3%) | 23 (28.7%) | 9 (11.2%) |
| HIV screening | 337 (89.9%) | 135 (36%) | 71 (88.8%) | 20 (25%) |
| Hepatitis B screening | 94 (25.1%) | 36 (9.6%) | 16 (20%) | 10 (12.5%) |
| Hypertension screening | 92 (24.5%) | 40 (10.7%) | 14 (17.5%) | 7 (8.8%) |
| Mental health support | 99 (26.4%) | 52 (13.9%) | 13 (16.2%) | 11 (13.8%) |
| Nutritional support | 158 (42.1%) | 51 (13.6%) | 25 (31.2%) | 5 (6.2%) |
| Social protection | 105 (28%) | 51 (13.6%) | 14 (17.5%) | 9 (11.2%) |

**Footnotes**: N panellists is the total number of individual country settings reported by panellists (each panellist could report for more than one country setting). N countries is the number of individual countries on which at least one panellist reported. For determining whether a service was available in country-level analysis, we considered it to be offered if at least 67% panellists reported that it was available in that country.

Fig 4: Ranking of the most important conditions and services to be included for people with tuberculosis and household contacts (n=375 responses for 80 country settings)

**Footnotes** Conditions are ordered by their ranking across all country-specific scenarios provided. Percentages shown are the percentages of individual country settings in which panellists agreed this condition should be included in integrated screening or care. Solid borders and bold font indicate where the condition was ranked by panellists in >75% of country settings. Colours indicate the ‘category’ of the condition: yellow = infectious diseases, blue = non-communicable diseases, green = mental health and purple = substance use disorders.

Table 30: Number of times conditions to be included as part of integrated care for people with TB and household contacts were ranked in first, second and third place, and total number of rankings received (n=375 rankings for 80 countries; survey round 2)

| **Services** | **People with TB** | | | |  | **Household contacts** | | | |
| --- | --- | --- | --- | --- | --- | --- | --- | --- | --- |
|  | **1st** | **2nd** | **3rd** | **Total** |  | **1st** | **2nd** | **3rd** | **Total** |
| HIV | 267 | 27 | 12 | 349 |  | 198 | 31 | 24 | 301 |
| Diabetes | 24 | 123 | 42 | 337 |  | 22 | 85 | 34 | 263 |
| Nutrition | 23 | 89 | 78 | 346 |  | 36 | 81 | 50 | 284 |
| Financial hardship | 18 | 18 | 44 | 320 |  | 30 | 22 | 43 | 255 |
| Mental health | 11 | 42 | 63 | 326 |  | 19 | 41 | 52 | 248 |
| Chronic lung disease | 10 | 28 | 38 | 310 |  | 11 | 26 | 20 | 228 |
| Smoking | 3 | 12 | 34 | 319 |  | 11 | 11 | 30 | 247 |
| Alcohol use disorder | 1 | 4 | 12 | 307 |  | 0 | 8 | 14 | 227 |
| Hypertension | 1 | 7 | 13 | 290 |  | 2 | 11 | 21 | 226 |
| Cardiovascular disease | 0 | 1 | 6 | 292 |  | 2 | 1 | 6 | 205 |
| Substance use | 0 | 5 | 6 | 288 |  | 0 | 6 | 9 | 209 |

**Footnotes:** Panellists were asked to leave a condition blank if they did not believe it was important. The number of people who did not rank a condition is therefore the difference between the total votes received and the total number of country settings entered (n=375). **Abbreviations**: CV disease = cardiovascular disease; TB = tuberculosis.

Fig 5: Ranking of conditions stratified by WHO world region (N=375 individual country scenarios)

1. **Among people with TB**

1. **Among household contacts**

**Footnotes** Conditions are ordered by their ranking across all country-specific scenarios provided within the WHO region shown. Percentages shown are the percentages of individual country settings in which panellists agreed this condition should be included in integrated screening or care. Solid borders and bold font indicate where the condition was ranked by panellists in >75% of country settings Colours indicate the ‘category’ of the condition: yellow = infectious diseases, blue = non-communicable diseases, green = mental health and purple = substance use disorders.

Fig 6: Ranking of conditions using weighted average ranking across strata of WHO world region (N=375 individual country scenarios)

**Footnotes** Conditions are ordered by their ranking across all country-specific scenarios provided, giving equal weight to responses from each WHO region. Percentages shown are the percentages of individual country settings in which panellists agreed this condition should be included in integrated screening or care, giving equal weighting to each WHO region. Solid borders and bold font indicate where the condition was ranked by panellists in >75% of country settings. Colours indicate the ‘category’ of the condition: yellow = infectious diseases, blue = non-communicable diseases, green = mental health and purple = substance use disorders.

Fig 7: Ranking of conditions stratified by professional group (N=375 individual country scenarios)

1. **Among people with TB**

1. **Among household contacts**

**Footnotes** Conditions are ordered by their ranking across all country-specific scenarios provided by people of the professional group shown. Percentages shown are the percentages of individual country settings in which panellists agreed this condition should be included in integrated screening or care. Solid borders and bold font indicate where the condition was ranked by panellists in >75% of country settings. Colours indicate the ‘category’ of the condition: yellow = infectious diseases, blue = non-communicable diseases, green = mental health and purple = substance use disorders.

Fig 8: Ranking of conditions using weighted average ranking across strata of professional group (N=375 individual country scenarios)

**Footnotes** Conditions are ordered by their ranking across all country-specific scenarios provided, giving equal weight to responses from each professional group. Percentages shown are the percentages of individual country settings in which panellists agreed this condition should be included in integrated screening or care, giving equal weighting to each professional group. Solid borders and bold font indicate where the condition was ranked by panellists in >75% of country settings. Colours indicate the ‘category’ of the condition: yellow = infectious diseases, blue = non-communicable diseases, green = mental health and purple = substance use disorders.

Table 31: Core list of consensus statements on integrated screening and care for people with tuberculosis and their household contacts

| Population /Topic | Consensus statements |
| --- | --- |
| Among people with tuberculosis | 1. It is important to provide holistic person-centred care for people with tuberculosis.  2. Integrated care for people with tuberculosis will improve tuberculosis treatment outcomes.  3. Integrated care for people with tuberculosis will improve the health and wellbeing of people with tuberculosis, during and after tuberculosis treatment completion.  4. The tuberculosis treatment period offers an excellent opportunity for integrated care, given the long period of healthcare engagement.  5. People with tuberculosis should be routinely screened for relevant non-tuberculosis conditions.  6. Tuberculosis care providers should provide screening for relevant non-tuberculosis conditions.  7. Tuberculosis care providers should incorporate care for relevant non-tuberculosis conditions, during the period of tuberculosis treatment.  8. The definition of relevant non-tuberculosis condition should be determined by a) the local disease burden (among the general population), b) the association of the conditions with tuberculosis risk or tuberculosis outcomes, c) the ability to provide screening using a simple, cheap and accurate test, with results available on the same day, d) the ability to provide affordable and effective treatment locally, e) the feasibility of integrating care for the condition with tuberculosis treatment, during the period of tuberculosis treatment, f) the feasibility of continuing/sustaining care beyond the period of tuberculosis treatment if required and g) the acceptability of screening and care for the non-tuberculosis condition in the community.  9. Conditions screened/treated among for people with tuberculosis should include HIV, diabetes, nutrition, mental health disorders, financial hardship, smoking, chronic lung disease, alcohol use disorders, cardiovascular disease, hypertension and substance use. |
| Among tuberculosis household contacts | 1. Holistic person-centred care is important for tuberculosis household contacts.  2. Integrated relevant non-tuberculosis and tuberculosis screening for tuberculosis household contacts will improve participation in tuberculosis screening.  3. Integrated relevant non-tuberculosis and tuberculosis screening for tuberculosis household contacts will improve uptake and completion of tuberculosis preventive therapy.  4. The interaction with the tuberculosis-affected household during tuberculosis screening is a good opportunity to provide non-tuberculosis screening to tuberculosis household contacts.  5. Offering tuberculosis screening that incorporates relevant non-tuberculosis screening and care will improve the health and wellbeing of tuberculosis household contacts.  6. The definition of relevant non-tuberculosis condition should be determined by a) the local disease burden (among the general population), b) the association of the conditions with tuberculosis risk or tuberculosis outcomes, c) the ability to provide screening using a simple, cheap and accurate test, with results available on the same day, d) the ability to provide affordable and effective treatment locally, e) the feasibility of integrating care for the condition with tuberculosis preventive therapy (TPT), during the period of TPT, f) the feasibility of continuing/sustaining care beyond the period of TPT if required and g) the acceptability of screening and care for the non-tuberculosis condition in the community. |
| During community-wide screening for tuberculosis in high-burden settings | 1. Community-wide systematic tuberculosis screening programmes are a good opportunity to screen for other conditions that are important to the community.  2. People participating in community-wide systematic tuberculosis screening in high-tuberculosis incidence settings should be offered screening for relevant non-tuberculosis conditions as part of the screening programme.  3. Programmes screening for relevant non-tuberculosis conditions during community wide systematic tuberculosis screening have a responsibility to ensure participants can access relevant non-tuberculosis care. |
| Evidence generation and funding | 1. Research measuring the selection, effectiveness and feasibility of offering non-tuberculosis services as part of routine tuberculosis care a) for people receiving tuberculosis treatment and b) as part of systematic screening for household contacts should be prioritised.  2. Domestic healthcare funding should support non-tuberculosis screening and provision of care for non-tuberculosis conditions amongst people receiving tuberculosis treatment.  3. International funding organisations should provide financial support for screening for non-tuberculosis conditions and provision of relevant non-tuberculosis services among a) people receiving tuberculosis treatment and b) people undergoing tuberculosis screening (for example tuberculosis household contacts and as part of community wide or targeted screening programmes). |

# References

1 World Health Organization. Global Tuberculosis Report 2023. Geneva: World Health Organization, 2023.

2 World Health Organization. Framework for collaborative action on tuberculosis and comorbidities. Geneva: World Health Organization, 2022.

3 Getahun H, Gunneberg C, Granich R, Nunn P. HIV infection-associated tuberculosis: the epidemiology and the response. *Clin Infect Dis* 2010; **50 Suppl 3**: S201-207.

4 World Health Organization. WHO policy on collaborative TB/HIV activities: guidelines for national programmes and other stakeholders. Geneva: World Health Organization, 2012 https://www.who.int/publications/i/item/9789241503006 (accessed Sept 5, 2024).

5 Olaru ID, Beliz Meier M, Mirzayev F, *et al.* Global prevalence of hepatitis B or hepatitis C infection among patients with tuberculosis disease: systematic review and meta-analysis. *EClinicalMedicine* 2023; **58**: 101938.

6 Baliashvili D, Blumberg H, Benkeser D, *et al.* Association of treated and untreated chronic hepatitis C with the incidence of active tuberculosis disease: a population-based cohort study. *Clin Infect Dis Off Publ Infect Dis Soc Am* 2022. DOI:10.1093/cid/ciac786.

7 Kim W, Lee SS, Lee C, *et al.* Hepatitis C and not Hepatitis B virus is a risk factor for anti-tuberculosis drug induced liver injury. *BMC Infect Dis* 2016; **16**. DOI:10.1186/s12879-016-1344-2.

8 Bushnell G, Stennis NL, Drobnik AM, *et al.* Characteristics and TB treatment outcomes in TB patients with viral hepatitis, New York City, 2000-2010. *Epidemiol Infect* 2015; **143**: 1972–81.

9 Jayes L, Haslam PL, Gratziou CG, *et al.* SmokeHaz: Systematic Reviews and Meta-analyses of the Effects of Smoking on Respiratory Health. *CHEST* 2016; **150**: 164–79.

10 Basu S, Stuckler D, Bitton A, Glantz SA. Projected effects of tobacco smoking on worldwide tuberculosis control: mathematical modelling analysis. *BMJ* 2011; **343**: d5506.

11 Lin HH, Ezzati M, Murray M. Tobacco Smoke, Indoor Air Pollution and Tuberculosis: A Systematic Review and Meta-Analysis. *PLOS Med* 2007; **4**: e20.

12 Wang MG, Huang WW, Wang Y, *et al.* Association between tobacco smoking and drug-resistant tuberculosis. *Infect Drug Resist* 2018; **11**: 873.

13 World Health Organization. Global tuberculosis report. Geneva, 2021.

14 Wagnew F, Eshetie S, Alebel A, Dessie G, Tesema C, Abajobir AA. Meta-analysis of the prevalence of tuberculosis in diabetic patients and its association with cigarette smoking in African and Asian countries. *BMC Res Notes* 2018; **11**: 298.

15 Hamada Y, Quartagno M, Law I, *et al.* Association of diabetes, smoking, and alcohol use with subclinical-to-symptomatic spectrum of tuberculosis in 16 countries: an individual participant data meta-analysis of national tuberculosis prevalence surveys. *eClinicalMedicine* 2023; **63**: 102191.

16 Adetifa IMO, Kendall L, Donkor S, *et al.* Mycobacterium tuberculosis Infection in Close Childhood Contacts of Adults with Pulmonary Tuberculosis is Increased by Secondhand Exposure to Tobacco. *Am J Trop Med Hyg* 2017; **97**: 429–32.

17 Chu AL, Lecca LW, Calderón RI, *et al.* Smoking Cessation in Tuberculosis Patients and the Risk of Tuberculosis Infection in Child Household Contacts. *Clin Infect Dis* 2021; **73**: 1500–6.

18 Leung CC, Yew WW, Chan CK, *et al.* Smoking adversely affects treatment response, outcome and relapse in tuberculosis. *Eur Respir J* 2015; **45**: 738–45.

19 Huynh N, Tariq S, Charron C, *et al.* Personalised multicomponent interventions for tobacco dependence management in low socioeconomic populations: a systematic review and meta-analysis. *J Epidemiol Community Health* 2022; **76**: 716–29.

20 Jeyashree K, Kathirvel S, Shewade HD, Kaur H, Goel S. Smoking cessation interventions for pulmonary tuberculosis treatment outcomes. *Cochrane Database Syst Rev* 2016; **2016**: CD011125.

21 Aryanpur M, Hosseini M, Masjedi MR, *et al.* A randomized controlled trial of smoking cessation methods in patients newly-diagnosed with pulmonary tuberculosis. *BMC Infect Dis* 2016; **16**: 369.

22 Whitehouse E, Lai J, Golub JE, Farley JE. A systematic review of the effectiveness of smoking cessation interventions among patients with tuberculosis. *Public Health Action* 2018; **8**: 37–49.

23 Fernandes L, Narvekar A, Lawande D. Efficacy of smoking cessation intervention delivered through mobile tele-counseling among smokers with tuberculosis in a Revised National Tuberculosis Control Program. *Indian J Tuberc* 2022; **69**: 207–12.

24 Bam TS, Aditama TY, Chiang C-Y, Rubaeah R, Suhaemi A. Smoking cessation and smokefree environments for tuberculosis patients in Indonesia-a cohort study. *BMC Public Health* 2015; **15**: 604.

25 Goel S, Kathiresan J, Singh P, Singh RJ. Effect of a brief smoking cessation intervention on adult tobacco smokers with pulmonary tuberculosis: A cluster randomized controlled trial from North India. *Indian J Public Health* 2017; **61**: S47–53.

26 Siddiquea BN, Islam MA, Bam TS, *et al.* High quit rate among smokers with tuberculosis in a modified smoking cessation programme in Dhaka, Bangladesh. *Public Health Action* 2013; **3**: 243–6.

27 Awaisu A, Nik Mohamed MH, Mohamad Noordin N, *et al.* The SCIDOTS Project: evidence of benefits of an integrated tobacco cessation intervention in tuberculosis care on treatment outcomes. *Subst Abuse Treat Prev Policy* 2011; **6**: 26.

28 Louwagie GMC, Okuyemi KS, Ayo-Yusuf OA. Efficacy of brief motivational interviewing on smoking cessation at tuberculosis clinics in Tshwane, South Africa: a randomized controlled trial. *Addiction* 2014; **109**: 1942–52.

29 Ramesh Kumar S, Dolla C, Vasantha M, Menon PA, Venkatesan G, Venkatesan P. Strategies for smoking cessation (pharmacologic intervention versus enhanced motivation vs. standard motivation) in TB patients under treatment in the RNTCP, India - A cluster - Randomized trial. *Indian J Tuberc* 2020; **67**: 8–14.

30 Khan MT, Zaheer S, Amar W, Shafique K. Effect of smoking cessation interventions on abstinence and tuberculosis treatment outcomes among newly diagnosed patients: a randomized controlled trial. *Microbiol Spectr* 2024; : e0387823.

31 Purushothama J, Badiger S, Olickal JJ, Kunkulol R, Kumar N, D’Souza N. Effectiveness of Nicotine Replacement Therapy on Smoking Cessation and Reduction Among Pulmonary Tuberculosis Patients - A Randomized Controlled Trial. *Int J Prev Med* 2023; **14**: 33.

32 Sharma SK, Mohan A, Singh AD, *et al.* Impact of nicotine replacement therapy as an adjunct to anti-tuberculosis treatment and behaviour change counselling in newly diagnosed pulmonary tuberculosis patients: an open-label, randomised controlled trial. *Sci Rep* 2018; **8**: 8828.

33 Siddiqi K, Keding A, Marshall A-M, *et al.* Effect of quitting smoking on health outcomes during treatment for tuberculosis: secondary analysis of the TB & Tobacco Trial. *Thorax* 2022; **77**: 74–8.

34 World Health Organization, International Union against Tuberculosis and Lung Disease. A WHO / the Union monograph on TB and tobacco control : joining efforts to control two related global epidemics. 2007; : 96.

35 Elsey H, Al Azdi Z, Regmi S, *et al.* Scaling up tobacco cessation within TB programmes: findings from a multi-country, mixed-methods implementation study. *Health Res Policy Syst* 2022; **20**: 43.

36 World Health Organization. Global status report on alcohol and health and treatment of substance use disorders. Geneva: World Health Organization, 2024.

37 Lönnroth K, Williams BG, Stadlin S, Jaramillo E, Dye C. Alcohol use as a risk factor for tuberculosis-a systematic review. *BMC Public Health* 2008; **8**. DOI:10.1186/1471-2458-8-289.

38 Rehm J, Samokhvalov AV, Neuman MG, *et al.* The association between alcohol use, alcohol use disorders and tuberculosis (TB). A systematic review. *BMC Public Health* 2009; **9**: 1–12.

39 Imtiaz S, Shield KD, Roerecke M, Samokhvalov AV, Lönnroth K, Rehm J. Alcohol consumption as a risk factor for tuberculosis: meta-analyses and burden of disease. *Eur Respir J* 2017; **50**: 1–13.

40 Simou E, Britton J, Leonardi-Bee J. Alcohol consumption and risk of tuberculosis: A systematic review and meta-analysis. *Int J Tuberc Lung Dis* 2018; **22**: 1277–85.

41 Stop TB Partnership. Country Profile. Interact. Ctry. Dashboards. 2024. https://dashboards.stoptb.org/country-profile.html (accessed Sept 5, 2024).

42 Ragan EJ, Kleinman MB, Sweigart B, *et al.* The impact of alcohol use on tuberculosis treatment outcomes: a systematic review and meta-analysis HHS Public Access. *Int J Tuberc Lung Dis* 2020; **24**: 73–82.

43 Degenhardt L, Peacock A, Colledge S, *et al.* Global prevalence of injecting drug use and sociodemographic characteristics and prevalence of HIV, HBV, and HCV in people who inject drugs: a multistage systematic review. *Lancet Glob Health* 2017; **5**: e1192–207.

44 Getahun H, Gunneberg C, Sculier D, Verster A, Raviglione M. Tuberculosis and HIV in people who inject drugs: Evidence for action for tuberculosis, HIV, prison and harm reduction services. *Curr Opin HIV AIDS* 2012; **7**: 345–53.

45 World Health Organization. Integrating collaborative TB and HIV services within a comprehensive package of care for people who inject drugs: consolidated guidelines. Geneva: World Health Organization, 2016 https://iris.who.int/handle/10665/204484 (accessed Sept 6, 2024).

46 Howlett P, Mousa H, Said B, *et al.* Silicosis, tuberculosis and silica exposure among artisanal and small-scale miners: A systematic review and modelling paper. *PLOS Glob Public Health* 2023; **3**: e0002085.

47 Howlett P, Gan J, Lesosky M, Feary J. Relationship between cumulative silica exposure and silicosis: a systematic review and dose-response meta-analysis. *Thorax* 2024; **79**: 934–42.

48 Byrne AL, Marais BJ, Mitnick CD, Lecca L, Marks GB. Tuberculosis and chronic respiratory disease: a systematic review. *Int J Infect Dis* 2015; **32**: 138–46.

49 Taylor J, Bastos ML, Lachapelle-Chisholm S, Mayo NE, Johnston J, Menzies D. Residual respiratory disability after successful treatment of pulmonary tuberculosis: a systematic review and meta-analysis. *EClinicalMedicine* 2023; **59**: 101979.

50 Burney P, Patel J, Minelli C, *et al.* Prevalence and Population-Attributable Risk for Chronic Airflow Obstruction in a Large Multinational Study. *Am J Respir Crit Care Med* 2021; **203**: 1353–65.

51 van Kampen SC, Jones R, Kisembo H, *et al.* Chronic Respiratory Symptoms and Lung Abnormalities Among People With a History of Tuberculosis in Uganda: A National Survey. *Clin Infect Dis Off Publ Infect Dis Soc Am* 2019; **68**: 1919–25.

52 Ehrlich R, Akugizibwe P, Siegfried N, Rees D. The association between silica exposure, silicosis and tuberculosis: a systematic review and meta-analysis. *BMC Public Health* 2021; **21**: 953.

53 Churchyard GJ, Kleinschmidt I, Corbett EL, Murray J, Smit J, De Cock KM. Factors associated with an increased case-fatality rate in HIV-infected and non-infected South African gold miners with pulmonary tuberculosis. *Int J Tuberc Lung Dis* 2000; **4**: 705–12.

54 Corbett EL, Churchyard GJ, Clayton TC, *et al.* HIV infection and silicosis: the impact of two potent risk factors on the incidence of mycobacterial disease in South African miners. *AIDS* 2000; **14**: 2759–68.

55 Hamada Y, Fong CJ, Copas A, Hurst JR, Rangaka MX. Risk for development of active tuberculosis in patients with chronic airway disease-a systematic review of evidence. *Trans R Soc Trop Med Hyg* 2022; **116**: 390–8.

56 World Health Organization. WHO consolidated guidelines on tuberculosis. Module 2: screening – systematic screening for tuberculosis disease. Geneva: World Health Organization, 2021.

57 World Health Organization. WHO consolidated guidelines on tuberculosis: module 1: prevention: tuberculosis preventive treatment. Geneva: World Health Organization, 2020 https://www.who.int/publications/i/item/9789240001503 (accessed Sept 6, 2024).

58 World Health Organization. mhGAP Mental Health Gap Action Programme. Geneva: World Health Organization, 2008 https://www.who.int/publications/i/item/9789241596206 (accessed Sept 6, 2024).

59 Duko B, Bedaso A, Ayano G. The prevalence of depression among patients with tuberculosis: a systematic review and meta-analysis. *Ann Gen Psychiatry* 2020; **19**: 30.

60 Janse Van Rensburg A, Dube A, Curran R, *et al.* Comorbidities between tuberculosis and common mental disorders: A scoping review of epidemiological patterns and person-centred care interventions from low-to-middle income and BRICS countries. *Infect Dis Poverty* 2020; **9**: 1–18.

61 Alene KA, Wangdi K, Colquhoun S, *et al.* Tuberculosis related disability: a systematic review and meta-analysis. *BMC Med* 2021; **19**: 203.

62 Patwal R, Sachdeva A, Bhaskarapillai B, Arasappa R, Muliyala KP, Desai G. Prevalence of suicidal ideations and suicide attempts in patients with tuberculosis: A systematic review and meta-analysis. *J Psychosom Res* 2023; **167**: 111171.

63 Koyanagi A, Vancampfort D, Carvalho AF, *et al.* Depression comorbid with tuberculosis and its impact on health status: cross-sectional analysis of community-based data from 48 low- and middle-income countries. *BMC Med* 2017; **15**: 209.

64 Hayward S, Deal A, Rustage K, *et al.* A systematic review of the association between mental health and tuberculosis disease risk. *Eur J Public Health* 2021; **31**: ckab164.885.

65 Oh KH, Choi H, Kim EJ, Kim HJ, Cho SI. Depression and risk of tuberculosis: a nationwide population-based cohort study. *Int J Tuberc Lung Dis Off J Int Union Tuberc Lung Dis* 2017; **21**: 804–9.

66 Yan S, Zhang S, Tong Y, Yin X, Lu Z, Gong Y. Nonadherence to Antituberculosis Medications: The Impact of Stigma and Depressive Symptoms. *Am J Trop Med Hyg* 2018; **98**: 262–5.

67 Lee G, Scuffell J, Galea JT, *et al.* Impact of mental disorders on active TB treatment outcomes: a systematic review and meta-analysis. *Int J Tuberc Lung Dis* 2020; **24**: 1279–84.

68 Ruiz-Grosso P, Cachay R, de la Flor A, Schwalb A, Ugarte-Gil C. Association between tuberculosis and depression on negative outcomes of tuberculosis treatment: A systematic review and meta-analysis. *PloS One* 2020; **15**: e0227472.

69 Gautam S, Shrestha N, Mahato S, Nguyen TPA, Mishra SR, Berg-Beckhoff G. Diabetes among tuberculosis patients and its impact on tuberculosis treatment in South Asia: a systematic review and meta-analysis. *Sci Rep* 2021; **11**: 2113.

70 Sweetland AC, Kritski A, Oquendo MA, *et al.* Addressing the tuberculosis-depression syndemic to end the tuberculosis epidemic. *Int J Tuberc Lung Dis* 2017; **21**: 852–61.

71 Walker IF, Baral SC, Wei X, *et al.* Multidrug-resistant tuberculosis treatment programmes insufficiently consider comorbid mental disorders. *Int J Tuberc Lung Dis* 2017; **21**: 603–9.

72 Sweetland AC, Galea J, Shin SS, *et al.* Integrating tuberculosis and mental health services: global receptivity of national tuberculosis program directors. *Int J Tuberc Lung Dis* 2019; **23**: 600–5.

73 Sorsdahl K, Naledi T, Lund C, *et al.* Integration of mental health counselling into chronic disease services at the primary health care level: Formative research on dedicated versus designated strategies in the Western Cape, South Africa. *J Health Serv Res Policy* 2021; **26**: 172–9.

74 Jarde A, Siqueira N, Afaq S, *et al.* Addressing TB multimorbidity in policy and practice: An exploratory survey of TB providers in 27 high-TB burden countries. *PLOS Glob Public Health* 2022; **2**: e0001205.

75 Farooq S, Tunmore J, Comber R. Pharmacological or non-pharmacological interventions for treatment of common mental disorders associated with Tuberculosis: A systematic review. *Chron Respir Dis* 2021; **18**: 14799731211003937.

76 World Health Organization. WHO operational handbook on tuberculosis Module 6: Tuberculosis and comorbidities Mental health conditions. Geneva: World Health Organization, 2023.

77 Simms V, Weiss HA, Chinoda S, *et al.* Peer-led counselling with problem discussion therapy for adolescents living with HIV in Zimbabwe: A cluster-randomised trial. *PLOS Med* 2022; **19**: e1003887.

78 Sun H, Saeedi P, Karuranga S, *et al.* IDF Diabetes Atlas: Global, regional and country-level diabetes prevalence estimates for 2021 and projections for 2045. *Diabetes Res Clin Pract* 2022; **183**: 109119.

79 Noubiap JJ, Nansseu JR, Nyaga UF, *et al.* Global prevalence of diabetes in active tuberculosis: a systematic review and meta-analysis of data from 2·3 million patients with tuberculosis. *Lancet Glob Health* 2019; **7**: e448–60.

80 Lee M-R, Huang Y-P, Kuo Y-T, *et al.* Diabetes Mellitus and Latent Tuberculosis Infection: A Systemic Review and Metaanalysis. *Clin Infect Dis* 2017; **64**: 719–27.

81 Martinez L, Zhu L, Castellanos ME, *et al.* Glycemic Control and the Prevalence of Tuberculosis Infection: A Population-based Observational Study. *Clin Infect Dis* 2017; **65**: 2060–8.

82 Al-Rifai RH, Pearson F, Critchley JA, Abu-Raddad LJ. Association between diabetes mellitus and active tuberculosis: A systematic review and meta-analysis. *PloS One* 2017; **12**. DOI:10.1371/journal.pone.0187967.

83 Hayashi S, Chandramohan D. Risk of active tuberculosis among people with diabetes mellitus: systematic review and meta-analysis. *Trop Med Int Health* 2018; **23**: 1058–70.

84 Foe-Essomba JR, Kenmoe S, Tchatchouang S, *et al.* Diabetes mellitus and tuberculosis, a systematic review and meta-analysis with sensitivity analysis for studies comparable for confounders. *PloS One* 2021; **16**: e0261246.

85 Obels I, Ninsiima S, Critchley JA, Huangfu P. Tuberculosis risk among people with diabetes mellitus in Sub-Saharan Africa: A systematic review. *Trop Med Int Health* 2022; **27**: 369–86.

86 Franco J, Metzendorf M, Risso A, *et al.* Diabetes as a risk factor for tuberculosis disease. *Cochrane Database Syst Rev* 2024; **8**. DOI:10.1002/14651858.CD016013.pub2.

87 Lee PH, Fu H, Lai TC, Chiang CY, Chan CC, Lin HH. Glycemic Control and the Risk of Tuberculosis: A Cohort Study. *PLoS Med* 2016. DOI:10.1371/journal.pmed.1002072.

88 Chen Z, Liu Q, Song R, *et al.* The association of glycemic level and prevalence of tuberculosis: a meta-analysis. *BMC Endocr Disord* 2021; **21**: 123.

89 Mendenhall E, Norris SA. When HIV is ordinary and diabetes new: remaking suffering in a South African township. *Glob Public Health* 2015; **10**: 449–62.

90 Baker MA, Harries AD, Jeon CY, *et al.* The impact of diabetes on tuberculosis treatment outcomes: A systematic review. *BMC Med* 2011; **9**: 1–15.

91 Huangfu P, Ugarte-Gil C, Golub J, Pearson F, Critchley J. The effects of diabetes on tuberculosis treatment outcomes: an updated systematic review and meta-analysis. *Int J Tuberc Lung Dis* 2019; **23**: 783–96.

92 Pan SC, Ku CC, Kao D, Ezzati M, Fang CT, Lin HH. Effect of diabetes on tuberculosis control in 13 countries with high tuberculosis: a modelling study. *Lancet Diabetes Endocrinol* 2015; **3**: 323–30.

93 Kubjane M, Berkowitz N, Goliath R, Levitt NS, Wilkinson RJ, Oni T. Tuberculosis, Human Immunodeficiency Virus, and the Association With Transient Hyperglycemia in Periurban South Africa. *Clin Infect Dis* 2020; **71**: 1080–8.

94 World Health Organization, International Union against Tuberculosis and Lung Disease. Collaborative framework for care and control of tuberculosis and diabetes. Geneva: World Health Organization, 2011.

95 Jackson-Morris A, Masyuko S, Morrell L, Kataria I, Kocher EL, Nugent R. Tackling syndemics by integrating infectious and noncommunicable diseases in health systems of low- and middle-income countries: A narrative systematic review. *PLOS Glob Public Health* 2024; **4**: e0003114.

96 Mensah GA, Fuster V, Murray CJL, *et al.* Global Burden of Cardiovascular Diseases and Risks, 1990-2022. *J Am Coll Cardiol* 2023; **82**: 2350–473.

97 López‐López JP, Posada‐Martínez EL, Saldarriaga C, *et al.* Tuberculosis and the Heart. *J Am Heart Assoc Cardiovasc Cerebrovasc Dis* 2021; **10**: e019435.

98 Byrne AL, Marais BJ, Mitnick CD, *et al.* Feasibility and yield of screening for non-communicable diseases among treated tuberculosis patients in Peru. *Int J Tuberc Lung Dis* 2018; **22**: 86–92.

99 Basham CA, Smith SJ, Romanowski K, Johnston JC. Cardiovascular morbidity and mortality among persons diagnosed with tuberculosis: A systematic review and meta-analysis. *PloS One* 2020; **15**: e0235821.

100 World Health Organization. Guideline: nutritional care and support for patients with tuberculosis. Geneva: World Health Organization, 2013 https://apps.who.int/iris/handle/10665/94836.

101 Global Nutrition Report. Global Nutrition Report 2022. 2022 https://globalnutritionreport.org/reports/2022-global-nutrition-report/executive-summary/#note-jk1_NK5Av (accessed Oct 1, 2024).

102 Carwile ME, Hochberg NS, Sinha P. Undernutrition is feeding the tuberculosis pandemic: A perspective. *J Clin Tuberc Mycobact Dis* 2022; **27**: 100311.

103 Franco JV, Bongaerts B, Metzendorf M-I, *et al.* Undernutrition as a risk factor for tuberculosis disease. *Cochrane Database Syst Rev* 2024. DOI:10.1002/14651858.CD015890.pub2.

104 Lönnroth K, Williams BG, Cegielski P, Dye C. A consistent log-linear relationship between tuberculosis incidence and body mass index. *Int J Epidemiol* 2010; **39**: 149–55.

105 Park J, Yoon JH, Ki HK, Eun Y, Han K, Kim H. Association of duration of undernutrition with occurrence of tuberculosis. *BMC Public Health* 2022; **22**: 2392.

106 Min J, Kim JS, Kim HW, *et al.* Effects of underweight and overweight on mortality in patients with pulmonary tuberculosis. *Front Public Health* 2023; **11**: 1236099.

107 Sinha P, Ponnuraja C, Gupte N, *et al.* Impact of Undernutrition on Tuberculosis Treatment Outcomes in India: A Multicenter, Prospective, Cohort Analysis. *Clin Infect Dis* 2023; **76**: 1483–91.

108 Khan A, Sterling TR, Reves R, Vernon A, Horsburgh CR. Lack of weight gain and relapse risk in a large tuberculosis treatment trial. *Am J Respir Crit Care Med* 2006; **174**: 344–8.

109 Cegielski JP, McMurray DN. The relationship between malnutrition and tuberculosis: evidence from studies in humans and experimental animals. *Int J Tuberc Lung Dis* 2004; **8**: 286–98.

110 Bhargava A, Pai M, Bhargava M, Marais BJ, Menzies D. Can Social Interventions Prevent Tuberculosis? *Am J Respir Crit Care Med* 2012; **186**: 442–9.

111 Sinha P, Ezhumalai K, Du X, *et al.* Undernourished Household Contacts Are at Increased Risk of Tuberculosis (TB) Disease, but not TB Infection— a Multicenter Prospective Cohort Analysis. *Clin Infect Dis* 2024; : ciae149.

112 Baazim H, Antonio-Herrera L, Bergthaler A. The interplay of immunology and cachexia in infection and cancer. *Nat Rev Immunol* 2022; **22**: 309–21.

113 Management of severe acute malnutrition in individuals with active tuberculosis. https://www.who.int/tools/elena/interventions/sam-tuberculosis (accessed March 25, 2025).

114 Bhargava A, Bhargava M, Meher A, *et al.* Nutritional support for adult patients with microbiologically confirmed pulmonary tuberculosis: outcomes in a programmatic cohort nested within the RATIONS trial in Jharkhand, India. *Lancet Glob Health* 2023; **0**. DOI:10.1016/S2214-109X(23)00324-8.

115 World Health Organization. Disability. 2024. https://www.who.int/health-topics/disability (accessed Oct 18, 2024).

116 World Health Organization. Policy brief on tuberculosis-associated disability. Geneva: World Health Organization, 2023.

117 World Health Organization. Interim policy on collaborative TB/HIV activities. .

118 World Health Organization, World Health Organization. Treatment of tuberculosis: guidelines. 2010. https://iris.who.int/handle/10665/44165 (accessed Oct 17, 2024).

119 World Health Organization. Implementing the end TB strategy: the essentials. Geneva: World Health Organization, 2015 https://iris.who.int/handle/10665/206499 (accessed Oct 7, 2024).

120 United Nations General Assembly. Political declaration of the High-Level Meeting of the United Nations General Assembly on the fight against tuberculosis. United Nations, 2018.

121 Hamada Y, Quartagno M, Malik F, *et al.* Prevalence of non-communicable diseases among household contacts of people with tuberculosis: A systematic review and individual participant data meta-analysis. *Trop Med Int Health* 2024; **29**: 768–80.

122 Scott P, Elsayedkarar M, Marambire E, *et al.* HIV Testing During Systematic Screening for Tuberculosis Among Household Contacts Inhigh-Tuberculosis Burden Settings: A Systematic Review and Meta-Analysis. 2025; published online Feb 25. DOI:10.2139/ssrn.5151057.

123 Hamada Y, Quartagno M, Law I, *et al.* Tobacco smoking clusters in households affected by tuberculosis in an individual participant data meta-analysis of national tuberculosis prevalence surveys: Time for household-wide interventions? *PLOS Glob Public Health* 2024; **4**: e0002596.

124 Kim Soyeon, Hesseling AC, Wu XingYe, *et al.* Factors associated with prevalent Mycobacterium tuberculosis infection and disease among adolescents and adults exposed to rifampin-resistant tuberculosis in the household. *PLoS ONE* 2023; **18**. DOI:10.1371/journal.pone.0283290.

125 Lasebikan VO, Ige OM. Alcohol use disorders in multidrug resistant tuberculosis (MDR-TB) patients and their non-tuberculosis family contacts in Nigeria. *Pan Afr Med J* 2020; **1**: 321.

126 Fuchs A, Tufa TB, Pfäfflin F, *et al.* Risk-adjusted active tuberculosis case finding strategy in central Ethiopia. *IJID Reg* 2022; **3**: 196–203.

127 Htet KKK, Liabsuetrakul T, Thein S, McNeil EB, Chongsuvivatwong V. Improving detection of tuberculosis among household contacts of index tuberculosis patients by an integrated approach in Myanmar: A cross-sectional study. *BMC Infect Dis* 2018; **18**: 1–8.

128 Gyawali N, Gurung R, Poudyal N, *et al.* Tobacco and alcohol: the relation to pulmonary tuberculosis in household contacts. *Nepal Med Coll J* 2013; **15**: 125–8.

129 Paradkar M, Padmapriyadarsini C, Jain D, *et al.* Tuberculosis preventive treatment should be considered for all household contacts of pulmonary tuberculosis patients in India. *PLoS ONE* 2020; **15**: e0236743.

130 Shivakumar SVBY, Chandrasekaran P, Kumar AMV, *et al.* Diabetes and pre-diabetes among household contacts of tuberculosis patients in India: is it time to screen them all? *Int J Tuberc Lung Dis* 2018; **22**: 686–94.

131 Bhargava A, Bhargava M, Meher A, *et al.* Nutritional supplementation to prevent tuberculosis incidence in household contacts of patients with pulmonary tuberculosis in India (RATIONS): a field-based, open-label, cluster-randomised, controlled trial. *The Lancet* 2023; **402**: 627–40.

132 Shang Z. Use of Delphi in health sciences research: A narrative review. *Medicine (Baltimore)* 2023; **102**: e32829.

133 Khan MS, Rego S, Rajal JB, *et al.* Mitigating the impact of COVID-19 on tuberculosis and HIV services: A cross-sectional survey of 669 health professionals in 64 low and middle-income countries. *PLOS ONE* 2021; **16**: e0244936.

134 World Health Organization. Monitoring the building blocks of health systems: a handbook of indicators and their measurement strategies. Geneva: World Health Organization, 2010 https://iris.who.int/handle/10665/258734 (accessed Sept 24, 2024).

135 Round 2 TB Delphi Survey. 2024 https://www.youtube.com/watch?v=lVPKIKCBPN0 (accessed Sept 24, 2024).
